# Evolution of the Insertion-Deletion Mutation Rate Across the Tree of Life

**DOI:** 10.1534/g3.116.030890

**Published:** 2016-06-15

**Authors:** Way Sung, Matthew S. Ackerman, Marcus M. Dillon, Thomas G. Platt, Clay Fuqua, Vaughn S. Cooper, Michael Lynch

**Affiliations:** * Department of Bioinformatics and Genomics, University of North Carolina at Charlotte, North Carolina 28223; † Department of Biology, Indiana University, Bloomington, Indiana 47405; ‡ Microbiology Graduate Program, University of New Hampshire, Durham, New Hampshire 03824; § Division of Biology, Kansas State University, Manhattan, Kansas 66506; ** Department of Microbiology and Molecular Genetics, University of Pittsburgh School of Medicine, Pennsylvania 15219

**Keywords:** insertion-deletion mutation rate, mutation-rate evolution, drift barrier, mutation accumulation

## Abstract

Mutations are the ultimate source of variation used for evolutionary adaptation, while also being predominantly deleterious and a source of genetic disorders. Understanding the rate of insertion-deletion mutations (indels) is essential to understanding evolutionary processes, especially in coding regions, where such mutations can disrupt production of essential proteins. Using direct estimates of indel rates from 14 phylogenetically diverse eukaryotic and bacterial species, along with measures of standing variation in such species, we obtain results that imply an inverse relationship of mutation rate and effective population size. These results, which corroborate earlier observations on the base-substitution mutation rate, appear most compatible with the hypothesis that natural selection reduces mutation rates per effective genome to the point at which the power of random genetic drift (approximated by the inverse of effective population size) becomes overwhelming. Given the substantial differences in DNA metabolism pathways that give rise to these two types of mutations, this consistency of results raises the possibility that refinement of other molecular and cellular traits may be inversely related to species-specific levels of random genetic drift.

Mutations are a double-edged sword in all organisms, constituting the ultimate source of variation used for evolutionary adaptation, while also being predominantly deleterious and a source of genetic disorders. Hence, researchers have long sought the primary factors governing mutation-rate evolution. Some have argued that the mutation rate of an organism reflects a balance between the deleterious effect of mutations and physiological limitations, with further refinement of replication fidelity limiting the speed of DNA synthesis necessary for efficient daughter-cell production (Drake 1991; Sniegowski *et al.* 2000). However, replication fidelity can be improved without a significant decrease in doubling time (Loh *et al.* 2010), and prokaryotes undergo high cell-division rates and have low mutation rates (Drake 1991; Lynch 2010), suggesting that replication fidelity does not limit the rate of daughter-cell production. Furthermore, because there is no negative correlation between cell-division rate and genome size (Mira *et al.* 2001; Vieira-Silva *et al.* 2010), and the reverse may even be true in bacteria (Lynch and Marinov 2015), cell-division rates do not appear to be limited by the amount of DNA synthesized. Thus, alternative forces may govern mutation-rate evolution.

A general relationship describing mutation-rate variation was proposed by Drake *et al.* (1998), who suggested that the mutation rate per nucleotide site scales inversely with genome size in bacteria and unicellular eukaryotes, such that there is a constant ∼0.003 mutations per haploid genome per cell division. However, as direct estimates of mutation rates for additional organisms became available, the general relationship between genome size and mutation rate became less apparent, even when scaled to the number of cell divisions per generation in multicellular species (Lynch 2010).

In a previous analysis, we found a relationship between the base-substitution mutation rate per site per generation (*u_bs_*) multiplied by the amount of functional DNA in a genome (*G_e_*, approximated by proteome size), and the power of random genetic drift, which is inversely proportional to the effective population size (*N_e_*) (Sung *et al.* 2012a). Because mutations are generally deleterious, this finding suggested that selection operates to reduce genome-wide mutation rates by refining DNA replication fidelity and repair until further improvements are too inconsequential to overcome the power of random genetic drift (Sniegowski and Raynes 2013). This result is consistent with the drift-barrier hypothesis (DBH), which proposes that natural selection operates to improve molecular and cellular traits until the selective advantage of a beneficial mutation refining the trait is so miniscule that the probability of it being fixed is essentially the same as that for neutral mutations (Lynch 2011; Sung *et al.* 2012a).

While the negative correlation between *u_bs_G_e_* and *N*_e_ is consistent with expectations from population-genetic theory, there is a potential issue of circularity when correlating these factors, as the estimation of *N*_e_ relies indirectly on the estimation of *u_bs_* (Sung *et al.* 2012a). Although we presented an analysis suggesting that the correlated parameters are not likely to be the primary factor in the observed relationship (Sung *et al.* 2012a), and provide another one here (Supplemental Material, File S1), a more independent analysis is desirable, and, given the amount of data that has accumulated, it is time to go beyond a study that simply considers base-substitution mutations. Here, we present the rate of insertion-deletion mutation (indel) events (*u_id_*) per site per generation across eight eukaryotic and seven bacterial species, while also providing genome-wide estimates of *u_bs_* and *u_id_* from three new bacterial mutation-accumulation studies. These data continue to support a negative correlation between the genome-wide mutation rate and *N*_e_.

The DBH postulates that genetic drift determines the limit of adaptive molecular refinement that can be achieved for any trait, including those that determine the rate of indels. Indels are a class of mutations separate from base substitutions, differing in how they originate. Indels generally arise from strand slippage or double-strand breaks, whereas base-substitution mutations originate primarily from base misincorporation or biochemical alteration. Furthermore, there are major differences in how the two mutation types are repaired. Base-substitution mutations are often reversed by enzymes such as DNA photolyases and alkyl transferases, which do not require DNA incision and synthesis (Sancar *et al.* 2004), or are identified by glycosylases in base-excision repair (BER) pathways, and repaired by incision and DNA-gap filling (Krokan and Bjoras 2013). On the other hand, indel mutations are not surveyed by BER, but are repaired primarily by nucleotide-excision repair (NER), which has broad substrate specificity, and is used to excise bulky lesions arising from the insertion or deletion of nucleotides (Morita *et al.* 2010). Although the mismatch-repair (MMR) pathway can operate on both base-substitution mutations and indels, MMR-deficient strains of *Escherichia coli* and *Caenorhabditis elegans* exhibit a significantly greater elevation of the indel mutation rate relative to that for base substitutions, providing further evidence for the differential treatment of mutation types by DNA-repair pathways (Denver *et al.* 2005; Lee *et al.* 2012). Furthermore, depending on the type of mismatch and local sequence context, the error rates of different polymerases are highly variable between indel and base-substitution mutations (McCulloch and Kunkel 2008; Kunkel 2009; Sung *et al.* 2015). In summary, because the enzymes influencing base-substitution and indel mutation rates differ (and shared enzymes differ in the spectrum of repaired premutations), a focus on the indel mutation rate provides a means of testing the validity of the DBH that is substantially independent biologically (and essentially fully independent in terms of investigator sampling) of that used to extrapolate measures of the power of random genetic drift.

Selection operates to refine DNA replication fidelity and repair when the genome-wide deleterious load confers a discernable fitness disadvantage on an organism (Kimura 1967, 1983; Lynch 2010), and the contributions of indel and base substitution mutations to genome-wide deleterious load differ in two ways. First, the effects of base substitutions in coding regions are highly variable (Eyre-Walker and Keightley 2007), and some base substitutions may not have any effect on organismal fitness, which may create some uncertainties in quantifying the effective genome size (*G_e_*), thereby reducing the correlation observed between *u_bs_G_e_* and *N*_e_ (Sung *et al.* 2012a). On the other hand, most indel mutations that arise in protein-coding genes will generate a frame-shift mutation, interfering with gene function, and having a direct effect on organismal fitness. Because such indels are generally deleterious, selection is then expected to more efficiently fine tune the rate at which indels arise, and, if the DBH holds true, this should yield a close correlation between *u_id_G_e_* and *N*_e_. Second, base-substitutions are generally limited to single nucleotides, while indels may involve many base pairs. Although this might suggest that indels have a larger effect than base substitutions, single-base pair indels and gene-sized indels both result in gene disruption, thus generating more similar fitness effects regardless of the indel length. In fact, single base-pair indels in coding DNA may generate malformed gene products that require degradation, which might be even more harmful than entire gene deletions. Because the number of indel events, and not the size of indels, determines the genome-wide deleterious burden, we define the parameter *u_id_* to be the number of indel mutation events per site per generation, and use this parameter to test the DBH.

## Materials and Methods

To examine the effect of genetic drift on mutation-rate evolution, it is necessary to derive accurate estimates of the mutation rate and genetic diversity across phylogenetically diverse organisms. Whole-genome sequencing (WGS) has greatly improved our ability to estimate such parameters. Highly accurate measurements of *u_bs_* and *u_id_* can be obtained through WGS of mutation-accumulation (MA) lines, in which repeated single-organism bottlenecking minimizes the efficiency of selection, allowing for the accumulation of all but the most deleterious mutations (Lynch *et al.* 2008; Denver *et al.* 2009; Ossowski *et al.* 2010; Sung *et al.* 2012a, 2012b, 2015; Schrider *et al.* 2013). Along with data from prior MA studies, this study contains MA data from four new MA experiments. For new bacterial MA species, ∼100 independent MA lines were initiated from a single founder colony. The new strains used were as follows: *Agrobacterium tumefaciens* str. C58, *Staphylococcus epidermidis* ATCC 12228, and *Vibrio cholerae* 2740-80.

Depending on the speed of growth, a single colony from each MA line was isolated and transferred to a fresh plate every 1–3 d over the course of the experiment. The bottlenecking process ensures that mutations accumulate in an effectively neutral fashion (Kibota and Lynch 1996). After each transfer, the original plate was retained as a backup plate at 4°. If the destination plate was contaminated, or if a single colony could not be picked, a single colony was transferred from the last 4° backup plate.

To estimate the generation times that occurred between each transfer, every 2 wk, an entire colony from five randomly selected MA lines was transferred to 1 × PBS saline buffer. These were vortexed, serially diluted, and replated. Cell density was calculated from viable cell counts in both the growth conditions used throughout the bottleneck process as well as growth conditions at 4°. The total number of generations for each MA line was calculated by the average number of cell divisions per transfer multiplied by the total number of transfers. If backup plates were used, the average number of cell divisions at 4° was used in place of the average number of cell divisions per bottleneck at standard growth temperatures.

The average number of cell divisions across the MA are as follows (Dataset S1): *A. tumefaciens*, 5819; *Bacillus subtilis*, 5078 (Sung *et al.* 2015); *E. coli*, 4246 (Lee *et al.* 2012); *Mesoplasma florum*, 2351 (Sung *et al.* 2012a); *S. epidermidis*, 7170, and *V. cholerae*, 6453. The average number of generations used for reanalysis of the *C. elegans* MA study was 250 (Denver *et al.* 2009) (Dataset S2).

DNA extraction of MA lines was done using the wizard DNA extraction kit (Promega) or lysis media (CTAB or SDS) followed by phenol/chloroform extractions to Illumina library standards. Then, 101-bp paired-end Illumina (Illumina Hi-Seq platform) sequencing was applied to randomly selected MA lines of *A. tumefaciens*, *S. epidermidis*, and *V. cholerae*. Each MA line was sequenced to a coverage depth of ∼100 ×, with an average library fragment size (distance between paired-end reads) of ∼175 bp. The paired-end reads for each MA line were individually mapped against the reference genome (assembly and annotation available from the National Center for Biotechnology Information, https://www.ncbi.nlm.nih.gov) using two separate alignment algorithms: BWA v0.7.4 (Li and Durbin 2009) and NOVOALIGN v2.08.02 (available at www.novocraft.com). The resulting pileup files were converted to SAM format using SAMTOOLS v0.1.18 (Li *et al.* 2009). Using in-house perl scripts, the alignment information was further parsed to generate forward and reverse mapping information at each site, resulting in a configuration of eight numbers for each line (A, a, C, c, G, g, T, and t), corresponding to the number of reads mapped at each genomic position in the reference sequence. A separate file was also generated to display sites that had indel calls from the two alignment algorithms. Mutation calling was performed using a consensus method (Lynch *et al.* 2008; Denver *et al.* 2009; Ossowski *et al.* 2010; Lee *et al.* 2012; Sung *et al.* 2012a, 2012b, 2015).

A random subset of base-substitutions mutations called using these methods have been previously validated in *E. coli* and *B. subtilis* MA lines using fluorescent sequencing technology at the Indiana Molecular Biology Institute at Indiana University (Lee *et al.* 2012; Sung *et al.* 2015) (Dataset S3).

To verify indel mutations, we designed 38 primer sets to PCR amplify 300–500 bp regions surrounding the putative indel mutation in the *B. subtilis* MA lines (Dataset S4). All 29/29 short indels (< 10 bp) were directly confirmed using standard fluorescent sequencing technology. Two out of nine large indels (> 10 bp) were confirmed through sizing of the PCR product on gel electrophoresis. The remaining seven large indels did not amplify. For all cases, the indel was also confirmed to be absent in one other line without the mutation.

To calculate the base-substitution mutation rate per cell division for each line, we used the following equation:

$$u_{bs}\;=\;\frac{m}{nT},$$

where *u_bs_* is the base-substitution mutation rate (per nucleotide site per generation), *m* is the number of observed base substitutions, *n* is the number of nucleotide sites analyzed, and *T* is the number of generations that occurred in the mutation-accumulation study. The SE for an individual line is calculated using (Denver *et al.* 2004, 2009):

$$SE_{\bar{x}}\;=\;\sqrt{\frac{u_{bs}}{nT}}.$$

The total SE of base-substitution mutation rate is given by the SD of the mutation rates across all lines (*s*) divided by the square root of the number of lines analyzed (*N*).

$$SE_{pooled}\;=\;\frac{s}{\sqrt{N}}$$

The same calculation was used to calculate indel mutation rate, with *u_bs_* replaced with *u_id_*.

### Data availability

Illumina DNA sequences for the MA lines used in this study are deposited under the following Bioprojects: *A. tumefaciens* PRJNA256312, *B. subtilis* PRJNA256312, *M. florum* PRJNA256337, *S. epidermidis* PRJNA256338, and *V. cholerae* PRJNA256339.

File S1 contains detailed descriptions of eukaryotic *u_id_* estimates, as well as calculations for *G_e_*, *G_nc_*, θ_s_, π_s_, and phylogenetic independent contrasts for both eukaryotic and prokaryotic organisms. Figure S1 contains average depth of sequencing coverage for each MA line in *A. tumefaciens*, *S. epidermidis*, and *V. cholerae*. Figure S2 displays the similarity in θ_s_ when increasing the number of unique alleles analyzed. Figure S3 shows the frequency distribution of mutant calls across MA lines. Table S1 contains the calculation for the estimated limit of selection to fix antimutators. Figure S4, Figure S5, Figure S6, and Table S2 contain statistical support for the DBH. Dataset S1, Dataset S2, Dataset S3, and Dataset S4 contain single nucleotide polymorphisms and indels for prokaryotic and eukaryotic organisms generated in this study.

## Results

To examine the effect of genetic drift on mutation-rate evolution, it is necessary to derive accurate estimates of the mutation rate and genetic diversity across phylogenetically diverse organisms. WGS has greatly improved our ability to estimate such parameters. Highly accurate measurements of *u_bs_* and *u_id_* can be obtained through WGS of MA lines, in which repeated single-organism bottlenecking minimizes the efficiency of selection, allowing for the accumulation of all but the most deleterious mutations (Lynch *et al.* 2008; Denver *et al.* 2009; Ossowski *et al.* 2010; Sung *et al.* 2012a, 2012b, 2015; Schrider *et al.* 2013).

The power of genetic drift is related to the inverse of the effective population size [1/*N_e_* for haploids, 1/(2*N_e_*) for diploids]. Under the assumption of neutrality, the effective population size (*N_e_*) can be estimated from the average nucleotide heterozygosity at silent sites in natural populations (π_s_), or as a function of the number of segregating sites in the population (θ_s_), both of which lead to expected values equal to 4*N_e_u_bs_* in diploids and 2*N_e_u_bs_* in haploids (Kimura 1983). For most organisms analyzed in this study, enough WGS data were available to allow calculation of species-specific θ_s_ values (see File S1 and Table 1). For the remaining species, we pooled large available multilocus-sequence studies to estimate π_s_. In all cases, we set the estimates of θ_s_ or π_s_ equal to 4*N_e_u_bs_* in diploids (2*N_e_u_bs_* in haploids), and solved for *N*_e_ by factoring out *u_bs_*. Because this calculation only involves *u_bs_*, the estimate of *N_e_* is uninfluenced by sampling error in *u_id_*, thus providing an independent trait measurement by which to test the DBH (see File S1 for further evaluation of the nonindependence issue).

**Table 1:** Effective genome size (*G_e_*), indel events per site per generation (*u_id_*), base-substitution mutation rate per generation (*u_bs_*), θ_s_ (or π_s_, denoted by *) measurements for population mutation rate (Watterson 1975; Tajima 1989; Fu 1995), and estimated effective population size (*N_e_*) for seven prokaryotic and eight eukaryotic organisms (see File S1 for details)

Species	Label	*G_e_* (× 10^7^ Sites)	*G_c_* + *G_nc_* (× 10^7^ Sites)	*u_id_* (× 10^−10^ per Site per Generation)	*u_bs_* (× 10^−10^ Events per Site per Generation)	θ_s_ or π_s_	*N_e_* (× 10^6^)
Prokaryotes							
* Agrobacterium tumefaciens*	*Agt*	0.50	0.57	0.30	2.92	0.200*	342.47
* Bacillus subtilis*	*Bs*	0.36	0.43	1.20*^d^*	3.35*^d^*	0.041	61.19
* Escherichia coli*	*Ec*	0.39	0.46	0.37*^e^*	2.00*^e^*	0.071	179.60
* Mesoplasma florum*	*Mf*	0.07	0.08	23.10*^f^*	97.80*^f^*	0.021	1.07
* Pseudomonas aeruginosa*	*Pa*	0.59	0.67	0.14*^g^*	0.79*^g^*	0.033*	210.70
* Staphlyococcus epidermidis*	*Se*	0.21	0.26	1.13	7.40	0.052	35.14
* Vibrio cholerae*	*Vc*	0.34	0.39	0.18	1.15	0.110	478.26
Eukaryotes							
* Arabidopsis thaliana*	*At*	4.21	5.55*^a^*	11.20*^h^*	69.50*^h^**^,^**^p^*	0.008	0.29
* Caenorhabditis elegans*	*Ce*	2.50	6.37*^b^*	6.69*^i^*	14.50*^q^*	0.003	0.54
* Chlamydomonas reinhardtii*	*Cr*	3.92	5.51	0.44*^j^*	3.80*^j^*	0.032	43.31
* Drosophila melanogaster*	*Dm*	2.32	8.86*^c^*	4.61*^k^*	51.65*^k^*	0.018	0.86
* Homo sapiens*	*Hs*	3.65	21.75*^b^*	18.20*^l^*	135.13*^l^*	0.001	0.02
* Mus musculus*	*Mm*	3.55	27.17*^b^*	3.10*^m^*	54.00*^m^*	0.004*	1.77
* Paramecium tetraurelia*	*Pt*	5.68	7.28	0.04*^n^*	0.19*^n^*	0.008	101.80
* Saccharomyces cerevisiae*	*Sc*	0.87	1.02*^b^*	0.92*^o^*	2.63*^o^*	0.004	7.78

*G_c_* + *G_nc_* is the effective genome size when including the total amount of coding (*G_c_*) and noncoding DNA (*G_nc_*) that is estimated to be under purifying selection. Footnotes in *u_id_* and *u_bs_* indicate data sources (rates pooled when multiple data sources are available), and, when absent, indicate data generated in this study (see *Materials and Methods*).

a Haudry *et al.* (2013).

b Siepel *et al.* (2005).

c Halligan *et al* .(2004).

d Sung *et al.* (2015).

e Lee *et al.* (2012).

f Sung *et al.* (2012a).

g Sung *et al.* (2012b).

h Ossowski *et al.* (2010).

i Lipinski *et al.* (2011).

j Sung *et al.* (2012a); Ness *et al.* (2015).

k Schrider *et al.* (2013).

l Conrad *et al.* (2011); O’Roak *et al.* (2011, 2012); Kong *et al.* (2012); Campbell and Eichler (2013); Wang and Zhu (2014); The 1000 Genomes Project Consortium (2015).

m Uchimura *et al.* (2015).

n Sung *et al.* (2012b).

o Lynch *et al.* 2008); (Zhu *et al.* 2014).

p Ossowski *et al.* (2010); (Yang *et al.* 2015).

q Lipinski *et al.* (2011).

To provide additional data for testing whether the power of genetic drift constrains the lower limit of indel mutation-rate evolution, we performed MA experiments in *A. tumefaciens* str. C58, *S. epidermidis* ATCC 12228, and *V. cholerae* 2740-80. Each bacterial MA experiment was initiated from multiple lines derived from a single progenitor colony, each of which was repeatedly bottlenecked to accumulate mutations for an average of 5819, 7170, and 6453 generations, respectively (see *Materials and Methods*; harmonic mean population sizes between transfers were 13.4 (0.1), 12.6 (0.3), and 14.9 (0.2), respectively). Then, 101-bp paired-end WGS was applied to randomly selected MA lines (47 *A. tumefaciens*, 22 *S. epidermidis*, and 46 *V. cholerae* MA lines, Dataset S1). The average sequencing coverage depth is greater than 20 × per site across all MA lines surveyed in these organisms (Figure S1), and greater than 50 × per site for 93.75% (150/160) of the MA lines, providing high accuracy for measurement of *u_bs_* and *u_id_*. Mutations were called and categorized for each of the three species (Dataset S3 and Dataset S4), with *u_bs_* and *u_id_* shown in Table 1.

To test the DBH, we combined *u_bs_* and *u_id_* from the three bacterial species analyzed in this study with *u_bs_* and *u_id_* from four bacterial and eight eukaryotic MA WGS studies (Table 1, Dataset S1, Dataset S2, Dataset S3, and Dataset S4), and also included the same estimates for human derived from WGS of parent-offspring trios. *u_id_* includes all indel events in each of the 15 study species (see File S1). Due to the highly repetitive DNA sequence in eukaryotic genomes, the number of large indels events (> 9 bp) in eukaryotes may be downwardly biased when using WGS methods. Therefore, our estimate of the number of large indel events also includes events identified by comparative genome hybridization arrays for organisms where data were available (Lynch *et al.* 2008; Lipinski *et al.* 2011). Large indel events only account for 15.0% of total indels events across the study bacteria (76/506, Dataset S4), suggesting that any underestimation of the number of large indel events should only have a small effect on *u_id_*.

**Figure 1:**
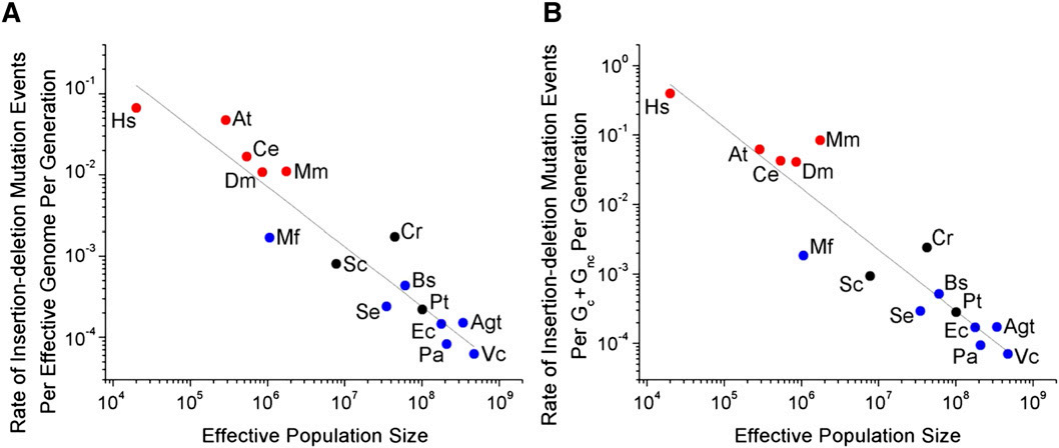
Relationship between the rate of indel events per generation per effective genome (*u_id_G_e_*) and effective population size (*N_e_*). (A) Regression: log_10_(*u_id_G_e_*) = 2.23(0.48) – 0.73(0.07)log_10_*N_e_* (*r*^2^ = 0.89, *P* = 6.81 × 10^−8^, d.f. = 13), with SE of parameter estimates shown in parentheses. Blue circles represent bacteria, red circles multicellular eukaryotes, and black circles unicellular eukaryotes, with all data summarized in Table 1. The full list of indel events for analyzed organisms is presented in Dataset S4. Chromosomal distributions of indel events at each site across all mutation-accumulation experiments are shown in Figure S1, A and B. (B) Relationship when adding the number of estimated noncoding sites under purifying selection into the effective genome size (*G_c_* + *G_nc_*) for eukaryotic organisms. Regression: log_10_[*u*id(*G_c_* + *G_nc_*)] = 3.49(0.66) – 0.87(0.09)log_10_*N_e_* (*r*^2^ = 0.87, *P* = 3.13 × 10^−7^, d.f. = 13).

**Figure 2:**
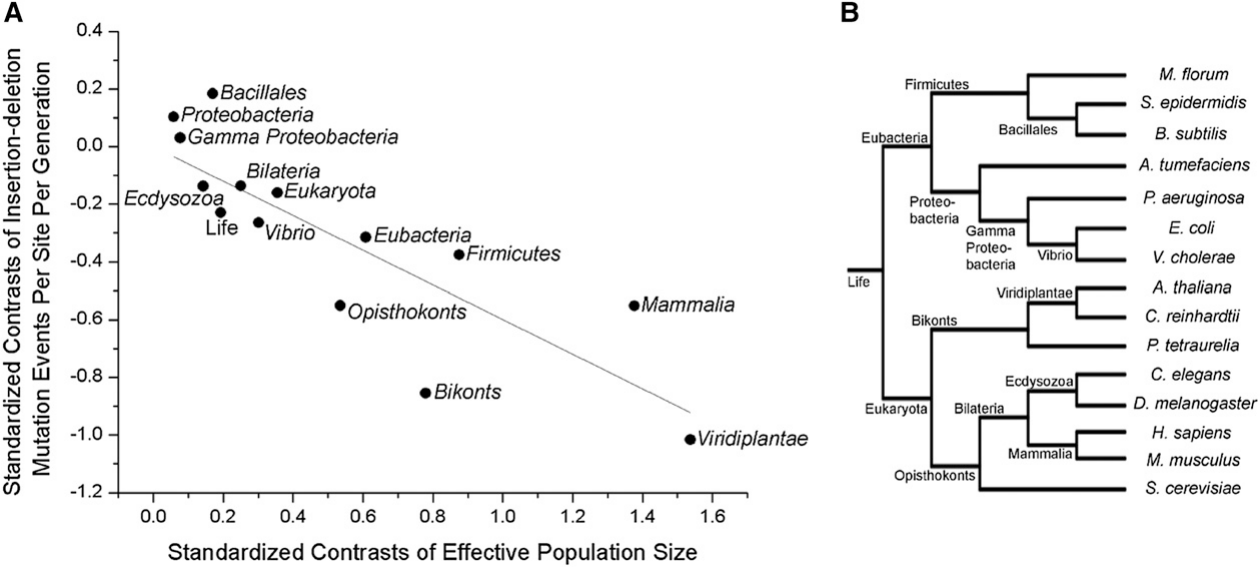
Relationship between indel events per site per generation (*u_id_G_e_*) and effective population size (*N_e_*) after phylogenetic correction. (A) Standardized phylogenetically independent contrasts performed using Compare (Martins 2004), and the PDAP module in Mesquite (Garland *et al.* 1993), with branch lengths of 1.0. The regression equation of the contrasts through the origin is: *u_id_G_e_* = –0.60(0.07)*N_e_* (*r*^2^ = 0.83, *P* = 1.28 × 10^−6^, d.f. = 13), with SE in parentheses. (B) Phylogenetic tree showing the relationship between organisms.

To determine the genome-wide deleterious burden in each organism associated with indel mutations, we multiplied *u_id_* with *G_e_*, approximating the latter by the proteome size of that organism. A plot of the logs of the two parameters of *u_id_G_e_* and *N*_e_ against one another yields a strong negative correlation across all of cellular life (Figure 1A, *r*^2^ = 0.89). Because the power of genetic drift is inversely proportional to *N_e_*, this observation is consistent with the idea that selection operates to reduce mutation rates to a barrier imposed by random genetic drift. Phylogenetic nonindependence may complicate observed relationships between genomic attributes and *N*_e_ (Whitney and Garland 2010). However, the relationship between *N*_e_ and *u_id_G_e_* remains robust even after phylogenetic correction (Figure 2, A and B, *r*^2^ = 0.83), indicating that the correlation between *N*_e_ and *u_id_G_e_* reflects a true biological phenomenon across the Tree of Life.

## Discussion

Because the DBH makes general predictions about the pattern of molecular and cellular evolution across the Tree of Life, because our focus is on one of the central determining factors in the evolutionary process (the mutation rate), and because the patterns appear so strong, it is essential to consider the range of factors that might give rise to the observed statistical relationships, and also to alternative evolutionary hypotheses for them. We first consider three issues with respect to estimating the key parameters *N_e_*, *u_bs_*, *u_id_*, and *G_e_*, and then elaborate on the significance and implications of the relationship between *u_id_G_e_* and *N*_e_ for our understanding of molecular evolution.

First, we address the estimation of *N_e_*, one of the most difficult issues in empirical population genetics. Because populations fluctuate in density over time, any estimate of *N_e_* must reflect a long-term average, presumably approximating a harmonic mean, not the immediate population state. Because evolution is a long-term process, however, the mean is most relevant to the issues being examined herein. Recent selective sweeps or population bottlenecks can transiently modify levels of genetic variation at individual loci (Charlesworth 2009; Karasov *et al.* 2010), introducing noise into any estimates of *N_e_* derived from limited numbers of genetic loci, but this would reduce the strength of any true underlying correlation between the rate of mutation (*u_id_G_e_*), and long-term *N*_e_, *i.e.*, would operate against our ability to detect the expected signal of the DBH.

Such effects are especially likely in asexual species, where the possibility of reduced recombination might subject many neutral nucleotide sites to the effects of selection on nearby, linked sites. Thus, to minimize sampling error, wherever possible, we have relied upon genome-wide sampling of the number of segregating sites to obtain a low-variance estimator of *N*_e_*u* from observations on silent sites (Watterson 1975). The utilization of an average θ_s_ across a large number of nucleotide sites and individual isolates reduces the effects of evolutionary sampling variance associated with chromosomally localized and population-specific sweeps arising within individual species (Fu and Li 1993). Using available genomic data, we calculated θ_s_ across a large number of within-species genotypic isolates, excluding nearly identical lab strains that originated from the same individual (see *Materials and Methods*). Although no estimates of silent-site diversity (the source of *N*_e_ estimates) are without error, estimates derived from segregating polymorphic sites across large-scale genomic data sets appear quite robust (Figure S2). Moreover, should the levels of variation sampled in our various study species reflect recent events, to which mutation-rate evolution has not had adequate time to respond (Brandvain and Wright 2016), this would only introduce noise into the relationship between effective population size and mutation rates.

Second, as we have noted earlier, there is some concern that correlations between estimates of mutation rates and *N_e_* could, in part, be spurious artifacts resulting from the use of estimates of *N_e_* obtained by dividing measures of standing variation at silent-sites by *u_bs_* (Sung *et al.* 2012a). If the sampling variance of *u_bs_* is substantial enough, this could lead to a negative correlation between the observed *u_bs_* and extrapolated *N_e_* estimates, and, if there were a sampling covariance between *u_bs_* and *u_id_*, this could carry over into the current study. In the Supplemental Material (File S1, Figure S4, Figure S5, Figure S6 and Figure S7), we provide complementary analyses to that in Sung *et al.* (2012a), indicating that the sampling variance of *u_bs_* from WGS-MA studies is not large enough to explain the negative correlation previously seen between *u_bs_* and *N_e_* estimates. Because *u_bs_* and *u_id_* are measured by different methods, the sampling covariance between these two measures is expected to be negligible. We emphasize that it is the sampling variance, not the evolutionary variance, that is of concern here. The variance of the log-scaled values of *u_bs_* would have to exceed the log-scaled values of *N_e_* by ∼two orders of magnitude in order to create the negative correlations that we observe (File S1). As an extreme way of looking at the situation, if silent-site variation were constant across all taxa, and the parametric values of mutation rates and *N_e_* were obtained without error, the only explanation for the data would be a true underlying negative evolutionary covariance between the two features. In fact, there is a marginal negative correlation between estimates of π_s_ and *u_bs_* (Figure S3, Figure S4, Figure S5, Figure S6, Figure S7, and Table S2), further bolstering the idea that *u_bs_* and *u_id_* decline evolutionarily as *N_e_* increases.

Third, the DBH proposes that the strength of selection operating to reduce the indel mutation rate is based upon the total indel deleterious mutational load, *i.e.*, the product of the mutational rate of appearance of indels at individual nucleotide sites (*u_id_*), and the number of sites under selective constraint in the genome (*G_e_*, approximated by the proteome size of the organism). However, some noncoding DNA (*e.g.*, noncoding functional RNAs, and *cis*-regulatory units in untranslated regions or introns) is certainly under selective constraint, with mutations at these sites increasing the deleterious mutational load. Thus, it can be argued that the estimated number of nucleotides affecting fitness (*G_e_*) scales differently than the protein-coding region of the genome, particularly in larger eukaryotic genomes with a considerable number of noncoding sites (Halligan *et al.* 2004; Siepel *et al.* 2005; Halligan and Keightley 2006). Difficulties can arise when estimating the proportion of noncoding DNA that is under selective constraint (*G_nc_*), as the estimated number of such sites can vary greatly depending on the model used to define noncoding DNA, and the identification of conserved noncoding DNA is highly sensitive to the available phylogeny (Siepel *et al.* 2005). Nevertheless, if we sum the estimated total amount of noncoding DNA under selective constraint (*G_nc_*, see File S1) with that of coding DNA (*G_c_*), we find that *u_id_*(*G_c_* + *G_nc_*) and *N_e_* remain highly correlated (Figure 1B, *r*^2^ = 0.87), simply because the fraction of functional noncoding DNA increases with the total amount of coding DNA.

We currently adhere to the DBH as an explanation for the phylogenetic pattern of mutation-rate variation primarily because it has been difficult to reconcile the patterns with alternative hypotheses. In the introduction, we provided arguments as to why selection for replication speed appears to be unlikely to explain a negative correlation between mutation rates and population size in unicellular species, and, in multicellular species, the simultaneous deployment of hundreds to thousands of origins of replication makes such an explanation even more unlikely. Nor does a general constraint on replication fidelity explain the data.

**Figure 3:**
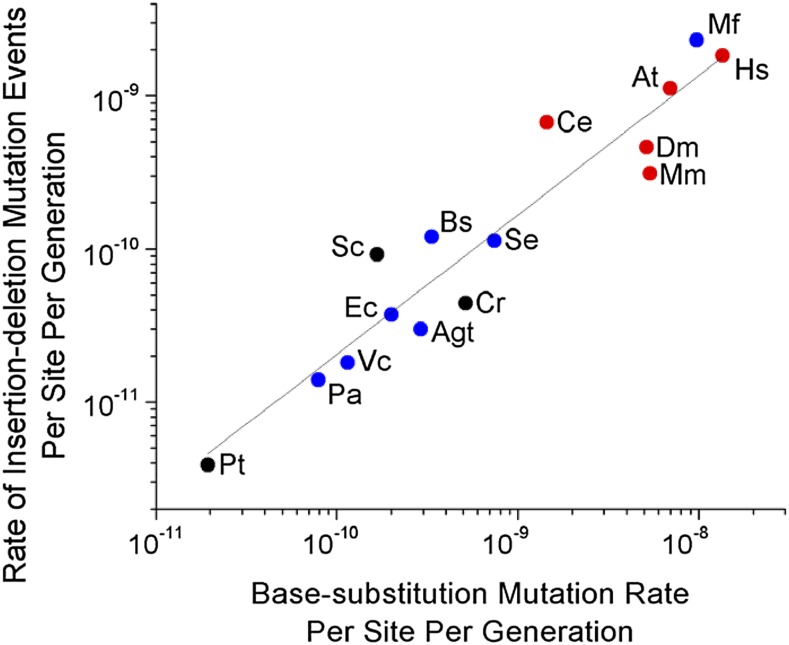
Relationship between the rate of indel events per site per generation (*u_id_*), and the base-substitution mutation rate per site per generation (*u_bs_*). Regression: log_10_(*u_id_*) = –1.56(0.74) + 0.91(0.08) log_10_*u_bs_* (*r*^2^ = 0.90, *P* = 4.13 × 10^−8^, d.f. = 13). SE measurements are shown in parentheses. Blue circles represent eubacteria, red circles multicellular eukaryotes, and black circles unicellular eukaryotes, with all data summarized in Table 1.

A second potential explanation for variation in the per-generation mutation rate is that it is driven largely by variation in numbers of germline cell divisions (Ness *et al.* 2012), but this cannot be reconciled with the fact that the base-substitution mutation rate scales negatively with *N_e_* in analyses entirely restricted to unicellular species (Sung *et al.* 2012a). In all such species, there is one cell division per generation, and yet the base-substitution mutation rate per site per cell division ranges from ∼10^−11^ in *Paramecium tetraurelia* (Sung *et al.* 2012b) to ∼10^−8^ in *M. florum* (Sung *et al.* 2012a). Similarly, the number of indel mutational events per site per cell division differs by over two orders of magnitude across unicellular organisms (Table 1 and Figure 3), and the negative regression with *N_e_* remains significant when confined to unicellular species (Figure 1, *r*^2^ = 0.66, *P* = 0.003).

A third hypothesis for mutation-rate evolution is that selection is effective enough to reduce the error rate to the point at which the physical laws of thermodynamics take over (Kimura 1967). However, it is difficult to reconcile this argument with the data now showing that mutation rates vary by three orders of magnitude, as there are no known mechanisms by which basic biophysical features (such as diffusion coefficients and stochastic molecular motion) would vary by this degree among the cytoplasms of different taxa. There is, of course, the issue of evolved differences in the biochemical features and efficiency of operation of the proteins involved in replication and repair. However, this type of variation is in the explanatory domain of the DBH. The DBH postulates that replication fidelity is typically not at the maximum possible level of refinement, but just the lowest level possible under the prevailing level of random genetic drift, which varies substantially among lineages.

That a decline in replication fidelity should decline with decreasing effective population size appears to be a unique prediction of the DBH. Although other theoretical work has been done on mutation-rate evolution, in no case is this type of scaling obviously predicted (acknowledging that this has not been a central focus of such work). For example, allowing for a role of beneficial mutations, Kimura (1967) and Leigh (1970) suggested that the long-term rate of adaptation is maximized when the genome-wide mutation rate equals the rate of population fixation of beneficial mutations. The precise predictions of this hypothesis are not entirely clear, but because mutations arise at a higher rate in large populations, and, if beneficial, fix with higher probabilities, a positive association between the mutation rate and *N_e_* seems to be implied. A rather different model argues that populations should evolve genome-wide mutation rates equal to the average effect of a deleterious mutation (Orr 2000; Johnson and Barton 2002), which seems to imply an optimal mutation rate independent of population size (unless one wishes to postulate an association between average mutational effect and *N_e_*, for which we are unaware of any evidence).

The DBH proposes that new alleles that reduce the genome-wide indel mutation rate (*i.e.*, anti-mutators) can be promoted by selection only if they provide a significant enough advantage to offset the power of genetic drift. The average selective effect of an antimutator or mutator allele (which operate opposite to each other) can be approximated by *st·*∆*U_id_*, with ∆*U_id_* representing the change in the genome-wide indel mutation rate with respect to the population mean rate, *s* being the average reduction in fitness per mutation (Lynch 2010), and *t* being the number of generations a mutation remains associated with its mutator genetic background (Lynch 2011). ∆*U_id_* can be approximated by the change in the indel mutation rate over the effective genome, or ∆*u_id_G_e_* (Lynch 2011). By setting *st*∆*u_id_G_e_* equal to the power of random genetic drift [1/*N_e_* for haploids, 1/(2*N_e_*) for diploids], we can acquire some sense of the average reduction in the indel mutation rate that is required for the power of selection to exceed power of genetic drift. Using estimates of an average value of the selective coefficient (*s* = 0.01) (Lynch *et al.* 1999; Eyre-Walker and Keightley 2007), and assuming that free recombination unlinks mutation-rate modifier alleles from their background every ∼2 generations in sexually outcrossing species (*t* = 2) (Lynch 2010), solving *st*∆*u_id_G_e_* = 1/*N_e_* [= 1/(2*N_e_*) for diploids] for ∆*u_id_* suggests that the average antimutator must reduce the indel mutation rate by greater than ∼0.1–1% in most organisms (Table S1) in order to be promoted by selection. One major limitation of this kind of analysis is that values of *s* and *t* are not well known, and are likely vary across organisms. A second and equally important caveat is that the prior analysis assumes that mutator and antimutator alleles arise with equal frequency. Owing to the high level of refinement of the replication and repair machinery, it seems much more likely that mutations involving the components of such machinery will increase rather than decrease the mutation rate. This will push the equilibrium mutation rate to higher levels than expected (Lynch 2008), although without quantitative information on such bias, it is difficult to determine the exact position at which the mutation rate will stall.

Finally, we note that because recombination unlinks alleles from their genetic background, the capacity of selection to enhance replication fidelity is ultimately a function of the recombination rate (Kimura 1967; Lynch 2008). Thus, it may be viewed as surprising that bacteria, which do not undergo meiotic recombination, exhibit a relationship between *u_id_* and *N*_e_ similar to that in eukaryotic species engaging in periodic to regular meiosis (Figure 1, A and B). It should be noted, however, that bacterial recombination occurs through multiple mechanisms (transformation, conjugation, and/or transduction). Many bacterial species are known to naturally undergo high rates of recombination, with ratios of recombination to mutation rates frequently being comparable to those in multicellular eukaryotes (Feil and Spratt 2001; Lynch 2007; Doroghazi *et al.* 2014; Lassalle *et al.* 2015), so, in this sense, comparable behavior of bacterial and eukaryotic species is not unexpected.

In summary, as in our previous work on the base-substitution mutation rate (Sung *et al.* 2012a), the strong correlation between the genome-wide indel rate and *N_e_* appears not to be a statistical artifact. Moreover, among various hypotheses that have been suggested for mutation-rate evolution, the DBH appears to provide the most compatible explanation for the ∼1000-fold range of variation of this trait across the Tree of Life. As noted above, the molecular mechanisms that generate and resolve base-substitution and indel mutations differ in a number of ways, and the rate of occurrence of these two types of mutations differ by one to two orders of magnitude (with *u_id_* ranging from 1.8 to 11.9% of *u_bs_*, presumably because of the elevated deleterious effects of indel mutations). Yet, despite these differences, both *u_bs_* and *u_id_* scale similarly with changes in *N_e_* (Figure 3, *r*^2^ = 0.89). Because the forces of mutation, selection, and drift apply to all biological traits, the maximum achievable level of refinement for other fundamental cellular traits may also be influenced by the drift barrier.

## Supplementary Material

Supplemental Material
