# Supplementary material for: Evolution of the Insertion-Deletion Mutation Rate Across the Tree of Life
Source: G3 (Bethesda). 2016 Jun 15;6(8):2583–91. doi: 10.1534/g3.116.030890 (PMC4978911; doi:10.1534/g3.116.030890)
Supplement: Supplemental Material [file supp_6_8_2583__index.html]

Evolution of the Insertion-Deletion Mutation Rate Across the Tree of Life — Supplemental Material 

# Evolution of the Insertion-Deletion Mutation Rate Across the Tree of Life

## Supplemental Material for Sung, *et al*, 2016

**Files in this Data Supplement:**

- Figure S1 - Average depth of coverage in *Staphylococcus epidermidis*, *Agrobacterium tumefaciens*, and *Vibrio cholerae*, mutation-accumulation experiments. (.pdf, 73 KB)
- Figure S2 - Estimate of Θs from minor allele counts (*Escherichia coli* data shown). (.pdf, 75 KB)
- Figure S3 - A) Distribution of the read frequency for insertion-deletion mutation calls using BWA mapping. B) Bimodal distribution displaying the proportion of MA lines sharing the same (size and motif) putative insertion-deletion mutation call using BWA mapping for same organisms. (.pdf, 84 KB)
- Figure S4 - The r2 value of the regression between *Ne* and *Uid* under the null hypothesis, as a function of the root mean square error (RMSE) of estimates of *ubs*. (.pdf, 92 KB)
- Figure S5 - The r2 value of the regression between *Ne* and *Uid* under the null hypothesis, as a function of the correlation between errors in estimate of *ubs* and *uid*. (.pdf, 93 KB)
- Figure S6 - A scatter-plot of effective genome size (*Ge*) against silent site diversity (π*s*). (.pdf, 99 KB)
- Figure S7 - A scatter-plot of base-substitution mutation rate (*ubs*) against silent site diversity (π*s*). (.pdf, 97 KB)
- File S1 - Supplemental Materials and Methods. (.pdf, 184 KB)
- Table S1 - Limit of selection to fix antimutators that reduce *uid* in each species. (.pdf, 106 KB)
- Table S2 - Regression coefficients of the variables relating effective population size to deleterious mutation rate. (.pdf, 71 KB)
- Dataset S1 - Base-substitution and insertion-deletion (indel) mutation summary statistics for the prokaryotes *Agrobacterium tumefaciens* (Agt), *Bacillus subtilis* (Bs), *Escherichia coli* (Ec), *Mesoplasma florum* (Mf), and *Vibrio cholerae* (Vc). (.pdf, 265 KB)
- Dataset S2 - *Caenorhabditis elegans* N2 insertion-deletion (indel) summary statistics after an average of 250 generations (Gen). (.pdf, 39 KB)
- Dataset S3 - List of base-substitution mutation calls for *Agrobacterium tumefaciens (Agt)*, *Bacillus subtilis* (Bs), *Escherichia coli* (Ec), *Mesoplasma florum* (Mf), and *Vibrio cholerae* (Vc). (.pdf, 693 KB)
- Dataset S4 - List of insertion-deletion mutation calls for *Agrobacterium tumefaciens* (Agt), *Bacillus subtilis* (Bs), *Escherichia coli* (Ec), *Mesoplasma florum* (Mf), *Vibrio cholerae* (Vc), and *Caenorhabditis elegans* (Ce). (.pdf, 230 KB)
