# Supplementary material for: Evolution of the Insertion-Deletion Mutation Rate Across the Tree of Life
Source: G3 (Bethesda). 2016 Jun 15;6(8):2583–91. doi: 10.1534/g3.116.030890 (PMC4978911; doi:10.1534/g3.116.030890)
Supplement: Supplemental Material [file supp_g3.116.030890_FigureS1.pdf]

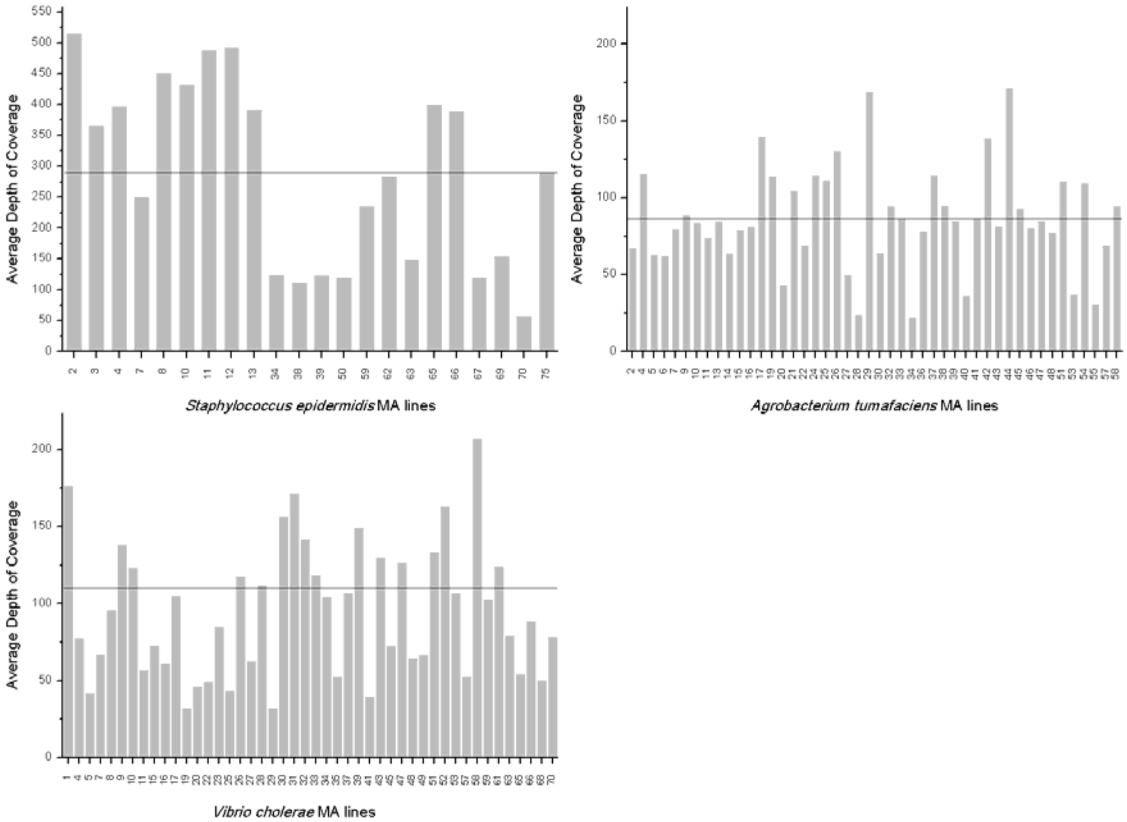

Figure S1. Average depth of coverage in *Staphylococcus epidermidis*, *Agrobacterium tumefaciens*, and *Vibrio cholerae*, mutation-accumulation experiments. MA line number for each experiment is listed below each vertical bar. Depth of coverage is determined by the total number of nucleotides aligned by the BWA alignment algorithm divided by the size of the genome. Grey horizontal line represents the average depth of coverage across all MA lines for each species prior to filtering (see Mutation Calling).
