# Supplementary material for: Evolution of the Insertion-Deletion Mutation Rate Across the Tree of Life
Source: G3 (Bethesda). 2016 Jun 15;6(8):2583–91. doi: 10.1534/g3.116.030890 (PMC4978911; doi:10.1534/g3.116.030890)
Supplement: Supplemental Material [file supp_g3.116.030890_FigureS2.pdf]

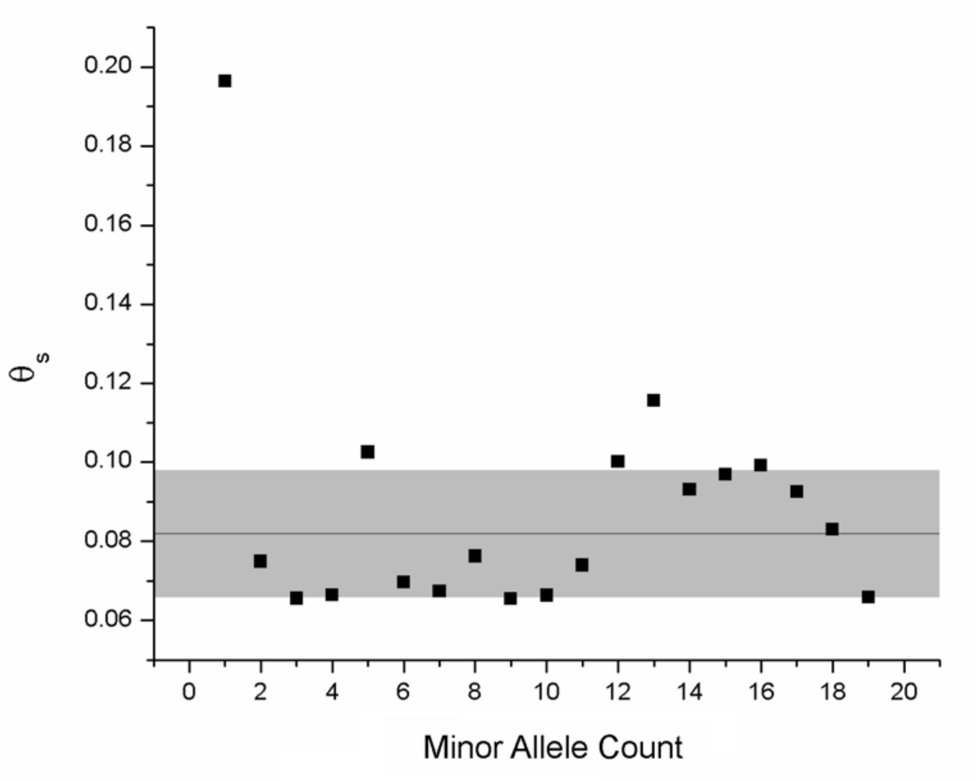

Figure S2. Estimate of θ_s from minor allele counts (*Escherichia coli* data shown). *X*-axis denotes the minor allele count (observed number of strains that have the allele). Formula used to calculate θ_s is derived from equations 6-8 of Fu (1995). Horizontal gray line indicates the average of θ_s across all allele frequencies excluding unique alleles (minor allele count of 1) and is closely related to Watterson's theta (See Supplemental Materials – Calculation of θ_s). Shaded region indicates one standard deviation from the mean of all points except for unique alleles.
