# Supplementary material for: Evolution of the Insertion-Deletion Mutation Rate Across the Tree of Life
Source: G3 (Bethesda). 2016 Jun 15;6(8):2583–91. doi: 10.1534/g3.116.030890 (PMC4978911; doi:10.1534/g3.116.030890)
Supplement: Supplemental Material [file supp_g3.116.030890_FigureS3.pdf]

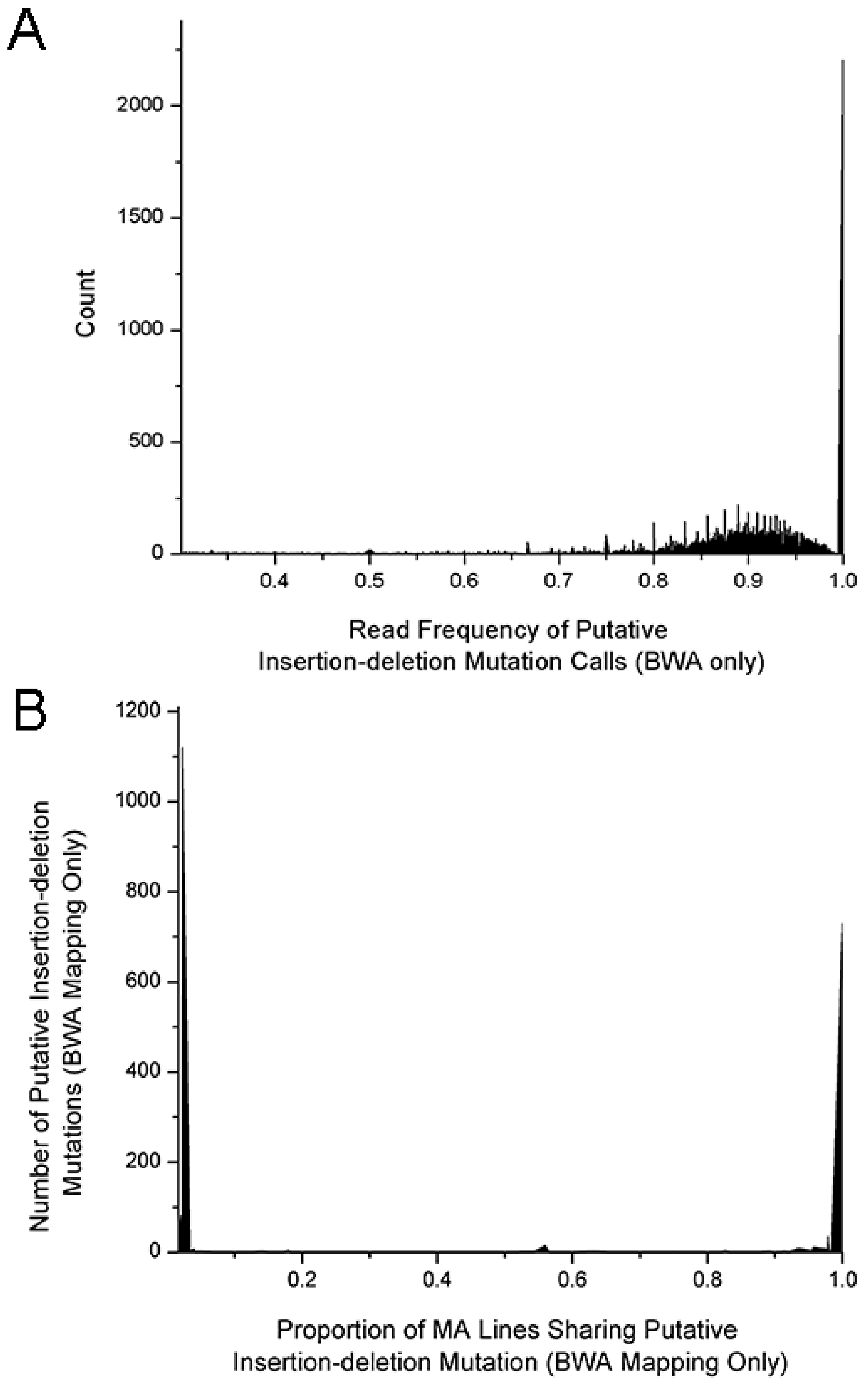

A
Count
2000
1500
1000
500
0
0.4
0.5
0.6
0.7
0.8
0.9
1.0
Read Frequency of Putative
Insertion-deletion Mutation Calls (BWA only)
B
Number of Putative Insertion-deletion
Mutations (BWA Mapping Only)
1200
1000
800
600
400
200
0
0.2
0.4
0.6
0.8
1.0
Proportion of MA Lines Sharing Putative
Insertion-deletion Mutation (BWA Mapping Only)

Figure S3. **A)** Distribution of the read frequency for insertion-deletion mutation calls using BWA mapping. Read frequency is determined by the number of reads representing the putative insertion-deletion mutation call at a site, divided by the total number of reads spanning that site. The total number includes counts from *Agrobacterium tumefaciens*, *Bacillus subtilis*, *Escherichia coli*, *Mesoplasma florum*, *Staphylococcus epidermidis*, and *Vibrio cholerae* MA experiments prior to filtering (see Mutation Calling). **B)** Bimodal distribution displaying the proportion of MA lines sharing the same (size and motif) putative insertion-deletion mutation call using BWA mapping for same organisms.
