# Supplementary material for: Evolution of the Insertion-Deletion Mutation Rate Across the Tree of Life
Source: G3 (Bethesda). 2016 Jun 15;6(8):2583–91. doi: 10.1534/g3.116.030890 (PMC4978911; doi:10.1534/g3.116.030890)
Supplement: Supplemental Material [file supp_g3.116.030890_FigureS5.pdf]

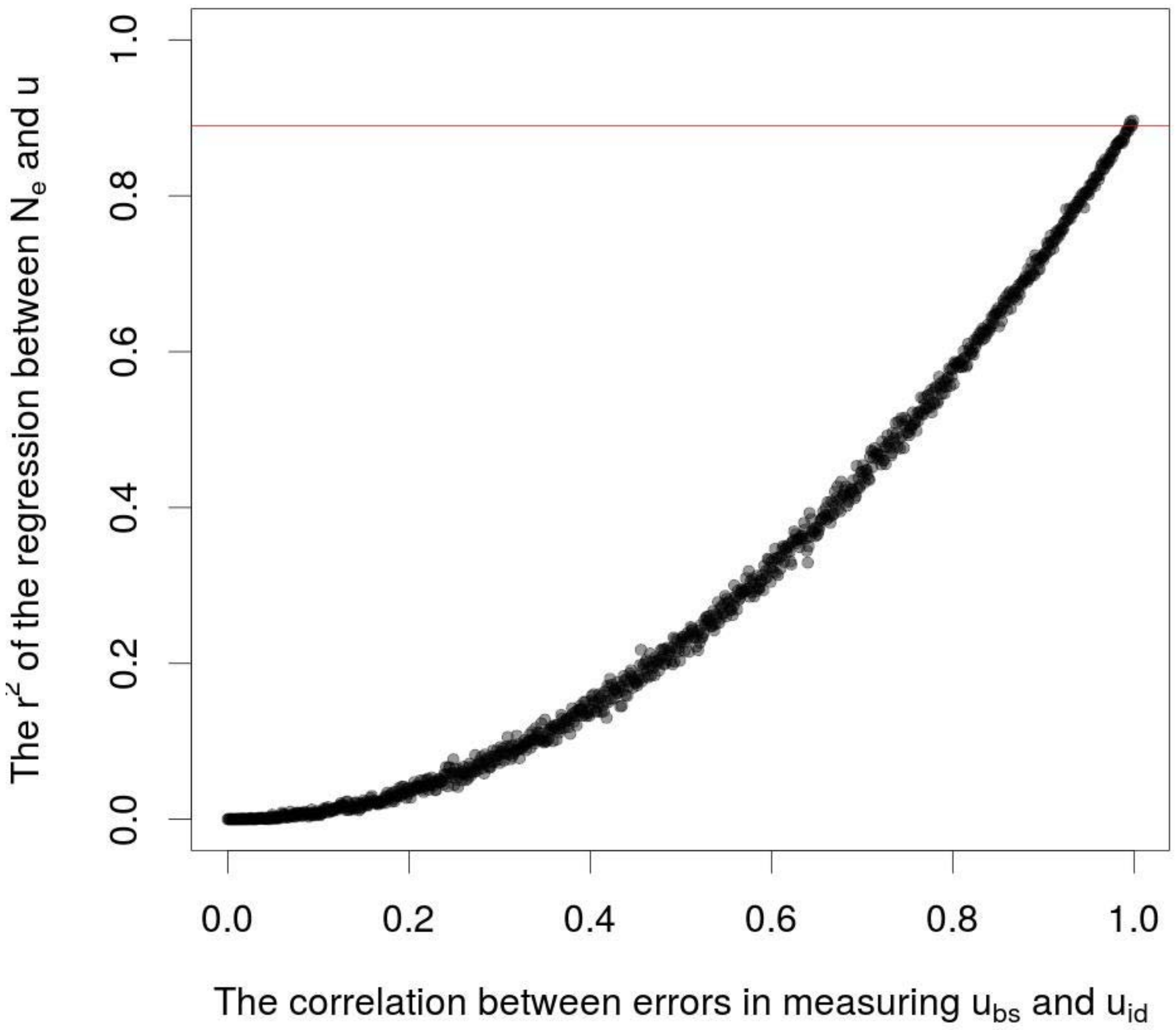

Figure S5: The r^2 value of the regression between N_e and U_{id} under the null hypothesis, as a function of the correlation between errors in estimate of u_{bs} and u_{id}. The errors are assumed to be log normally distributed (see main text). The horizontal red line is drawn at t value of 0.89, the r^2 of the observed data.
