# Supplementary figures and images for: Evolution of the Insertion-Deletion Mutation Rate Across the Tree of Life
Source: G3 (Bethesda). 2016 Jun 15;6(8):2583–91. doi: 10.1534/g3.116.030890 (PMC4978911; doi:10.1534/g3.116.030890)

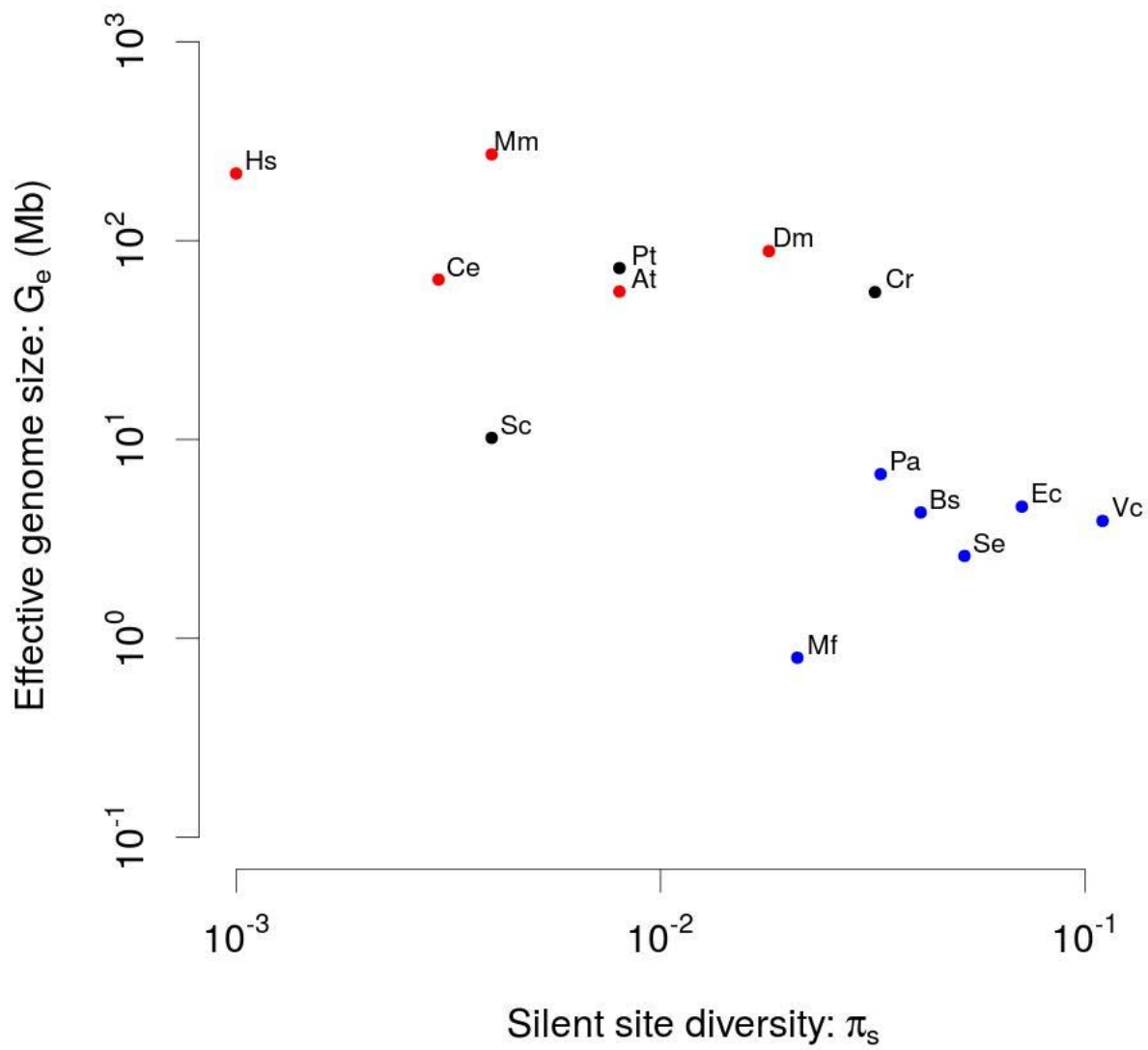

Figure S6: A scatter-plot of effective genome size ( $G_e$ ) against silent site diversity ( $\pi_s$ ).

Supplement: Supplemental Material [file supp_g3.116.030890_FigureS6.pdf]
