# Supplementary material for: Evolution of the Insertion-Deletion Mutation Rate Across the Tree of Life
Source: G3 (Bethesda). 2016 Jun 15;6(8):2583–91. doi: 10.1534/g3.116.030890 (PMC4978911; doi:10.1534/g3.116.030890)
Supplement: Supplemental Material [file supp_g3.116.030890_FileS1.pdf]

Supplemental Material and Methods

Eukaryotic u_{id} estimates. For eukaryotes, base-substitution mutation rates were taken from the original mutation-accumulation experiment or study. The indel mutation rates were calculated as follows:

Arabidopsis thaliana: Indel mutation rates were taken at face value from the *Arabidopsis thaliana* MA experiment (OSSOWSKI *et al.* 2010). The study was able to verify 8 short deletions, 5 short insertions, and 4 long deletions, for a total of 17 events, yielding an indel mutation rate of 1.12×10^{-9} events per site per generation.

Caenorhabditis elegans: The small-event indel mutation rate for *C. elegans* was not determined in the original mutation accumulation study (DENVER *et al.* 2009), so we reanalyzed the data using the methods described above. The small-event indel mutation rate was found to be 5.74×10^{-10} events per site per generation. The original study did not use paired-end sequencing, so the PINDEL analysis was excluded for *Caenorhabditis elegans* (DENVER *et al.* 2009). Instead, we relied on comparative genome-hybridization analysis performed by Lipinski *et al.* (LIPINSKI *et al.* 2011) to determine the rate of large-scale indel events. In this study, the authors found 30 large-scale insertions and 11 large-scale deletions of gene regions over 432 generations, yielding a rate of 9.50×10^{-11} events per site per generation. The joint indel mutation rate is then 6.69×10^{-10} events per site per generation.

Chlamydomonas reinhardtii and *Paramecium tetraurelia*: The indel event mutation rate was taken from prior mutation-accumulation studies (SUNG *et al.* 2012a; SUNG *et al.* 2012b).

Saccharomyces cerevisiae: Indel-event mutation rates were estimated using a joint average of indel events from two *Saccharomyces cerevisiae* mutation accumulation studies (LYNCH *et al.* 2008; ZHU *et al.* 2014). A genome-wide mutation study in *S. cerevisiae* identified 26 small-scale indel events across 145 lines over 2062 generations and 12.2 Mb, yielding a small-indel event rate of 7.13×10^{-12} events per site per generation (ZHU *et al.* 2014). However, the study did not provide a measurement of large-scale indel events. Using pulse-field gel electrophoresis and comparative genome hybridization (aCGH), an earlier mutation-accumulation study identified the rate of large insertions to be 0.00055 events per line per cell division, and a rate of large deletions to be 0.00047 events per line per cell division. Dividing those rates by the total genome size of *S. cerevisiae* (12.2 Mb) yields a rate of 3.85×10^{-11} and 4.51×10^{-11} events per site per generation respectively. The total of the three mutation classes from the two studies yields a combined rate of 9.16×10^{-11} events per site per generation.

Drosophila melanogaster: The rate of indel mutation events was taken from the most recent mutation-accumulation study in *Drosophila melanogaster* that incorporated paired-end sequencing information (SCHRIDER *et al.* 2013). The authors estimated the small-scale indel mutation rate to be ~2.43×10^{-10} events per site per generation. The study also verified 22 large deletions and 7 large duplications yielding a large-scale indel mutation rate of 2.18×10^{-10} events per site per generation. The joint total of the two mutation types is then 4.61×10^{-10} events per site per generation.

Mus musculus: The rate of indel mutation events was taken from the most recent mutation-accumulation study in *Mus musculus* (UCHIMURA et al. 2015).

Homo sapiens: Small indel mutation rates are approximately 15% of base-substitution mutation rates, with estimates at disease alleles in human trios approximately 10% of base-substitution mutation rates (0.20×10^{-9} insertion events per site per generation and 0.58×10^{-9} events per site per generation), and whole-genome sequencing data estimating a ~20% of the ratio of base-substitution mutation events to indel mutation events (LACHANCE *et al.* 2012). We took the average rate of small indel mutation events to be 15% of the base-substitution mutation rate: $1.16\times10^{-8} \times 0.15 = 1.74\times10^{-9}$. In addition to small indels, mobile element insertions (~0.05×10^{-9} events per site per generation), and large CNVs (~0.03×10^{-9} events per site per generation), summarized by Campbell and Eichler yield a joint estimate of ~1.82×10^{-9} indel events per site per generation (CAMPBELL AND EICHLER 2013).

Calculation of G_e. The effective genome size (G_e) for *Arabidopsis thaliana* (ARABIDOPSIS GENOME 2000) and *Chlamydomonas reinhardtii (MERCHANT et al. 2007)* were determined by the total exon size (bp) from the published genome papers. G_e for the remaining eukaryotes were determined by the coding DNA sequence (CDS) from the newest available reference genome as follows: *Caenorhabditis elegans* - Wormbase release WS242 (HARRIS *et al.* 2014), *Drosophila melanogaster* - Flybase release FB2014_03 (ST PIERRE *et al.* 2014), *Homo sapiens* - Ensembl Release 75, ~1.5% of total size (FLICEK *et al.* 2014), *Mus musculus* – assembly GRCm38.p4, *Paramecium tetraurelia* - ParameciumDB release v1.92 (ARNAIZ AND SPERLING 2011), and *Saccharomyces cerevisiae* - Saccharomyces Genome Database S288C release R64-1-1 (ENGEL *et al.* 2014).

For prokaryotes, G_e was taken from the CDS of the reference genome for the organism used in the mutation-accumulation study. The CDS for prokaryotes were downloaded from the National Center for Biotechnology Information FTP site.

Calculation of G_{nc}. To calculate the effective genome size when considering non-coding sites that are under purifying selection (G_{nc}) in eukaryotes, we determined the proportion of non-coding sites that are under purifying selection when compared to coding sites (G_c), and increased the total number of sites by that proportion. Although the estimate of non-coding sites under selection varies from source to source, we used the following sources for estimates of G_{nc} : *Homo sapiens* and *Mus musculus* (SIEPEL *et al.* 2005), *D. melanogaster* (HALLIGAN *et al.* 2004; SIEPEL *et al.* 2005), *C. elegans* (SIEPEL *et al.* 2005), *A. thaliana* genome (HAUDRY *et al.* 2013), *S. cerevisiae* (SIEPEL *et al.* 2005). We lacked any data for *P. tetraurelia* and *C. reinhardtii*, so we weighted *P. tetraurelia* and *C. reinhardtii* using the closest relative from our phylogenetic mirror tree (*A. thaliana* - Fig 2B). For prokaryotes, G_{nc} includes all non-coding sites.

Calculation of θ_s or π_s. For the eukaryotes *Arabidopsis thaliana* (NORDBORG *et al.* 2005; SCHMID *et al.* 2005), *Caenorhabditis elegans* (CUTTER 2006), *Chlamydomonas reinhardtii* (FLOWERS *et al.* 2015; NESS *et al.* 2015), *Drosophila melanogaster* (LYNCH 2010), *Homo sapiens* (Lynch 2006), *Mus musculus* (LYNCH 2010), *Paramecium tetraurelia* (Catania *et al.* 2009), and *Saccharomyces cerevisiae* (SCHACHERER *et al.* 2009), θ_s or π_s estimates were taken

directly from genome-wide studies.

For five bacterial species, θ_s was derived from comparative analysis of the genome-wide sequencing projects for that species that are publicly available for download at the National Center for Biotechnology Information (NCBI). Within a species, genome projects that differed from another genome project by less than 1/100th of Illumina sequencing error (less than 500 differences in a 5Mbp genome) were assumed to be nearly identical lab strains, and only one of the strains was used in the comparative analysis. For each bacterium, the following genomes were used to calculate θ_s (redundant genomes not listed):

Bacillus subtilis: BAB1, BSn5, BSP1, natto BEST195, NCIB 3610, RO NN 1, SC 8, XF 1, and inaquosorum KCTC 13429.

Escherichia coli: B REL606, SE15, SE11, KO11FL, UMN026, ABU 83972, O157 H7 EC4115, K 12 substr MG1655, DH1, O55 H7 CB9615, 536, APEC O1, clone D i14, CFT073, ATCC 8739, Xuzhou21, UM146, 42, IAI1, IAI39, O111 H 11128, UMNK88, O103 H2 12009, O83 H1 NRG 857C, 55989, S88, E24377A, O26 H11 11368, HS, SMS 3 5, O7 K1 CE10, O127 H6 E2348 69, IHE3034, O55 H7 RM12579, ETEC H10407, ED1a, O157 H7 EDL933, P12b, and NA114.

Mesoplasma florum: L1, and W37.

Staphylococcus epidermidis: NIHLM039, AU12 03, NIHLM020, VCU144, VCU117, NIHLM067, NIHLM001, NIHLM003, NIHLM031, FRI909, ATCC 12228, NIH04008, W23144, NIHLM049, M23864 W2 grey, VCU081, VCU129,VCU065, VCU118, VCU128, VCU071, NIHLM053, NIHLM018, NIHLM040, VCU120, BCM HMP0060, NIHLM061, RP62A, NIH051475, SK135, VCU123, NIHLM015, VCU041, VCU105, NIHLM088, BVS058A4, NIHLM021, M23864 W1, NIHLM008, NIHLM087, VCU125, NIH05005, NIHLM023, VCU127, and NIHLM037.

Vibrio cholera: Ex25, IEC224, LMA3984 4, M66 2, MJ 1236, O1 2010EL 1786, O1 biovar El Tor N16961, O395 uid159869, O395 uid58425, and EJY3.

For each species, reciprocal tblastx was performed for all combination of genes across the different genomes. From the comparisons, Orthomcl (Li *et al.* 2003) was used to determine orthologous gene families. Gene families that contained co-orthologs or in-paralogs were discarded. Alleles that are observed only once across the orthologs are generally elevated in sequence diversity and likely reflect sequencing errors (Fig. S2). Therefore, these orthologs were discarded from all analyses except in *Mesoplasma florum*, where only two genomes were available. The following formula was used to calculate θ_s from the remaining orthologous gene families (Fu 1995):

$$\frac{2}{(n-1)\times L}\sum_{i=2}^{n/2}\left(\frac{S_i \times (1+\delta_{i,(n-i)})}{\frac{1}{i}+\frac{1}{n-1}}\right)$$

where n is the total number of genomes, S_i is the number of sites segregating at frequency i/n, and L is the total number of sites analyzed. If $i = n_i$, then $\delta_{i,(n-i)} = 1$, otherwise $\delta_{i,(n-i)} = 0$.

For *A. tumefaciens*, π_s was derived from prior multi-locus sequencing typing experiments as follows: A total of 109 alleles were surveyed across *acsA-I , glyA, lysA-I, pgi, rpoD,* and *thrB* genes in the circular chromosome, and *aosA-II, ftsZ* and *lysA-II* genes in the linear chromosome (Marri *et al.* 2008). The weighted average of π_s across all 9 loci is 0.200. Estimates of π_s estimates for *Pseudomonas aeruginosa* were taken directly from a prior study (Lynch 2010).

Phylogenetic Independent Contrasts. Phylogenetic Independent Contrast (PIC) is a statistical method to determine whether cross-taxon relationship between traits remain statistically significant after accounting for phylogenetic topology (Felsenstein 1985). We used the two programs Compare (Martins 2004) and Mesquite v2.75 (Maddison and Maddison 2011) to calculate the PIC for the traits involved in insertion-deletion mutation rates and effective population size. A composite species tree was constructed for both programs using phylogenies previously reported for prokaryotes and eukaryotes (Wu and Eisen 2008; Parfrey *et al.* 2010). In Compare an d in Mesquite using the PDAP module (Garland *et al.* 1993), phylogenetic-generalized least squares were calculated, with all branch length set to 1.0 (Fig. 3A). In Mesquite, Grafen's, Nee's and Pagel's methods also all yielded significant results ($P<1\times10^{-5}$) (Maddison and Maddison 2011). Mirror tree displayed in Fig. 2B.

Statistical support for the drift-barrier hypothesis. This section examines the statistical support for the drift-barrier hypothesis (DBH) of mutation rate evolution presented in both Sung *et al.*, 2012, which examined the base pair mutation rate (u_{bs}), and in this manuscript, which examines the indel mutation rate (u_{id}). The DBH proposes that selection operates to reduce the per generation deleterious mutation rate (U), and effective population size (N_e - a measure of the power of random genetic drift) determines the minimum benefit that selection can effectively favor. Thus, under the DBH, it is expected that the deleterious mutation rate is proportional to the inverse of effective population size (U□N_e^{-1}). Neither of the variables N_e or U can be measured directly. Effective population size is calculated from the ratio of silent site diversity (π_s) and the per base pair substitution mutation rate ($N_e=\pi_s/u_{bs}$), and the deleterious mutation rate is calculated from the product of effective genome size (G_e) and the per base pair substitution mutation rate ($U_{bs}=G_e\times u_{bs}$) or the per base pair indel mutation rate ($U_{id}=G_e\times u_{id}$). Assuming that the DBH is correct, then U and N_e for both mutation types becomes linear after a log transformation, $\log_{10}U \approx k\text{-}\log_{10}N_e$, where k is some constant of proportionality. However, it is necessary to ensure that there is no statistical relationship between the parameters that would drive an erroneous relationship. We show that without introducing unrealistic amount of sampling error, we are unable to reproduce the relationship observed in this manuscript when the parameters N_e and U are uncorrelated. The python script used to generate the figures presented are available from https://github.com/LynchLab/DBH_SIMULATIONS.

If there is no correlation between U and N_e then the covariance between the two terms is $Cov(\log_{10}U, \log_{10}N_e)$. We can rewrite U and N_e in terms of the variables we measure, giving us:

$$Cov(log_{10}U, log_{10}N_e) = Cov(log_{10}G_e + log_{10}u_{bs}, log_{10}\pi_s - log_{10}u_{bs}),$$
(S1)

which expands to

$$Cov(log_{10}\pi_s, log_{10}u_{bs}) - Cov(log_{10}G_e, log_{10}u_{bs}) + Cov(log_{10}\pi_s, log_{10}u_{bs}) - Var(log_{10}u_{bs}).$$ (S2)

Because $Var(\log_{10}u_{bs})$ is strictly positive, for equation S2 to evaluate to 0 some of the covariance terms must be non-zero. If there is no relationship between effective population size and the per base pair mutation rate, *i.e.* $Cov(\log_{10}N_e, \log_{10}u_{bs})=0$, then by expanding this term in a similar manner as before we see that $Cov(\log_{10}\pi_s, \log_{10}u_{bs})=Var(\log_{10}u_{bs})$. When this relationship is satisfied no other covariance terms are needed, and we can assume that effective genome size is uncorrelated with silent site diversity, $Cov(\log_{10}G_e, \log_{10}\pi_s)=0$, and the per base pair mutation rate, $Cov(\log_{10}G_e, \log_{10}u_{bs})=0$. Under these assumptions there will be a correlation between the per base pair mutation rate and both per-genome deleterious mutation rates and silent-site diversity. Both of these correlations are consistent with biological expectations. Silent-site diversity measures the ratio of the power of drift and the power of mutation, and if effective population size remains constant as mutation rates increase, then there will be a corresponding increase in silent site diversity. Similarly, if the per base pair mutation rate has no effect on effective genome size, then an increased per base pair mutation rate should lead to an increased deleterious mutation rate. In summary, the hypothesis that U and N_e are uncorrelated is fundamentally equivalent to the claims that the slope of the regression between $\log_{10}\pi_s$ and $\log_{10}u_{bs}$ is 1, and that effective genome size is uncorrelated with silent-site diversity and per base pair substitution rates.

The null hypothesis is rejected when $Cov(\pi_s, u_{bs}) \neq Var(u_{bs})$. There is nothing intrinsically wrong with this approach, however, we do have to be wary of possibility that measurement error has decreased the expected covariance of π_s and u_{bs} beneath the true value. This problem can be dealt with by assessing the magnitude of measurement error necessary to diminish the covariance of π_s and u_{bs} to the extent observed.

In the fifteen organism in this study a weak negative relationship is observed between u_{bs} and π_s (β=-0.35, r^2=0.21). In order to observe this result erroneously, the magnitude of measurement error would need to exceed biological variability by nearly **two orders of magnitude** (Fig. S4). Additionally, to create a spurious relationship between indel mutation rates and effective population size the errors in estimating base-substitution and indel mutation rates would have to be highly correlated (Fig. S5). A correlation may exist under some circumstances because the correct estimation of both rates depends on a high-quality reference genome and sufficient sequencing depth to distinguish between sequencing error and true mutations. However, the fifteen organisms used in this study are model organisms with high-quality reference genomes, and the MA lines are sequenced to high depths, so sampling error ie likely to be well below the two orders of magnitude required to create a spurious relationship.

If the DBH is correct, we should see a relationship between N_e and U. This directly implies a negative correlation between G_e and π_s. The drift-barrier hypothesis makes no direct prediction of a relationship between u_{bs} and π_s. N_e might have some effect on effective genome size, which would create an indirect relationship between N_e and π_s of similar magnitude and opposite sign. Additionally, N_e could be systematically underestimated because many silent sites are weakly constrained. Although this would generate a negative relationship between u_{bs} and π_s, no corresponding relationship would be generated between G_e and π_s, since it would

reflect an error in our estimation of N_e, and not an evolutionary relationship between these variables.

Finally, one of the predictions of the DBH is that effective genome size and silent-site diversity should be correlated. This prediction is appealing in that it should be truly independent of the mutation rate. Measurement errors in mutation rates, no matter how profound, could not effect this signal (Fig. S6 and Table S2). The correlation of G_e and π_s, and the anti-correlation of u_{bs} and π_s are inconsistent with the null hypothesis. Thus the correlation of N_e with both U_{bs} and U_{id} is statistically and biologically significant, and evolutionary hypotheses which explain these relationships must be sought.

Arabidopsis Genome, I., 2000 Analysis of the genome sequence of the flowering plant *Arabidopsis thaliana*. Nature 408**:** 796-815.

Arnaiz, O., and L. Sperling, 2011 ParameciumDB in 2011: new tools and new data for functional and comparative genomics of the model ciliate *Paramecium tetraurelia*. Nucleic Acids Res 39**:** D632-636.

Campbell, C. D., and E. E. Eichler, 2013 Properties and rates of germline mutations in humans. Trends Genet 29**:** 575-584.

Catania, F., F. Wurmser, A. A. Potekhin, E. Przybos and M. Lynch, 2009 Genetic diversity in the *Paramecium aurelia* species complex. Mol Biol Evol 26**:** 421-431.

Cutter, A. D., 2006 Nucleotide polymorphism and linkage disequilibrium in wild populations of the partial selfer *Caenorhabditis elegans*. Genetics 172**:** 171-184.

Denver, D. R., P. C. Dolan, L. J. Wilhelm, W. Sung, J. I. Lucas-Lledo *et al.*, 2009 A genome-wide view of *Caenorhabditis elegans* base-substitution mutation processes. Proc Natl Acad Sci U S A 106**:** 16310-16314.

Engel, S. R., F. S. Dietrich, D. G. Fisk, G. Binkley, R. Balakrishnan *et al.*, 2014 The reference genome sequence of *Saccharomyces cerevisiae*: then and now. G3 (Bethesda) 4**:** 389-398.

Felsenstein, J., 1985 Phylogenies and the Comparative Method. American Naturalist 125**:** 1-15.

Flicek, P., M. R. Amode, D. Barrell, K. Beal, K. Billis *et al.*, 2014 Ensembl 2014. Nucleic Acids Res 42**:** D749-755.

Flowers, J. M., K. M. Hazzouri, G. M. Pham, U. Rosas, T. Bahmani *et al.*, 2015 Whole-Genome Resequencing Reveals Extensive Natural Variation in the Model Green Alga *Chlamydomonas reinhardtii*. Plant Cell 27**:** 2353-2369.

Fu, Y. X., 1995 Statistical properties of segregating sites. Theor Popul Biol 48**:** 172-197.

Garland, T., A. W. Dickerman, C. M. Janis and J. A. Jones, 1993 Phylogenetic Analysis of Covariance by Computer-Simulation. Systematic Biology 42**:** 265-292.

Halligan, D. L., A. Eyre-Walker, P. Andolfatto and P. D. Keightley, 2004 Patterns of evolutionary constraints in intronic and intergenic DNA of *Drosophila*. Genome Res 14**:** 273-279.

Harris, T. W., J. Baran, T. Bieri, A. Cabunoc, J. Chan *et al.*, 2014 WormBase 2014: new views

of curated biology. Nucleic Acids Res 42: D789-793.

Haudry, A., A. E. Platts, E. Vello, D. R. Hoen, M. Leclercq *et al.*, 2013 An atlas of over 90,000 conserved noncoding sequences provides insight into crucifer regulatory regions. Nat Genet 45: 891-898.

Lachance, J., B. Vernot, C. C. Elbers, B. Ferwerda, A. Froment *et al.*, 2012 Evolutionary history and adaptation from high-coverage whole-genome sequences of diverse African hunter-gatherers. Cell 150: 457-469.

Li, L., C. J. Stoeckert, Jr. and D. S. Roos, 2003 OrthoMCL: identification of ortholog groups for eukaryotic genomes. Genome Res 13: 2178-2189.

Lipinski, K. J., J. C. Farslow, K. A. Fitzpatrick, M. Lynch, V. Katju *et al.*, 2011 High spontaneous rate of gene duplication in *Caenorhabditis elegans*. Curr Biol 21: 306-310.

Lynch, M., 2006 The origins of eukaryotic gene structure. Mol Biol Evol 23: 450-468.

Lynch, M., 2010 Evolution of the mutation rate. Trends Genet 26: 345-352.

Lynch, M., W. Sung, K. Morris, N. Coffey, C. R. Landry *et al.*, 2008 A genome-wide view of the spectrum of spontaneous mutations in yeast. Proc Natl Acad Sci USA 105: 9272-9277.

Maddison, W. P., and D. R. Maddison, 2011 Mesquite: a modular system for evolutionary analysis. Version 2.75 http://mesquiteproject.org.

Marri, P. R., L. K. Harris, K. Houmiel, S. C. Slater and H. Ochman, 2008 The effect of chromosome geometry on genetic diversity. Genetics 179: 511-516.

Martins, E. P., 2004 Compare, version 4.6b. Computer programs for the statistical analysis of comparative data. Distributed by the author at http://compare.bio.indiana.edu. Department of Biology, Indiana University, Bloomington, IN.

Merchant, S. S., S. E. Prochnik, O. Vallon, E. H. Harris, S. J. Karpowicz *et al.*, 2007 The *Chlamydomonas* genome reveals the evolution of key animal and plant functions. Science 318: 245-250.

Ness, R. W., A. D. Morgan, R. B. Vasanthakrishnan, N. Colegrave and P. D. Keightley, 2015 Extensive de novo mutation rate variation between individuals and across the genome of *Chlamydomonas reinhardtii*. Genome Res 25: 1739-1749.

Nordborg, M., T. T. Hu, Y. Ishino, J. Jhaveri, C. Toomajian *et al.*, 2005 The pattern of polymorphism in *Arabidopsis thaliana*. PLoS Biol 3: e196.

Ossowski, S., K. Schneeberger, J. I. Lucas-Lledo, N. Warthmann, R. M. Clark *et al.*, 2010 The rate and molecular spectrum of spontaneous mutations in *Arabidopsis thaliana*. Science 327: 92-94.

Parfrey, L. W., J. Grant, Y. I. Tekle, E. Lasek-Nesselquist, H. G. Morrison *et al.*, 2010 Broadly sampled multigene analyses yield a well-resolved eukaryotic tree of life. Syst Biol 59: 518-533.

Schacherer, J., J. A. Shapiro, D. M. Ruderfer and L. Kruglyak, 2009 Comprehensive polymorphism survey elucidates population structure of *Saccharomyces cerevisiae*. Nature 458: 342-345.

Schmid, K. J., S. Ramos-Onsins, H. Ringys-Beckstein, B. Weisshaar and T. Mitchell-Olds,

2005 A multilocus sequence survey in *Arabidopsis thaliana* reveals a genome-wide departure from a neutral model of DNA sequence polymorphism. Genetics 169**:** 1601-1615.

Schrider, D. R., D. Houle, M. Lynch and M. W. Hahn, 2013 Rates and genomic consequences of spontaneous mutational events in *Drosophila melanogaster*. Genetics 194**:** 937-954.

Siepel, A., G. Bejerano, J. S. Pedersen, A. S. Hinrichs, M. Hou *et al.*, 2005 Evolutionarily conserved elements in vertebrate, insect, worm, and yeast genomes. Genome Res 15**:** 1034-1050.

St Pierre, S. E., L. Ponting, R. Stefancsik, P. McQuilton and C. FlyBase, 2014 FlyBase 102--advanced approaches to interrogating FlyBase. Nucleic Acids Res 42**:** D780-788.

Sung, W., M. S. Ackerman, S. F. Miller, T. G. Doak and M. Lynch, 2012a Drift-barrier hypothesis and mutation-rate evolution. Proc Natl Acad Sci U S A 109**:** 18488-18492.

Sung, W., A. E. Tucker, T. G. Doak, E. Choi, W. K. Thomas *et al.*, 2012b Extraordinary genome stability in the ciliate *Paramecium tetraurelia*. Proc Natl Acad Sci U S A 109**:** 19339-19344.

Uchimura, A., M. Higuchi, Y. Minakuchi, M. Ohno, A. Toyoda *et al.*, 2015 Germline mutation rates and the long-term phenotypic effects of mutation accumulation in wild-type laboratory mice and mutator mice. Genome Res 25**:** 1125-1134.

Wu, M., and J. A. Eisen, 2008 A simple, fast, and accurate method of phylogenomic inference. Genome Biol 9**:** R151.

Zhu, Y. O., M. L. Siegal, D. W. Hall and D. A. Petrov, 2014 Precise estimates of mutation rate and spectrum in yeast. Proc Natl Acad Sci U S A.
