# Supplementary material for: Evolution of the Insertion-Deletion Mutation Rate Across the Tree of Life
Source: G3 (Bethesda). 2016 Jun 15;6(8):2583–91. doi: 10.1534/g3.116.030890 (PMC4978911; doi:10.1534/g3.116.030890)
Supplement: Supplemental Material [file supp_g3.116.030890_TableS1.pdf]

Table S1.
Limit of selection to fix antimutators that reduce u_{id} in each species. Average effect of an antimutator can be approximated by $st\Delta u_{id}(G_c+G_{nc})$, with s being the average reduction in fitness per mutation, $\Delta u_{id}(G_c+G_{nc})$ being the reduction in indel mutation rate (Δu_{id}) multiplied by the total size of deleterious targets (G_c+G_{nc}), and t being the time over which the antimutator is associated with the genetic background. $\%\Delta u_{id}$ indicates the percentage reduction of the current indel mutation rate that must be exceeded for selection to promote fixation of the antimutator, i.e., the point where the strength of selection promoting the antimutator is equal to the power of random genetic drift: $st\Delta u_{id}(G_c+G_{nc}) = 1/N_e$ ($1/2N_e$ in diploids).

Organism	G_c+G_{nc} ($\times 10^7$ sites)	N_e ($\times 10^6$)	u_{id} ($\times 10^{-10}$ events /site/gen.)	s	t	Δu_{id} (events per site per gen.)	$\%\Delta u_{id}$
Prokaryotes							
Agrobacterium tumefaciens	0.57	341.52	0.30	0.01	2.00	2.57×10^{-14}	0.09%
Bacillus subtilis	0.43	61.19	1.20	0.01	2.00	1.90×10^{-13}	0.16%
Escherichia coli	0.46	179.60	0.37	0.01	2.00	6.05×10^{-14}	0.16%
Mesoplasma florum	0.08	1.070	23.10	0.01	2.00	5.84×10^{-11}	2.53%
Pseudomonas auriginosa	0.67	210.70	0.14	0.01	2.00	3.54×10^{-14}	0.25%
Staphylococcus epidermidis	0.26	35.14	1.13	0.01	2.00	5.47×10^{-13}	0.48%
Vibrio cholera	0.39	478.26	0.18	0.01	2.00	2.68×10^{-14}	0.15%
Eukaryotes							
Arabidopsis thaliana	5.55	0.29	11.20	0.01	2.00	1.55×10^{-12}	0.14%
Caenorhabditis elegans	6.37	0.54	6.69	0.01	2.00	7.27×10^{-13}	0.11%
Chlamydomonas reinhardtii	5.51	43.31	0.44	0.01	2.00	1.05×10^{-14}	0.02%
Drosophila melanogaster	8.86	0.86	4.61	0.01	2.00	3.28×10^{-13}	0.07%
Homo sapiens	21.75	0.02	18.20	0.01	2.00	5.75×10^{-12}	0.32%
Mus musculus	27.17	1.77	3.10	0.01	2.00	5.20×10^{-14}	0.02%
Paramecium tetraurelia	7.28	101.80	0.04	0.01	2.00	3.37×10^{-15}	0.09%
Saccharomyces cerevisiae	1.02	7.78	0.92	0.01	2.00	3.15×10^{-13}	0.34%
