# Supplementary material for: Evolution of the Insertion-Deletion Mutation Rate Across the Tree of Life
Source: G3 (Bethesda). 2016 Jun 15;6(8):2583–91. doi: 10.1534/g3.116.030890 (PMC4978911; doi:10.1534/g3.116.030890)
Supplement: Supplemental Material [file supp_g3.116.030890_TableS2.pdf]

Table S2: Regression coefficients of the variables relating effective population size to deleterious mutation rate. Slopes are predicted under the null model (where effective population size is unassociated with the deleterious mutation rate) and the minimal version of the drift-barrier hypothesis (where only the deleterious mutation rate is affected by effective population size).

Covariance terms	Slope	regression slope β (lower CI, upper CI)	Null	DBH
G_e, u_{bs}	0.07	(-0.4, 0.4)	0	0
G_e, π_s	-0.84	(-1.4, -0.3)	0**	-1
u_{bs}, π_s	-0.62	(-1.3, -0.)	1***	0*
