# Supplementary material for: Evolution of the Insertion-Deletion Mutation Rate Across the Tree of Life
Source: G3 (Bethesda). 2016 Jun 15;6(8):2583–91. doi: 10.1534/g3.116.030890 (PMC4978911; doi:10.1534/g3.116.030890)
Supplement: Supplemental Material [file supp_g3.116.030890_DatasetS1.pdf]

Dataset S1.

Base-substitution and insertion-deletion (indel) mutation summary statistics for the prokaryotes *Agrobacterium tumefaciens* (Agt), *Bacillus subtilis* (Bs), *Escherichia coli* (Ec), *Mesoplasma florum* (Mf), and *Vibrio cholerae* (Vc). SEMis the standard error. Ts/Tv is the transition/transversion ratio.

MA Line	Substitutions						Indels		Ts/Tv	Sites ($\times 10^{6}$)	Gen.	Base-sub Rate ($\times 10^{-10}$) /site/gen.	Indel Rate ($\times 10^{-10}$) /events /gen.
	Transitions		Transversions										
	AT>GC	GC>AT	AT>TA	GC>TA	AT>CG	GC>CG	Ins.	Del.					
Agt|2	2	6	1	0	2	3	0	1	1.33	5.45	5842	4.40	0.31
Agt|4	2	5	1	0	0	0	0	1	7.00	5.55	5816	2.48	0.31
Agt|5	1	5	0	1	1	1	0	3	2.00	5.66	5816	2.73	0.91
Agt|6	0	6	1	0	1	3	0	0	1.20	3.84	5842	4.90	0.00
Agt|7	1	1	1	2	2	3	1	2	0.25	5.66	5842	3.02	0.91
Agt|9	2	5	0	2	0	2	0	1	1.75	5.65	5869	3.31	0.30
Agt|10	3	8	0	3	1	4	0	1	1.38	5.45	5816	5.99	0.32
Agt|11	1	4	0	3	0	0	1	0	1.67	5.66	5842	2.42	0.30
Agt|13	4	3	0	2	1	2	0	0	1.40	5.64	5789	3.68	0.00
Agt|14	2	3	0	2	2	2	1	1	0.83	5.65	5735	3.39	0.62
Agt|15	1	3	0	2	2	0	0	0	1.00	5.66	5842	2.42	0.00
Agt|16	2	2	0	1	3	1	0	0	0.80	5.66	5816	2.73	0.00
Agt|17	2	4	0	4	0	0	0	0	1.50	5.56	5842	3.08	0.00
Agt|19	0	3	0	1	0	1	0	0	1.50	3.79	5816	2.27	0.00
Agt|20	3	3	1	0	0	0	1	0	6.00	5.66	5869	2.11	0.30
Agt|21	0	4	0	0	1	0	0	0	4.00	5.55	5869	1.53	0.00
Agt|22	0	2	0	3	0	1	0	0	0.50	5.66	5842	1.81	0.00
Agt|24	3	2	1	0	1	1	0	0	1.67	5.57	5789	2.48	0.00
Agt|25	1	3	1	0	0	2	1	1	1.33	5.64	5816	2.13	0.61
Agt|26	0	3	0	1	1	1	1	2	1.00	5.66	5789	1.83	0.91
Agt|27	0	0	3	1	0	2	0	0	0.00	5.66	5842	1.81	0.00
Agt|28	1	5	0	1	1	4	0	1	1.00	5.65	5842	3.64	0.30
Agt|29	4	4	1	3	0	0	1	0	2.00	5.57	5762	3.74	0.31
Agt|30	4	4	1	3	0	0	1	1	2.00	5.66	5842	3.63	0.60
Agt|32	1	8	2	1	1	0	1	0	2.25	5.55	5816	4.03	0.31
Agt|33	4	6	0	3	0	1	0	0	2.50	5.45	5869	4.38	0.00
Agt|34	1	4	0	0	0	1	1	0	5.00	3.77	5869	2.71	0.45
Agt|36	1	4	1	1	1	0	1	2	1.67	5.66	5869	2.41	0.90
Agt|37	1	2	0	2	0	0	0	0	1.50	5.66	5735	1.54	0.00
Agt|38	0	5	0	0	1	4	0	0	1.00	5.66	5735	3.08	0.00
Agt|39	1	3	0	1	0	2	1	0	1.33	5.66	5816	2.12	0.30
Agt|40	6	3	0	1	3	1	1	0	1.80	5.66	5842	4.23	0.30
Agt|41	1	1	1	3	0	1	0	0	0.40	5.63	5816	2.14	0.00
Agt|42	2	3	3	2	2	3	0	2	0.50	5.67	5869	4.51	0.60
Agt|43	3	4	1	2	0	3	0	0	1.17	5.66	5842	3.93	0.00
Agt|44	3	2	0	0	1	3	2	1	1.25	5.63	5842	2.74	0.91
Agt|45	0	2	0	0	0	0	1	0	-	0.88	5816	3.89	1.95
Agt|46	1	0	1	1	0	1	0	2	0.33	5.65	5789	1.22	0.61
Agt|47	1	3	0	2	0	0	0	0	2.00	5.66	5816	1.82	0.00
Agt|48	1	1	1	2	0	0	0	0	0.67	5.66	5816	1.52	0.00
Agt|51	2	5	0	1	1	2	0	3	1.75	5.66	5762	3.37	0.92
Agt|53	0	1	1	2	0	4	1	1	0.14	5.66	5842	2.42	0.60
Agt|54	1	3	0	0	0	1	0	0	4.00	3.85	5789	2.25	0.00
Agt|55	3	4	0	1	1	1	1	2	2.33	5.63	5816	3.05	0.92
Agt|57	3	2	0	1	1	1	0	1	1.67	5.45	5816	2.52	0.32
Agt|58	0	3	0	1	2	2	0	1	0.60	5.65	5708	2.48	0.31
Agt|59	2	3	0	1	2	3	0	2	0.83	5.66	5816	3.34	0.61
Sum	77	160	23	63	35	67	18	32	--	--	--	--	--
Ave.	1.64	3.40	0.49	1.34	0.74	1.43	0.38	0.68	1.69	5.37	5819	2.92	0.36
SEM	0.20	0.26	0.11	0.16	0.13	0.19	0.08	0.13	0.21	0.12	5.74	0.15	0.06
Bs|1	1	2	4	1	0	0	1	1	0.60	4.29	5078	3.67	0.92
Bs|2	5	2	0	0	2	0	1	0	3.50	4.29	5059	4.15	0.46
Bs|3	1	2	1	0	1	1	1	1	1.00	4.29	5067	2.76	0.92

Bs|4	0	2	0	0	2	0	1	0	1.00	4.21	5100	1.86	0.47
Bs|6	3	0	0	0	0	0	2	3	-	4.29	5109	1.37	2.28
Bs|7	5	4	0	0	1	0	1	0	9.00	4.21	5100	4.66	0.47
Bs|8	3	1	0	1	1	1	1	3	1.33	4.20	5075	3.28	1.88
Bs|11	1	4	0	0	0	0	1	0	-	4.21	5067	2.34	0.47
Bs|13	1	1	0	0	0	0	0	1	-	4.21	5084	0.94	0.47
Bs|14	5	1	0	1	1	0	0	1	3.00	4.29	5100	3.66	0.46
Bs|15	3	3	0	0	3	0	1	0	2.00	4.29	5125	4.09	0.45
Bs|17	4	3	2	2	2	0	1	2	1.17	4.29	5067	5.98	1.38
Bs|19	1	2	2	0	1	0	0	2	1.00	4.29	5075	2.75	0.92
Bs|20	2	3	0	0	0	0	0	3	-	4.29	5042	2.31	1.39
Bs|22	2	5	0	0	0	0	0	4	-	4.21	5067	3.28	1.88
Bs|24	3	4	2	0	0	0	1	2	3.50	4.29	5100	4.11	1.37
Bs|26	3	1	0	1	3	0	1	3	1.00	4.21	5075	3.74	1.87
Bs|27	2	2	0	0	0	1	0	3	4.00	4.29	5084	2.29	1.38
Bs|28	1	1	1	0	0	0	0	3	2.00	4.29	5050	1.38	1.38
Bs|30	2	4	0	0	0	1	0	3	6.00	4.21	5042	3.30	1.41
Bs|31	5	2	0	0	0	0	1	2	-	4.29	5050	3.23	1.38
Bs|32	2	2	0	2	1	0	0	3	1.33	4.29	5100	3.20	1.37
Bs|35	4	1	0	0	0	0	1	3	-	4.29	5100	2.29	1.83
Bs|36	0	5	0	1	0	0	1	3	5.00	4.21	5092	2.80	1.87
Bs|37	1	3	0	0	0	1	0	2	4.00	4.29	5084	2.29	0.92
Bs|38	2	2	2	0	1	0	1	2	1.33	4.21	5059	3.29	1.41
Bs|39	4	5	1	1	0	0	0	4	4.50	4.29	5059	5.07	1.84
Bs|40	1	2	0	2	0	0	0	2	1.50	4.29	5050	2.31	0.92
Bs|41	2	1	0	1	0	0	1	1	3.00	4.21	5050	1.88	0.94
Bs|43	4	5	0	0	0	0	0	3	-	4.29	5109	4.11	1.37
Bs|44	13	5	3	0	0	0	0	1	6.00	4.21	5109	9.77	0.47
Bs|45	1	4	0	0	2	0	2	6	2.50	4.20	5100	3.26	3.73
Bs|46	2	3	0	2	1	0	0	3	1.67	4.29	5067	3.68	1.38
Bs|48	3	3	0	0	2	0	0	2	3.00	4.21	5050	3.77	0.94
Bs|49	3	1	0	1	0	0	1	1	4.00	4.21	5050	2.35	0.94
Bs|50	3	0	2	2	0	1	0	1	0.60	4.29	5042	3.70	0.46
Bs|51	3	1	2	1	0	0	1	2	1.33	4.21	5042	3.30	1.41
Bs|52	1	1	0	0	0	0	0	1	-	4.29	5109	0.91	0.46
Bs|53	4	4	1	1	0	0	0	0	4.00	4.21	5109	4.65	0.00
Bs|54	3	5	0	2	0	1	1	2	2.67	4.21	5092	5.13	1.40
Bs|55	7	4	0	0	2	1	2	2	3.67	4.21	5100	6.52	1.86
Bs|57	3	3	0	0	0	0	0	1	-	4.29	5092	2.75	0.46
Bs|60	3	3	0	1	0	0	0	2	6.00	4.19	5075	3.29	0.94
Bs|62	1	3	0	1	1	0	2	2	2.00	4.21	5075	2.81	1.87
Bs|63	3	2	3	1	0	0	2	1	1.25	4.21	5075	4.21	1.40
Bs|65	0	5	1	0	0	0	0	2	5.00	4.29	5067	2.76	0.92
Bs|68	5	3	1	0	0	0	0	2	8.00	4.29	5059	4.15	0.92
Bs|69	3	2	3	1	0	0	2	1	1.25	4.21	5075	4.21	1.40
Bs|71	0	5	1	0	0	0	0	2	5.00	4.29	5067	2.76	0.92
Bs|74	5	3	1	0	0	0	0	2	8.00	4.29	5059	4.15	0.92
Sum	139	135	33	26	27	8	31	96	--	--	--	--	--
Ave.	2.78	2.70	0.66	0.52	0.54	0.16	0.62	1.92	3.17	4.25	5077	3.41	1.18
SEM	0.31	0.21	0.15	0.10	0.12	0.05	0.10	0.17	0.31	0.01	3.16	0.21	0.09
Ec|1	0	0	0	0	0	0	0	0	-	4.62	3080	0.00	0.00
Ec|5	0	1	0	0	0	0	0	0	-	4.62	3080	0.70	0.00
Ec|6	2	4	0	0	0	1	1	1	6.00	4.60	6356	2.40	0.68
Ec|7	0	0	1	0	0	1	0	0	0.00	4.62	3080	1.41	0.00
Ec|10	0	1	2	0	1	0	0	0	0.33	4.62	3080	2.81	0.00
Ec|12	2	1	1	0	2	0	1	1	1.00	4.61	6356	2.05	0.68
Ec|14	2	2	1	0	0	0	1	2	4.00	4.61	6356	1.71	1.02
Ec|16	2	2	0	1	0	0	0	0	4.00	4.63	3080	3.51	0.00
Ec|17	1	2	0	1	0	0	0	1	3.00	4.56	6356	1.38	0.35
Ec|21	0	1	1	0	1	0	0	0	0.50	4.62	3080	2.11	0.00
Ec|23	2	1	1	0	0	0	0	0	3.00	4.62	3080	2.81	0.00
Ec|27	0	1	0	0	0	0	0	0	-	4.62	3080	0.70	0.00
Ec|28	2	3	1	0	4	0	0	2	1.00	4.62	6356	3.40	0.68
Ec|29	4	4	0	1	0	1	0	1	4.00	4.61	6356	3.42	0.34
Ec|35	0	3	0	2	2	0	0	1	0.75	4.59	6356	2.40	0.34
Ec|38	0	1	1	0	1	2	1	1	0.25	4.62	6356	1.70	0.68
Ec|39	4	5	2	0	1	0	1	1	3.00	4.62	6356	4.09	0.68

Ec|41	1	2	0	3	1	1	0	0	0.60	4.61	6356	2.73	0.00
Ec|42	0	0	0	0	0	0	0	0	-	4.62	3080	0.00	0.00
Ec|44	2	1	0	1	5	0	0	2	0.50	4.61	6356	3.07	0.68
Ec|45	0	2	2	1	2	1	1	3	0.33	4.60	6356	2.74	1.37
Ec|46	1	0	0	1	1	0	1	0	0.50	4.62	3080	2.11	0.70
Ec|49	4	0	0	2	2	0	0	2	1.00	4.62	6356	2.72	0.68
Ec|50	2	1	0	0	3	3	0	0	0.50	4.59	6356	3.08	0.00
Ec|51	0	0	0	0	0	0	0	0	-	4.62	3080	0.00	0.00
Ec|53	1	0	1	0	1	1	0	2	0.33	4.62	3080	2.81	1.41
Ec|55	0	2	0	0	0	1	0	0	2.00	4.62	3080	2.11	0.00
Ec|58	0	0	0	0	0	0	0	0	-	4.62	3080	0.00	0.00
Ec|59	2	3	0	0	3	0	1	0	1.67	4.61	6356	2.73	0.34
Ec|61	2	2	0	0	1	0	0	0	4.00	4.62	6356	1.70	0.00
Ec|62	0	2	0	0	1	0	0	1	2.00	4.61	6356	1.02	0.34
Ec|63	3	6	1	0	0	0	1	1	9.00	4.52	6356	3.48	0.70
Ec|65	1	4	0	1	0	1	1	2	2.50	4.61	6356	2.39	1.02
Ec|66	0	1	0	3	1	1	0	0	0.20	4.60	6356	2.05	0.00
Ec|67	0	1	0	0	0	0	0	0	-	4.62	3080	0.70	0.00
Ec|68	0	3	0	0	1	0	0	0	3.00	4.62	3080	2.81	0.00
Ec|69	0	1	0	1	0	0	0	0	1.00	4.62	3080	1.41	0.00
Ec|70	1	0	1	2	0	0	0	0	0.33	4.58	3080	2.84	0.00
Ec|71	1	0	0	0	1	0	1	0	1.00	4.62	3080	1.41	0.70
Ec|74	0	5	0	0	0	0	0	1	-	4.62	3080	3.52	0.70
Ec|75	1	0	0	0	1	0	0	0	1.00	4.62	3080	1.41	0.00
Ec|76	0	0	1	0	0	0	0	0	0.00	4.62	3080	0.70	0.00
Ec|78	1	1	0	0	0	0	0	0	-	4.62	3080	1.41	0.00
Ec|80	0	1	0	2	0	0	0	1	0.50	4.60	3080	2.12	0.71
Ec|81	0	3	0	1	0	0	1	0	3.00	4.62	3080	2.81	0.70
Ec|82	0	1	0	1	1	1	0	0	0.33	4.62	3080	2.81	0.00
Ec|83	0	2	1	0	0	0	0	0	2.00	4.62	3080	2.11	0.00
Ec|84	0	0	0	1	1	0	0	0	0.00	4.62	3080	1.41	0.00
Ec|85	0	3	2	3	0	0	0	0	0.60	4.62	3080	5.63	0.00
Ec|86	0	0	0	0	2	0	1	0	0.00	4.62	3080	1.41	0.70
Ec|87	0	0	0	2	0	0	0	0	0.00	4.62	3080	1.41	0.00
Ec|89	0	1	0	0	0	0	1	0	-	4.62	3080	0.70	0.70
Ec|90	0	2	0	0	0	0	1	0	-	4.62	3080	1.41	0.70
Ec|92	1	0	0	0	0	1	0	3	1.00	4.62	3080	1.41	2.11
Ec|93	1	0	0	0	0	0	0	0	-	4.62	3080	0.70	0.00
Ec|94	0	2	0	0	0	0	0	0	-	4.62	3080	1.41	0.00
Ec|96	0	1	0	1	0	0	0	1	1.00	4.62	3080	1.41	0.70
Ec|99	1	2	0	0	3	1	1	0	0.75	4.61	3080	4.92	0.70
Ec|100	0	0	0	0	0	0	2	0	-	4.60	6356	0.00	0.68
Sum	47	87	20	31	43	17	18	30	--	--	--	--	--
Ave.	0.80	1.47	0.34	0.53	0.73	0.29	0.31	0.51	1.59	4.61	4246	2.02	0.37
SEM	0.13	0.17	0.07	0.10	0.13	0.07	0.06	0.09	0.21	0.00	182.67	0.14	0.05
Mf|6	3	11	1	3	0	2	3	1	2.33	0.79	2332	1.08	0.22
Mf|11	2	4	0	4	0	2	1	3	1.00	0.79	2353	0.64	0.21
Mf|12	2	10	0	5	0	2	0	2	1.71	0.79	2396	1.00	0.11
Mf|14	2	7	0	4	0	0	3	3	2.25	0.79	2353	0.70	0.32
Mf|19	2	8	1	8	0	6	1	4	0.67	0.79	2268	1.39	0.28
Mf|23	2	9	1	3	1	1	3	2	1.83	0.79	2290	0.94	0.28
Mf|26	4	4	0	7	0	0	1	2	1.14	0.79	2311	0.82	0.16
Mf|39	0	8	1	6	1	1	4	2	0.89	0.79	2374	0.90	0.32
Mf|41	3	4	0	4	0	2	2	4	1.17	0.79	2374	0.69	0.32
Mf|42	0	6	1	5	0	0	3	2	1.00	0.79	2353	0.64	0.27
Mf|47	1	10	0	3	1	3	1	2	1.57	0.79	2374	0.96	0.16
Mf|49	1	5	0	7	1	5	1	4	0.46	0.79	2353	1.02	0.27
Mf|50	4	4	1	4	0	5	1	1	0.80	0.79	2332	0.98	0.11
Mf|55	3	5	0	8	0	3	2	2	0.73	0.79	2396	1.00	0.21
Mf|56	3	5	0	6	0	2	1	7	1.00	0.79	2374	0.85	0.43
Mf|57	1	7	1	4	2	1	2	4	1.00	0.79	2353	0.86	0.32
Mf|58	3	38	2	4	0	4	2	4	4.10	0.79	2374	2.73	0.32
Mf|59	1	9	0	4	0	2	3	1	1.67	0.79	2374	0.85	0.21
Mf|62	1	8	0	2	0	3	1	2	1.80	0.79	2374	0.74	0.16
Mf|65	0	12	1	3	1	1	3	0	2.00	0.79	2353	0.97	0.16
Mf|67	1	9	0	3	0	1	4	2	2.50	0.79	2353	0.75	0.32
Mf|68	1	13	1	1	0	0	1	2	7.00	0.79	2374	0.85	0.16

Mf|76	3	9	1	6	2	3	2	2	1.00	0.79	2310	1.31	0.22
Mf|83	1	6	0	1	0	6	2	3	1.00	0.79	2332	0.76	0.27
Mf|85	1	7	1	5	0	1	2	1	1.14	0.79	2353	0.80	0.16
Mf|90	3	2	0	5	2	5	0	1	0.42	0.79	2311	0.93	0.05
Mf|92	3	8	0	3	0	1	2	4	2.75	0.79	2374	0.80	0.32
Mf|93	1	5	0	10	1	3	0	1	0.43	0.79	2396	1.05	0.05
Mf|99	0	6	3	8	0	4	1	5	0.40	0.79	2311	1.15	0.33
Sum	52	239	16	136	12	69	52	73	--	--	--	--	--
Ave.	1.79	8.24	0.55	4.69	0.41	2.38	1.79	2.52	1.58	0.79	2351	0.97	0.23
SEM	0.22	1.17	0.14	0.40	0.13	0.33	0.21	0.28	0.25	0.00	5.98	0.07	0.02
Se|2	1	4	0	7	1	0	2	0	0.63	2.56	7103	7.16	1.10
Se|3	1	2	3	5	0	1	1	0	0.33	2.54	7103	6.65	0.55
Se|4	2	7	6	1	1	0	2	0	1.13	2.54	7103	9.44	1.11
Se|7	4	4	4	6	0	0	1	0	0.80	2.54	7053	10.06	0.56
Se|8	1	5	1	1	0	0	1	2	3.00	2.55	7053	4.44	1.66
Se|10	4	4	2	7	1	0	2	1	0.80	2.55	7103	9.95	1.66
Se|11	2	2	1	6	0	0	2	0	0.57	2.55	7128	6.06	1.10
Se|12	3	3	1	4	1	0	4	0	1.00	2.55	7103	6.62	2.21
Se|13	0	8	3	2	0	0	1	2	1.60	2.55	7128	7.14	1.65
Se|34	0	2	3	4	0	1	1	3	0.25	2.54	7103	5.55	2.22
Se|38	2	3	1	4	1	1	0	0	0.71	2.56	7103	6.61	0.00
Se|39	2	5	3	3	0	0	2	3	1.17	2.52	7103	7.27	2.79
Se|50	2	2	2	4	4	0	0	2	0.40	2.53	7128	7.75	1.11
Se|59	2	8	2	2	3	2	0	0	1.11	2.55	7128	10.45	0.00
Se|62	2	8	4	4	3	0	0	0	0.91	2.55	7103	11.57	0.00
Se|63	0	0	0	1	3	0	0	0	0.00	2.56	7128	2.20	0.00
Se|65	0	0	1	0	0	1	0	2	0.00	2.55	7103	1.10	1.10
Se|66	2	6	2	2	0	0	1	2	2.00	2.55	7103	6.62	1.65
Se|67	1	1	1	3	1	1	1	2	0.33	2.55	7103	4.41	1.65
Se|69	0	6	4	3	0	0	0	1	0.86	2.54	7103	7.19	0.55
Se|70	3	6	4	4	1	0	0	1	1.00	2.55	7078	9.99	0.55
Se|75	0	9	5	6	2	4	2	1	0.53	2.53	7053	14.57	1.68
Sum	34	95	53	79	22	11	23	22	--	--	--	--	--
Ave.	1.55	4.32	2.41	3.59	1.00	0.50	1.05	1.00	0.87	2.55	7101	7.40	1.13
SEM	0.28	0.59	0.35	0.44	0.27	0.21	0.23	0.23	0.15	0.00	5.05	0.66	0.17
Vc|1	0	4	0	0	0	0	0	1	-	3.83	6453	1.62	0.40
Vc|4	0	2	1	1	0	0	0	1	1.00	3.83	6453	1.62	0.40
Vc|5	1	0	0	1	0	0	0	0	1.00	3.82	6453	0.81	0.00
Vc|7	0	0	0	0	0	0	0	1	-	3.83	6453	0.00	0.40
Vc|8	0	1	0	1	0	0	0	0	1.00	3.83	6453	0.81	0.00
Vc|9	0	2	0	0	0	0	0	1	-	3.83	6453	0.81	0.40
Vc|10	1	1	0	0	0	0	0	0	-	3.84	6453	0.81	0.00
Vc|11	0	2	0	0	1	0	0	0	2.00	3.82	6453	1.22	0.00
Vc|15	0	0	0	0	0	0	0	0	-	3.81	6453	0.00	0.00
Vc|16	1	2	1	0	0	1	0	0	1.50	3.82	6453	2.03	0.00
Vc|17	1	1	0	1	0	0	0	0	2.00	3.83	6453	1.21	0.00
Vc|19	0	1	0	3	1	0	0	0	0.25	3.80	6453	2.04	0.00
Vc|20	0	1	0	0	0	0	0	0	-	3.82	6453	0.41	0.00
Vc|22	0	3	0	0	0	0	0	0	-	3.82	6453	1.22	0.00
Vc|23	0	4	0	1	0	0	0	1	4.00	3.83	6453	2.02	0.40
Vc|25	0	1	0	1	0	0	0	1	1.00	3.81	6453	0.81	0.41
Vc|26	0	0	1	1	0	0	0	1	0.00	3.82	6453	0.81	0.41
Vc|27	0	0	0	0	0	0	0	0	-	3.83	6453	0.00	0.00
Vc|28	0	1	0	0	0	0	0	0	-	3.83	6453	0.40	0.00
Vc|29	1	0	0	0	1	0	1	0	1.00	3.80	6453	0.81	0.41
Vc|30	0	2	0	0	2	0	0	0	1.00	3.84	6453	1.62	0.00
Vc|31	0	2	0	3	2	0	0	0	0.40	3.84	6453	2.83	0.00
Vc|32	1	0	0	0	0	0	0	1	-	3.84	6453	0.40	0.40
Vc|33	1	2	1	1	0	0	0	0	1.50	3.84	6453	2.02	0.00
Vc|34	0	2	0	1	0	1	0	0	1.00	3.83	6453	1.62	0.00
Vc|35	1	2	0	0	1	1	0	0	1.50	3.83	6453	2.02	0.00
Vc|37	0	1	0	1	0	0	0	0	1.00	3.83	6453	0.81	0.00
Vc|39	0	3	2	1	1	0	0	1	0.75	3.83	6453	2.83	0.40
Vc|41	0	0	0	1	0	0	0	0	0.00	3.82	6453	0.41	0.00
Vc|43	1	1	1	0	0	0	0	0	2.00	3.84	6453	1.21	0.00
Vc|45	1	2	0	0	0	1	0	0	3.00	3.83	6453	1.62	0.00
Vc|47	0	2	0	1	0	0	1	0	2.00	3.84	6453	1.21	0.40

Vc|48	1	2	0	1	1	1	0	0	1.00	3.81	6453	2.44	0.00
Vc|49	1	0	0	1	0	0	0	1	1.00	3.82	6453	0.81	0.41
Vc|51	0	2	0	0	2	0	0	1	1.00	3.83	6453	1.62	0.40
Vc|52	0	0	0	0	1	0	0	0	0.00	3.84	6453	0.40	0.00
Vc|53	0	1	0	0	2	0	1	1	0.50	3.82	6453	1.22	0.81
Vc|57	1	1	0	0	1	0	0	1	2.00	3.82	6453	1.22	0.41
Vc|58	0	0	0	0	0	0	0	1	-	3.84	6453	0.00	0.40
Vc|59	1	3	0	0	1	0	0	0	4.00	3.83	6453	2.02	0.00
Vc|61	1	0	0	0	1	0	0	0	1.00	3.84	6453	0.81	0.00
Vc|63	1	2	0	0	0	0	0	1	-	3.83	6453	1.21	0.40
Vc|65	0	0	0	1	0	1	0	1	0.00	3.82	6453	0.81	0.41
Vc|66	0	0	0	1	0	0	0	0	0.00	3.83	6453	0.40	0.00
Vc|68	1	0	0	0	0	0	0	0	-	3.82	6453	0.41	0.00
Vc|70	1	2	0	0	1	0	0	1	3.00	3.83	6453	1.62	0.40
Sum	18	58	7	23	19	6	3	17	--	--	--	--	--
Ave.	0.39	1.26	0.15	0.50	0.41	0.13	0.07	0.37	1.28	3.83	6453	1.15	0.18
SEM	0.07	0.17	0.06	0.11	0.10	0.05	0.04	0.07	0.16	0.00	0.00	0.11	0.03
