# Supplementary material for: Evolution of the Insertion-Deletion Mutation Rate Across the Tree of Life
Source: G3 (Bethesda). 2016 Jun 15;6(8):2583–91. doi: 10.1534/g3.116.030890 (PMC4978911; doi:10.1534/g3.116.030890)
Supplement: Supplemental Material [file supp_g3.116.030890_DatasetS2.pdf]

Dataset S2.

Caenorhabditis elegans N2 insertion-deletion (indel) summary statistics after an average of 250 generations (Gen). SEM is the standard error.

MA Line	Indels		Sites (× 10^6)	Gen.	Indel Rate (× 10^{-10}) /events /gen.
	Ins.	Del.			
Ce|523	5	8	64.5	247	8.16
Ce|526	3	0	60.5	245	2.03
Ce|529	9	4	82.4	256	6.16
Ce|538	9	8	83.5	250	8.16
Ce|535	0	0	56.2	256	0.00
Ce|553	3	3	77.5	241	2.68
Ce|574	21	8	86.5	258	13.0
Sum	50	31	--	--	--
Ave.	7.14	4.43	73.01	250	5.74
SEM	2.83	1.49	5.03	2.63	1.83
