# Supplementary material for: Evolution of the Insertion-Deletion Mutation Rate Across the Tree of Life
Source: G3 (Bethesda). 2016 Jun 15;6(8):2583–91. doi: 10.1534/g3.116.030890 (PMC4978911; doi:10.1534/g3.116.030890)
Supplement: Supplemental Material [file supp_g3.116.030890_DatasetS3.pdf]

Dataset S3.

List of base-substitution mutation calls for *Agrobacterium tumefaciens* (Agt), *Bacillus subtilis* (Bs), *Escherichia coli* (Ec), *Mesoplasma florum* (Mf), and *Vibrio cholerae* (Vc). Line column indicates the organism and MA line where indel is called seperated by (|). IG - intergenic, EX - exon, syn - synonymous, nonsyn - nonsynonymous. Gene is the gene name or type of gene where the base substitution occurs. Confirmed (conf.) column indicates if the mutation was verified using direct sequencing (seq).

Line	Scaffold:Position	Mut	Type	Codon Change	Amino Acid Change	Gene	syn nonsyn	Conf.
Agt|11	NC_003062:2110	G>T	EX	GCG>TCG	A>S	shikimate	nonsyn	
Agt|59	NC_003064:3426	A>G	EX	GAG>GGG	E>G	replication	nonsyn	
Agt|14	NC_003065:6186	G>A	EX	GGG>GGA	G>G	C'	syn	
Agt|33	NC_003062:7280	G>A	EX	GGC>AGC	G>S	hypothetical	nonsyn	
Agt|55	NC_003063:7913	C>T	IG					
Agt|54	NC_003062:9249	G>A	EX	GCC>ACC	A>T	DNA	nonsyn	
Agt|54	NC_003062:10200	G>C	EX	GTT>CTT	V>L	DNA	nonsyn	
Agt|19	NC_003062:13517	C>T	EX	GGC>GGT	G>G	intracellular	syn	
Agt|13	NC_003063:13737	T>C	EX	GTC>GCC	V>A	sugar	nonsyn	
Agt|34	NC_003062:15711	C>T	EX	ACC>ACT	T>T	tryptophan	syn	
Agt|6	NC_003065:18170	C>G	EX	CCG>CGG	P>R	tryptophan	nonsyn	
Agt|20	NC_003062:20222	C>T	EX	CCG>CCA	P>P	ATP-dependant	syn	
Agt|25	NC_003064:20373	C>T	EX	ATG>ATA	M>I	hypothetical	nonsyn	
Agt|42	NC_003065:23616	T>A	EX	GAA>GTA	E>V	D-nopaline	nonsyn	
Agt|7	NC_003062:38383	C>A	EX	GGC>GGA	G>G	phosphoenolpyruvate	syn	
Agt|20	NC_003064:38970	A>G	IG					
Agt|40	NC_003063:40424	G>A	EX	GAA>AAA	E>K	hydantoinase	nonsyn	
Agt|36	NC_003063:41161	A>G	EX	ACG>GCG	T>A	asparaginase	nonsyn	
Agt|33	NC_003063:45990	A>G	EX	TGC>CGC	C>R	oligopeptide	nonsyn	
Agt|55	NC_003064:46303	T>C	IG					
Agt|55	NC_003065:46430	C>A	EX	GAG>TAG	E>*	conjugal	nonsyn	
Agt|51	NC_003062:47306	C>G	EX	GGC>GGG	G>G	hypothetical	syn	
Agt|10	NC_003063:51054	A>G	EX	ATG>ACG	M>T	peptide	nonsyn	
Agt|43	NC_003063:53886	G>A	EX	GTC>GTT	V>V	hypothetical	syn	
Agt|40	NC_003062:54600	C>T	EX	CTG>CTA	L>L	helicase	syn	
Agt|24	NC_003065:56650	T>G	EX	GCA>GCC	A>A	DNA	syn	
Agt|51	NC_003063:58636	C>A	IG					
Agt|22	NC_003065:69034	C>G	EX	CCA>CGA	P>R	ABC	nonsyn	
Agt|2	NC_003064:69952	C>T	IG					
Agt|45	NC_003062:79525	G>A	EX	CCG>TCG	P>S	D-isomer	nonsyn	
Agt|30	NC_003065:81832	C>T	EX	GTA>ATA	V>I	ABC	nonsyn	
Agt|53	NC_003064:101622	G>C	EX	CTC>CTG	L>L	ATP-dependent	syn	
Agt|5	NC_003062:105414	C>T	IG					
Agt|40	NC_003065:107215	T>C	EX	GAT>GAC	D>D	hypothetical	syn	
Agt|48	NC_003063:108500	G>A	EX	ACC>ATC	T>I	sugar	nonsyn	
Agt|54	NC_003063:113162	T>C	IG					
Agt|16	NC_003064:118893	A>C	EX	ATC>CTC	I>L	conjugal	nonsyn	
Agt|2	NC_003064:124727	A>T	EX	GTG>GAG	V>E	hypothetical	nonsyn	
Agt|55	NC_003064:124997	T>C	IG					
Agt|46	NC_003065:125393	A>T	EX	GAT>GTT	D>V	SNF2	nonsyn	
Agt|33	NC_003063:125935	A>G	IG					
Agt|43	NC_003064:129638	G>T	EX	CCC>ACC	P>T	acetolactate	nonsyn	
Agt|17	NC_003064:133911	G>T	EX	GGG>TGG	G>W	ABC	nonsyn	
Agt|9	NC_003063:133957	C>A	EX	GCA>TCA	A>S	acetyltransferase	nonsyn	

<table>
<tr><td>Agt|14</td><td>NC_003065:134655</td><td>G>T</td><td>EX</td><td>ACC>ACA</td><td>T>T</td><td>hypothetical</td><td>syn</td></tr>
<tr><td>Agt|9</td><td>NC_003062:138112</td><td>G>A</td><td>EX</td><td>ACC>ACT</td><td>T>T</td><td>hypothetical</td><td>syn</td></tr>
<tr><td>Agt|10</td><td>NC_003063:146320</td><td>G>A</td><td>EX</td><td>GCC>GCT</td><td>A>A</td><td>hypothetical</td><td>syn</td></tr>
<tr><td>Agt|41</td><td>NC_003062:148488</td><td>C>A</td><td>EX</td><td>ATC>ATA</td><td>I>I</td><td>hypothetical</td><td>syn</td></tr>
<tr><td>Agt|9</td><td>NC_003063:149329</td><td>G>A</td><td>EX</td><td>GCC>GTC</td><td>A>V</td><td>2-deoxy-D-gluconate</td><td>nonsyn</td></tr>
<tr><td>Agt|53</td><td>NC_003063:150802</td><td>A>T</td><td>IG</td><td></td><td></td><td></td><td></td></tr>
<tr><td>Agt|7</td><td>NC_003062:157731</td><td>C>T</td><td>EX</td><td>CCG>CCA</td><td>P>P</td><td>enoyl-(acyl-carrier-protein)</td><td>syn</td></tr>
<tr><td>Agt|5</td><td>NC_003065:161987</td><td>T>C</td><td>EX</td><td>TTG>TCG</td><td>L>S</td><td>ABC</td><td>nonsyn</td></tr>
<tr><td>Agt|2</td><td>NC_003062:163548</td><td>G>A</td><td>EX</td><td>CCG>CCA</td><td>P>P</td><td>ABC</td><td>syn</td></tr>
<tr><td>Agt|32</td><td>NC_003062:164790</td><td>C>T</td><td>EX</td><td>ATC>ATT</td><td>I>I</td><td>ABC</td><td>syn</td></tr>
<tr><td>Agt|22</td><td>NC_003065:165904</td><td>G>T</td><td>EX</td><td>GAT>TAT</td><td>D>Y</td><td>hypothetical</td><td>nonsyn</td></tr>
<tr><td>Agt|59</td><td>NC_003065:169532</td><td>G>C</td><td>EX</td><td>TGG>TCG</td><td>W>S</td><td>P-450</td><td>nonsyn</td></tr>
<tr><td>Agt|25</td><td>NC_003063:170691</td><td>G>A</td><td>EX</td><td>ATC>ATT</td><td>I>I</td><td>hypothetical</td><td>syn</td></tr>
<tr><td>Agt|30</td><td>NC_003062:173021</td><td>C>A</td><td>EX</td><td>GTT>TTT</td><td>V>F</td><td>hypothetical</td><td>nonsyn</td></tr>
<tr><td>Agt|16</td><td>NC_003064:183921</td><td>T>G</td><td>IG</td><td></td><td></td><td></td><td></td></tr>
<tr><td>Agt|42</td><td>NC_003063:194294</td><td>A>C</td><td>IG</td><td></td><td></td><td></td><td></td></tr>
<tr><td>Agt|16</td><td>NC_003063:196816</td><td>C>A</td><td>EX</td><td>GCC>GCA</td><td>A>A</td><td>glycerol-3-phosphate</td><td>syn</td></tr>
<tr><td>Agt|40</td><td>NC_003065:197439</td><td>G>T</td><td>EX</td><td>GCA>TCA</td><td>A>S</td><td>type</td><td>nonsyn</td></tr>
<tr><td>Agt|47</td><td>NC_003062:208230</td><td>C>T</td><td>IG</td><td></td><td></td><td></td><td></td></tr>
<tr><td>Agt|15</td><td>NC_003065:210421</td><td>G>A</td><td>IG</td><td></td><td></td><td></td><td></td></tr>
<tr><td>Agt|44</td><td>NC_003062:215751</td><td>A>C</td><td>EX</td><td>ACC>CCC</td><td>T>P</td><td>hypothetical</td><td>nonsyn</td></tr>
<tr><td>Agt|17</td><td>NC_003063:221868</td><td>G>A</td><td>EX</td><td>GGC>GAC</td><td>G>D</td><td>diguanylate</td><td>nonsyn</td></tr>
<tr><td>Agt|59</td><td>NC_003063:226233</td><td>T>C</td><td>IG</td><td></td><td></td><td></td><td></td></tr>
<tr><td>Agt|53</td><td>NC_003064:241750</td><td>G>T</td><td>EX</td><td>CCC>CCA</td><td>P>P</td><td>amidase</td><td>syn</td></tr>
<tr><td>Agt|33</td><td>NC_003064:247699</td><td>G>A</td><td>EX</td><td>GCC>GCT</td><td>A>A</td><td>pyridoxal</td><td>syn</td></tr>
<tr><td>Agt|30</td><td>NC_003063:250757</td><td>G>A</td><td>EX</td><td>ACC>ATC</td><td>T>I</td><td>NAD</td><td>nonsyn</td></tr>
<tr><td>Agt|29</td><td>NC_003062:252718</td><td>T>C</td><td>IG</td><td></td><td></td><td></td><td></td></tr>
<tr><td>Agt|10</td><td>NC_003064:252969</td><td>C>T</td><td>EX</td><td>GCT>GTT</td><td>A>V</td><td>ABC</td><td>nonsyn</td></tr>
<tr><td>Agt|19</td><td>NC_003064:253000</td><td>C>T</td><td>EX</td><td>ACC>ACT</td><td>T>T</td><td>ABC</td><td>syn</td></tr>
<tr><td>Agt|11</td><td>NC_003063:256863</td><td>G>A</td><td>IG</td><td></td><td></td><td></td><td></td></tr>
<tr><td>Agt|16</td><td>NC_003064:271545</td><td>T>C</td><td>EX</td><td>CGA>CGG</td><td>R>R</td><td>hypothetical</td><td>syn</td></tr>
<tr><td>Agt|40</td><td>NC_003064:276193</td><td>T>C</td><td>EX</td><td>GAT>GGT</td><td>D>G</td><td>dehydrogenase</td><td>nonsyn</td></tr>
<tr><td>Agt|43</td><td>NC_003064:287122</td><td>G>A</td><td>EX</td><td>GGA>AGA</td><td>G>R</td><td>dehydrogenase</td><td>nonsyn</td></tr>
<tr><td>Agt|58</td><td>NC_003064:288460</td><td>C>T</td><td>EX</td><td>CTG>TTG</td><td>L>L</td><td>TetR</td><td>syn</td></tr>
<tr><td>Agt|28</td><td>NC_003064:291087</td><td>C>T</td><td>EX</td><td>CCC>CCT</td><td>P>P</td><td>arylester</td><td>syn</td></tr>
<tr><td>Agt|59</td><td>NC_003062:291368</td><td>G>A</td><td>EX</td><td>TTC>TTT</td><td>F>F</td><td>hypothetical</td><td>syn</td></tr>
<tr><td>Agt|38</td><td>NC_003063:292400</td><td>G>A</td><td>EX</td><td>GTG>ATG</td><td>V>M</td><td>exopolysaccharide</td><td>nonsyn</td></tr>
<tr><td>Agt|43</td><td>NC_003062:292441</td><td>C>G</td><td>EX</td><td>GTG>CTG</td><td>V>L</td><td>hypothetical</td><td>nonsyn</td></tr>
<tr><td>Agt|33</td><td>NC_003062:294372</td><td>G>A</td><td>EX</td><td>GGC>GGT</td><td>G>G</td><td>DNA</td><td>syn</td></tr>
<tr><td>Agt|59</td><td>NC_003064:305818</td><td>C>G</td><td>EX</td><td>CAC>GAC</td><td>H>D</td><td>TonB-dependent</td><td>nonsyn</td></tr>
<tr><td>Agt|57</td><td>NC_003063:306404</td><td>T>C</td><td>EX</td><td>ATC>ACC</td><td>I>T</td><td>ABC</td><td>nonsyn</td></tr>
<tr><td>Agt|55</td><td>NC_003064:309783</td><td>A>C</td><td>IG</td><td></td><td></td><td></td><td></td></tr>
<tr><td>Agt|10</td><td>NC_003063:310028</td><td>C>T</td><td>EX</td><td>CTG>TTG</td><td>L>L</td><td>sugar</td><td>syn</td></tr>
<tr><td>Agt|11</td><td>NC_003064:311752</td><td>A>G</td><td>IG</td><td></td><td></td><td></td><td></td></tr>
<tr><td>Agt|51</td><td>NC_003062:316640</td><td>C>T</td><td>EX</td><td>CTG>TTG</td><td>L>L</td><td>30S</td><td>syn</td></tr>
<tr><td>Agt|41</td><td>NC_003063:317684</td><td>A>G</td><td>IG</td><td></td><td></td><td></td><td></td></tr>
<tr><td>Agt|7</td><td>NC_003063:317823</td><td>C>A</td><td>EX</td><td>GCC>GAC</td><td>A>D</td><td>alanine</td><td>nonsyn</td></tr>
<tr><td>Agt|28</td><td>NC_003063:322604</td><td>G>A</td><td>EX</td><td>CCC>CTC</td><td>P>L</td><td>two</td><td>nonsyn</td></tr>
<tr><td>Agt|16</td><td>NC_003064:327134</td><td>C>T</td><td>EX</td><td>ATC>ATT</td><td>I>I</td><td>dehydrogenase</td><td>syn</td></tr>
<tr><td>Agt|24</td><td>NC_003062:348534</td><td>A>G</td><td>EX</td><td>AAC>AGC</td><td>N>S</td><td>ferric</td><td>nonsyn</td></tr>
<tr><td>Agt|21</td><td>NC_003064:354538</td><td>A>C</td><td>EX</td><td>TGC>GGC</td><td>C>G</td><td>aquaporin</td><td>nonsyn</td></tr>
<tr><td>Agt|13</td><td>NC_003062:360745</td><td>A>G</td><td>IG</td><td></td><td></td><td></td><td></td></tr>
<tr><td>Agt|32</td><td>NC_003063:362300</td><td>A>T</td><td>EX</td><td>GCT>GCA</td><td>A>A</td><td>hypothetical</td><td>syn</td></tr>
<tr><td>Agt|27</td><td>NC_003064:364836</td><td>A>T</td><td>IG</td><td></td><td></td><td></td><td></td></tr>
<tr><td>Agt|44</td><td>NC_003062:370135</td><td>G>A</td><td>EX</td><td>GGC>GGT</td><td>G>G</td><td>inosine-uridine</td><td>syn</td></tr>
<tr><td>Agt|30</td><td>NC_003063:376491</td><td>C>T</td><td>EX</td><td>GTC>GTT</td><td>V>V</td><td>trehalose/maltose</td><td>syn</td></tr>
<tr><td>Agt|7</td><td>NC_003063:390528</td><td>T>A</td><td>EX</td><td>GGA>GGT</td><td>G>G</td><td>pyrroloquinoline-quinone-dependent</td><td>syn</td></tr>
<tr><td>Agt|2</td><td>NC_003064:399468</td><td>T>G</td><td>IG</td><td></td><td></td><td></td><td></td></tr>
<tr><td>Agt|14</td><td>NC_003064:400601</td><td>A>C</td><td>IG</td><td></td><td></td><td></td><td></td></tr>
</table>

Agt|20	NC_003063:416529	A>T	EX	TGG>AGG	W>R	peptide	nonsyn
Agt|14	NC_003064:419113	A>G	EX	GAC>GGC	D>G	oxidoreductase	nonsyn
Agt|4	NC_003064:423797	C>T	IG				
Agt|59	NC_003064:434276	T>G	EX	CTT>CGT	L>R	ABC	nonsyn
Agt|40	NC_003064:437254	A>G	IG				
Agt|30	NC_003062:438032	T>C	IG				
Agt|5	NC_003063:448065	C>A	EX	CCC>ACC	P>T	succinate	nonsyn
Agt|30	NC_003063:456551	T>C	EX	CTG>CCG	L>P	ABC	nonsyn
Agt|26	NC_003063:469175	C>T	EX	GCG>GCA	A>A	monooxygenase	syn
Agt|53	NC_003064:470214	C>T	EX	GCG>ACG	A>T	chloromuconate	nonsyn
Agt|38	NC_003064:471392	C>T	EX	GCC>ACC	A>T	acetylornithine	nonsyn
Agt|48	NC_003064:472081	T>C	EX	AAC>AGC	N>S	acetylornithine	nonsyn
Agt|59	NC_003064:474764	T>G	IG				
Agt|41	NC_003062:478067	T>A	IG				
Agt|17	NC_003064:478655	C>T	EX	CGC>TGC	R>C	hypothetical	nonsyn
Agt|17	NC_003062:494085	T>C	EX	CGT>CGC	R>R	enoyl-CoA	syn
Agt|24	NC_003062:495774	G>C	EX	GAG>GAC	E>D	hypothetical	nonsyn
Agt|28	NC_003063:512307	C>T	EX	GGC>GGT	G>G	dehydrogenase	syn
Agt|33	NC_003064:514069	C>G	EX	GGT>GCT	G>A	hypothetical	nonsyn
Agt|13	NC_003063:517433	A>C	EX	CTT>CTG	L>L	phosphoglyceromutase	syn
Agt|43	NC_003062:518364	C>T	EX	ACG>ACA	T>T	two	syn
Agt|13	NC_003064:526869	C>T	EX	ACG>ATG	T>M	ABC	nonsyn
Agt|14	NC_003064:532724	C>T	EX	GGA>AGA	G>R	mercuric	nonsyn
Agt|43	NC_003062:541096	A>T	EX	GTC>GAC	V>D	flagellar	nonsyn
Agt|2	NC_003062:545362	A>C	EX	ATG>AGG	M>R	hypothetical	nonsyn
Agt|34	NC_003062:558515	C>T	EX	CTG>TTG	L>L	transcriptional	syn
Agt|14	NC_003063:563131	G>T	EX	GGC>GGA	G>G	hypothetical	syn
Agt|13	NC_003063:572431	C>A	EX	CTT>ATT	L>I	polysaccharide	nonsyn
Agt|53	NC_003062:579436	C>G	EX	GGC>GGG	G>G	alpha-glucosidase	syn
Agt|40	NC_003062:585964	T>G	EX	AAG>ACG	K>T	glucose-6-phosphate	nonsyn
Agt|57	NC_003063:588700	G>A	IG				
Agt|33	NC_003062:591613	G>T	IG				
Agt|57	NC_003062:602978	G>C	EX	ACC>ACG	T>T	two	syn
Agt|9	NC_003062:623347	C>G	EX	GCG>GCC	A>A	octaprenyl-diphosphate	syn
Agt|15	NC_003062:633577	C>A	EX	CTG>ATG	L>M	glycyl-tRNA	nonsyn
Agt|37	NC_003063:652176	G>T	EX	GGT>GTT	G>V	3-hydroxybutyryl-CoA	nonsyn
Agt|58	NC_003062:652258	T>G	EX	GAA>GAC	E>D	phosphate	nonsyn
Agt|38	NC_003063:653177	C>G	EX	TCA>TGA	S>*	argininosuccinate	nonsyn
Agt|38	NC_003063:653178	A>C	EX	TCA>TCC	S>S	argininosuccinate	syn
Agt|5	NC_003062:660105	T>G	IG				
Agt|46	NC_003063:661017	C>A	EX	GAT>TAT	D>Y	hypothetical	nonsyn
Agt|29	NC_003062:661699	T>C	IG				
Agt|41	NC_003063:675986	C>A	EX	CCG>CCT	P>P	cobyrinic	syn
Agt|9	NC_003063:676200	G>A	EX	GCG>GTG	A>V	cobyrinic	nonsyn
Agt|43	NC_003062:684364	T>C	EX	GAT>GAC	D>D	hypothetical	syn
Agt|51	NC_003063:698722	A>C	EX	CAA>CAC	Q>H	hypothetical	nonsyn
Agt|6	NC_003062:699323	A>C	EX	CAG>CCG	Q>P	two	nonsyn
Agt|9	NC_003062:714219	A>G	EX	CAC>CGC	H>R	hypothetical	nonsyn
Agt|11	NC_003062:740198	C>T	EX	GCG>GTG	A>V	transcriptional	nonsyn
Agt|29	NC_003062:754609	T>C	EX	GAC>GGC	D>G	enoyl-(acyl-carrier-protein)	nonsyn
Agt|38	NC_003062:758571	C>T	IG				
Agt|10	NC_003062:758811	G>C	EX	GCC>CCC	A>P	hypothetical	nonsyn
Agt|22	NC_003062:767514	G>T	EX	TGC>TTC	C>F	cytochrome	nonsyn
Agt|44	NC_003063:775685	C>G	EX	CTG>CTC	L>L	hypothetical	syn
Agt|10	NC_003063:779726	A>G	IG				
Agt|34	NC_003063:788218	A>G	IG				
Agt|29	NC_003063:791250	G>A	EX	GCA>GTA	A>V	metalloprotease	nonsyn
Agt|24	NC_003062:792433	A>G	EX	ACT>ACC	T>T	putative	syn
Agt|4	NC_003062:820596	T>C	EX	GTC>GCC	V>A	ABC	nonsyn

Agt|55	NC_003063:822696	G>C	EX	GAA>CAA	E>Q	fructose	nonsyn
Agt|58	NC_003062:842511	G>C	IG				
Agt|20	NC_003062:843560	A>G	EX	TGG>CGG	W>R	P	nonsyn
Agt|32	NC_003063:846339	G>A	EX	CGC>CGT	R>R	sigma	syn
Agt|27	NC_003062:850675	A>T	EX	GGT>GGA	G>G	ABC	syn
Agt|39	NC_003063:860579	C>T	EX	CAG>TAG	Q>*	hypothetical	nonsyn
Agt|30	NC_003063:873550	A>G	EX	GAC>GGC	D>G	threonine	nonsyn
Agt|5	NC_003062:881511	C>T	IG				
Agt|53	NC_003063:884801	G>C	EX	TTC>TTG	F>L	L-asparagine	nonsyn
Agt|20	NC_003062:892265	A>G	EX	ACC>GCC	T>A	6-O-methylguanine-DNA	nonsyn
Agt|39	NC_003062:902547	C>A	EX	ACC>AAC	T>N	Na+/H+	nonsyn
Agt|9	NC_003062:906024	A>G	IG				
Agt|6	NC_003062:912073	G>C	EX	GCC>GCG	A>A	hypothetical	syn
Agt|2	NC_003062:918237	G>C	EX	CAG>GAG	Q>E	amide	nonsyn
Agt|17	NC_003062:927723	C>A	EX	ACG>AAG	T>K	copper	nonsyn
Agt|28	NC_003062:928847	G>C	IG				
Agt|27	NC_003063:932057	G>C	EX	CCT>CGT	P>R	hypothetical	nonsyn
Agt|13	NC_003062:937948	C>G	EX	GCC>GGC	A>G	hypothetical	nonsyn
Agt|42	NC_003062:940069	T>A	IG				
Agt|4	NC_003062:940210	C>T	EX	GAC>GAT	D>D	hypothetical	syn
Agt|32	NC_003063:959774	C>A	EX	ATC>ATA	I>I	hypothetical	syn
Agt|42	NC_003062:961875	G>A	EX	GGC>GAC	G>D	cycH	nonsyn
Agt|19	NC_003062:970181	C>G	IG				
Agt|2	NC_003063:976973	G>A	EX	CCC>CTC	P>L	sugar	nonsyn
Agt|51	NC_003063:977775	A>G	IG				
Agt|42	NC_003062:1009637	C>T	EX	TCA>TTA	S>L	aldolase	nonsyn
Agt|4	NC_003062:1012593	A>G	EX	GAC>GGC	D>G	outer	nonsyn
Agt|27	NC_003063:1023951	T>A	EX	GAG>GTG	E>V	replication	nonsyn
Agt|10	NC_003063:1026702	C>G	IG				
Agt|55	NC_003063:1031977	A>G	EX	ACT>ACC	T>T	hypothetical	syn
Agt|51	NC_003063:1034098	C>T	EX	GGC>GAC	G>D	urocanate	nonsyn
Agt|44	NC_003062:1034285	A>G	EX	GTA>GCA	V>A	hypothetical	nonsyn
Agt|2	NC_003063:1040330	C>T	EX	TCC>TTC	S>F	N-formimino-L-glutamate	nonsyn
Agt|14	NC_003063:1041515	T>C	IG				
Agt|45	NC_003062:1044622	G>A	EX	CAG>TAG	Q>*	hypothetical	nonsyn
Agt|30	NC_003062:1055422	C>A	EX	CGC>CTC	R>L	ABC	nonsyn
Agt|32	NC_003062:1074308	G>A	EX	GCC>ACC	A>T	hypothetical	nonsyn
Agt|5	NC_003063:1088232	C>T	IG				
Agt|32	NC_003062:1100114	C>T	EX	GCC>GCT	A>A	cytosol	syn
Agt|19	NC_003062:1120345	G>T	IG				
Agt|13	NC_003062:1120451	A>G	IG				
Agt|6	NC_003063:1131558	C>T	EX	ACG>ACA	T>T	two	syn
Agt|48	NC_003063:1140805	T>A	EX	CTG>CAG	L>Q	ferrichrome	nonsyn
Agt|4	NC_003063:1153224	C>T	EX	GGT>AGT	G>S	glucose	nonsyn
Agt|37	NC_003062:1175996	A>G	IG				
Agt|38	NC_003062:1183589	G>C	EX	GGT>GCT	G>A	hypothetical	nonsyn
Agt|21	NC_003062:1193274	C>T	EX	TTC>TTT	F>F	ABC	syn
Agt|34	NC_003063:1201024	G>C	EX	CGC>GGC	R>G	glutamine	nonsyn
Agt|38	NC_003063:1204722	C>G	EX	GCC>CCC	A>P	2-hydroxy-3-oxopropionate	nonsyn
Agt|39	NC_003062:1218555	G>C	IG				
Agt|36	NC_003063:1222225	C>T	EX	CCG>CTG	P>L	cytidylate	nonsyn
Agt|44	NC_003062:1224524	A>G	EX	GTG>GCG	V>A	polysaccharide	nonsyn
Agt|40	NC_003063:1232628	A>G	EX	AAC>AGC	N>S	dipeptide	nonsyn
Agt|2	NC_003063:1232815	C>G	EX	TAC>TAG	Y>*	dipeptide	nonsyn
Agt|30	NC_003062:1246470	G>T	EX	GCG>TCG	A>S	ATP-dependent	nonsyn
Agt|58	NC_003062:1250220	A>C	EX	GAT>GCT	D>A	ATP-dependent	nonsyn
Agt|28	NC_003062:1260599	C>G	EX	CTG>GTG	L>V	NADH	nonsyn
Agt|28	NC_003062:1266985	G>A	EX	GAA>AAA	E>K	NADH	nonsyn
Agt|26	NC_003062:1267561	C>A	EX	TTC>TTA	F>L	NADH	nonsyn

Agt|33	NC_003062:1267639	C>T	EX	AGC>AGT	S>S	NADH	syn
Agt|7	NC_003062:1271601	C>G	EX	ATC>ATG	I>M	NADH	nonsyn
Agt|10	NC_003062:1288825	C>A	IG				
Agt|22	NC_003063:1294976	G>A	EX	GAA>AAA	E>K	phosphoenolpyruvate-protein	nonsyn
Agt|6	NC_003063:1295987	C>T	EX	TCC>TTC	S>F	peptide	nonsyn
Agt|15	NC_003062:1303451	C>T	EX	GCC>GTC	A>V	acyl-CoA	nonsyn
Agt|9	NC_003063:1304022	C>A	EX	CTG>CTT	L>L	hypothetical	syn
Agt|10	NC_003063:1305815	T>C	IG				
Agt|15	NC_003063:1311363	T>G	EX	CAA>CCA	Q>P	dihydropicolinate	nonsyn
Agt|10	NC_003063:1314997	C>T	EX	CTG>TTG	L>L	oligopeptide	syn
Agt|2	NC_003062:1322817	C>G	EX	CGG>GGG	R>G	hypothetical	nonsyn
Agt|29	NC_003063:1324649	G>A	EX	CGC>TGC	R>C	methyltransferase	nonsyn
Agt|40	NC_003062:1329450	A>G	EX	GAA>GAG	E>E	penicillin-binding	syn
Agt|7	NC_003063:1339888	T>G	IG				
Agt|32	NC_003062:1345667	A>G	IG				
Agt|39	NC_003062:1349152	G>C	IG				
Agt|36	NC_003063:1352899	A>T	EX	GAA>GTA	E>V	amidophosphoribosyltransferase	nonsyn
Agt|2	NC_003063:1362161	A>G	IG				
Agt|27	NC_003062:1373663	C>G	EX	GCC>GCG	A>A	zinc	syn
Agt|7	NC_003062:1374946	A>G	EX	ATC>GTC	I>V	group	nonsyn
Agt|58	NC_003062:1375128	C>A	EX	GCC>GCA	A>A	group	syn
Agt|58	NC_003062:1375434	C>G	EX	AGC>AGG	S>R	group	nonsyn
Agt|38	NC_003062:1376269	C>G	EX	CGC>GGC	R>G	group	nonsyn
Agt|57	NC_003062:1382988	A>C	IG				
Agt|36	NC_003062:1411771	C>T	EX	GCG>GCA	A>A	long-chain-fatty-acid-CoA-ligase	syn
Agt|54	NC_003062:1416168	C>T	EX	CAG>TAG	Q>*	transcriptional	nonsyn
Agt|57	NC_003062:1423725	A>G	IG				
Agt|47	NC_003062:1426455	G>A	IG				
Agt|51	NC_003062:1426480	G>A	IG				
Agt|25	NC_003062:1438309	T>A	EX	CAT>CAA	H>Q	two	nonsyn
Agt|25	NC_003062:1438310	C>T	EX	CTT>TTT	L>F	two	nonsyn
Agt|6	NC_003062:1439200	C>T	EX	GAC>GAT	D>D	two	syn
Agt|14	NC_003062:1443034	G>A	EX	GGG>GGA	G>G	two	syn
Agt|46	NC_003062:1446204	G>C	EX	CTG>CTC	L>L	putative	syn
Agt|54	NC_003063:1459767	C>T	EX	GCT>ACT	A>T	serine/threonine	nonsyn
Agt|13	NC_003062:1465326	G>C	EX	GGT>GCT	G>A	Camphor	nonsyn
Agt|29	NC_003063:1467791	T>C	EX	GAA>GGA	E>G	forkhead-type	nonsyn
Agt|10	NC_003063:1480640	C>T	EX	TCA>TTA	S>L	hypothetical	nonsyn
Agt|53	NC_003062:1488422	C>A	EX	CCG>CCT	P>P	thymydilate	syn
Agt|15	NC_003062:1490675	A>C	EX	GTT>GTG	V>V	rare	syn
Agt|2	NC_003062:1491913	G>A	IG				
Agt|43	NC_003062:1496248	T>C	EX	ACC>GCC	T>A	DNA	nonsyn
Agt|15	NC_003063:1505626	C>T	IG				
Agt|25	NC_003063:1509227	C>G	EX	CTG>GTG	L>V	sugar	nonsyn
Agt|28	NC_003063:1509365	C>G	EX	GGC>GGG	G>G	ribose	syn
Agt|40	NC_003063:1529182	C>T	EX	GCG>GTG	A>V	denitrification	nonsyn
Agt|57	NC_003062:1537053	G>A	EX	GCG>GTG	A>V	magnesium	nonsyn
Agt|26	NC_003062:1540418	G>A	EX	CGC>CAC	R>H	formate	nonsyn
Agt|17	NC_003062:1543348	C>A	IG				
Agt|33	NC_003062:1543350	C>A	IG				
Agt|30	NC_003063:1543889	A>T	EX	ACC>TCC	T>S	nitrate	nonsyn
Agt|37	NC_003063:1555080	G>T	EX	GCG>TCG	A>S	bacteriocin	nonsyn
Agt|59	NC_003062:1555269	C>T	EX	GCG>ACG	A>T	transcriptional	nonsyn
Agt|44	NC_003063:1555761	G>C	EX	GTC>CTC	V>L	bacteriocin	nonsyn
Agt|59	NC_003063:1557276	C>G	EX	GAA>CAA	E>Q	hypothetical	nonsyn
Agt|33	NC_003063:1570366	C>A	IG				
Agt|40	NC_003062:1576477	A>G	EX	TTC>TCC	F>S	lipid	nonsyn
Agt|28	NC_003062:1576623	C>A	EX	AAG>AAT	K>N	lipid	nonsyn
Agt|55	NC_003062:1580343	G>A	EX	AAC>AAT	N>N	3-oxoacyl-(acyl-carrier-protein)	syn

Agt|6	NC_003062:1585605	T>A	EX	CTT>CTA	L>L	poly-beta-hydroxybutyrate	syn
Agt|14	NC_003063:1588294	G>C	EX	GGC>CGC	G>R	hippurate	nonsyn
Agt|10	NC_003063:1592428	C>A	EX	AAC>AAA	N>K	sorbitol	nonsyn
Agt|57	NC_003062:1604819	C>A	IG				
Agt|32	NC_003062:1607432	G>A	EX	GAA>AAA	E>K	DNA	nonsyn
Agt|42	NC_003063:1613547	A>G	IG				
Agt|51	NC_003063:1624568	C>T	EX	ATC>ATT	I>I	MFS	syn
Agt|25	NC_003063:1625784	A>G	IG				
Agt|16	NC_003062:1628852	T>G	EX	GAG>GCG	E>A	ABC	nonsyn
Agt|9	NC_003062:1639773	C>T	EX	GCG>ACG	A>T	nitroreductase	nonsyn
Agt|46	NC_003063:1641991	A>G	IG				
Agt|43	NC_003062:1648053	C>A	EX	ATC>ATA	I>I	soluble	syn
Agt|4	NC_003062:1652748	G>A	IG				
Agt|36	NC_003063:1652772	C>T	EX	TTC>TTT	F>F	hypothetical	syn
Agt|24	NC_003063:1653511	T>C	EX	TTC>TCC	F>S	dehydrogenase	nonsyn
Agt|7	NC_003062:1657276	G>C	EX	GCG>CCG	A>P	hypothetical	nonsyn
Agt|21	NC_003063:1659543	G>A	EX	CGC>CGT	R>R	myo-inositol	syn
Agt|32	NC_003063:1667916	C>T	EX	GAG>GAA	E>E	AraC	syn
Agt|14	NC_003062:1673649	G>C	EX	GCC>CCC	A>P	molybdenum	nonsyn
Agt|29	NC_003062:1674138	G>T	EX	GAT>TAT	D>Y	molybdopterin	nonsyn
Agt|29	NC_003063:1680599	C>A	EX	GAG>TAG	E>*	amino	nonsyn
Agt|26	NC_003063:1689054	C>G	EX	CGC>CGG	R>R	LysR	syn
Agt|27	NC_003062:1694602	G>T	EX	CGC>AGC	R>S	hypothetical	nonsyn
Agt|34	NC_003063:1707810	C>T	EX	TCG>TCA	S>S	hypothetical	syn
Agt|42	NC_003062:1711026	C>A	IG				
Agt|55	NC_003063:1711334	C>T	IG				
Agt|13	NC_003062:1711797	C>T	IG				
Agt|22	NC_003062:1722946	G>T	EX	GCC>GCA	A>A	manganese	syn
Agt|42	NC_003063:1723918	C>A	IG				
Agt|33	NC_003063:1725329	A>G	IG				
Agt|17	NC_003062:1725860	G>A	IG				
Agt|33	NC_003062:1738886	G>A	EX	CTC>TTC	L>F	hypothetical	nonsyn
Agt|2	NC_003062:1750169	A>G	IG				
Agt|6	NC_003062:1750595	C>G	EX	CGC>GGC	R>G	hypothetical	nonsyn
Agt|6	NC_003062:1753540	C>T	IG				
Agt|53	NC_003063:1758474	G>C	EX	GCC>GCG	A>A	transposase	syn
Agt|28	NC_003062:1762701	G>C	EX	GCC>GCG	A>A	transcription-repair	syn
Agt|43	NC_003062:1766369	G>C	IG				
Agt|40	NC_003063:1771819	T>G	IG				
Agt|29	NC_003063:1773593	C>T	EX	GCA>GTA	A>V	mannonate	nonsyn
Agt|47	NC_003062:1776367	C>A	EX	ATG>ATT	M>I	lipoprotein	nonsyn
Agt|32	NC_003062:1785869	C>T	EX	GCT>ACT	A>T	hypothetical	nonsyn
Agt|40	NC_003062:1792868	A>C	EX	CAA>CAC	Q>H	hypothetical	nonsyn
Agt|32	NC_003062:1797103	C>T	EX	CTT>TTT	L>F	hypothetical	nonsyn
Agt|17	NC_003063:1808391	C>A	EX	GAA>TAA	E>*	sugar	nonsyn
Agt|39	NC_003062:1818469	T>C	IG				
Agt|20	NC_003062:1822170	G>A	EX	ACG>ACA	T>T	hypothetical	syn
Agt|41	NC_003062:1826965	G>T	EX	CGC>CTC	R>L	hypothetical	nonsyn
Agt|5	NC_003063:1828472	C>G	EX	ACG>AGG	T>R	ABC	nonsyn
Agt|9	NC_003063:1831441	C>T	IG				
Agt|32	NC_003063:1833023	A>C	EX	ACT>CCT	T>P	cell	nonsyn
Agt|33	NC_003063:1834432	C>T	IG				
Agt|29	NC_003062:1836785	A>T	EX	CTC>CAC	L>H	inositol	nonsyn
Agt|16	NC_003062:1837357	G>C	EX	TCC>TCG	S>S	inositol	syn
Agt|48	NC_003063:1848598	C>A	EX	CTC>CTA	L>L	amino	syn
Agt|10	NC_003063:1851314	A>C	EX	TAT>TCT	Y>S	diguanylate	nonsyn
Agt|24	NC_003062:1884026	T>A	EX	ATC>TTC	I>F	ABC	nonsyn
Agt|6	NC_003062:1897405	C>T	IG				
Agt|39	NC_003062:1900502	C>T	EX	GCC>GTC	A>V	hypothetical	nonsyn

Agt|51	NC_003062:1902061	C>T	EX	ACG>ACA	T>T	DNA-directed	syn
Agt|42	NC_003062:1903363	T>C	IG				
Agt|14	NC_003063:1905218	T>G	IG				
Agt|28	NC_003062:1910068	G>A	EX	GGC>GGT	G>G	50S	syn
Agt|10	NC_003062:1911466	C>G	EX	GAG>CAG	E>Q	30S	nonsyn
Agt|29	NC_003062:1915392	G>A	IG				
Agt|42	NC_003063:1920950	C>G	EX	CCC>GCC	P>A	hypothetical	nonsyn
Agt|30	NC_003062:1924559	T>C	EX	ACC>GCC	T>A	DNA-directed	nonsyn
Agt|47	NC_003062:1933329	C>A	EX	GAC>TAC	D>Y	50S	nonsyn
Agt|22	NC_003062:1933880	G>A	EX	CCG>TCG	P>S	50S	nonsyn
Agt|47	NC_003063:1934849	C>T	EX	GCC>GTC	A>V	hypothetical	nonsyn
Agt|10	NC_003062:1935030	C>G	IG				
Agt|13	NC_003062:1935797	T>C	EX	GAT>GGT	D>G	UDP-glucose	nonsyn
Agt|44	NC_003063:1936259	C>G	EX	CGG>CCG	R>P	hypothetical	nonsyn
Agt|10	NC_003062:1941946	G>A	EX	CTG>TTG	L>L	cyclopropane-fatty-acyl-phospholipid	syn
Agt|32	NC_003063:1976371	A>T	EX	CCT>CCA	P>P	hypothetical	syn
Agt|38	NC_003062:1988944	G>A	EX	GAC>GAT	D>D	hypothetical	syn
Agt|42	NC_003062:1990792	G>C	EX	CTG>CTC	L>L	hypothetical	syn
Agt|58	NC_003063:1991393	C>T	EX	CCT>TCT	P>S	UDP-phosphate	nonsyn
Agt|13	NC_003063:1993247	G>T	EX	GCG>TCG	A>S	hypothetical	nonsyn
Agt|21	NC_003062:1994765	G>A	EX	CGC>TGC	R>C	acetolactate	nonsyn
Agt|4	NC_003063:2004467	A>T	EX	ATC>AAC	I>N	IclR	nonsyn
Agt|7	NC_003062:2021475	G>C	EX	ATG>ATC	M>I	DNA	nonsyn
Agt|11	NC_003062:2023284	G>A	IG				
Agt|29	NC_003063:2033942	G>T	EX	ATC>ATA	I>I	hypothetical	syn
Agt|11	NC_003063:2034898	G>T	EX	CTC>CTA	L>L	hypothetical	syn
Agt|41	NC_003062:2046583	G>C	EX	GCC>GGC	A>G	UDP-3-0-(3-hydroxymyristoyl)	nonsyn
Agt|26	NC_003063:2046593	C>T	IG				
Agt|57	NC_003062:2047509	T>C	EX	GCA>GCG	A>A	cell	syn
Agt|11	NC_003062:2049241	G>T	EX	CGC>AGC	R>S	cell	nonsyn
Agt|11	NC_003062:2049246	C>T	EX	GGC>GAC	G>D	cell	nonsyn
Agt|36	NC_003063:2052723	A>C	EX	CTC>CGC	L>R	oxidoreductase	nonsyn
Agt|28	NC_003063:2054152	A>G	EX	CTG>CCG	L>P	NADP-dependent	nonsyn
Agt|43	NC_003062:2060951	G>A	EX	TCC>TCT	S>S	UDP-N-acetylmuramoyl-L-alanyl-D-glutama	syn
Agt|36	NC_003062:2080737	C>A	EX	CTG>ATG	L>M	hypothetical	nonsyn
Agt|5	NC_003062:2087307	G>A	IG				
Agt|38	NC_003062:2096409	G>A	EX	AGC>AGT	S>S	hypothetical	syn
Agt|15	NC_003062:2098874	C>A	EX	CGC>CGA	R>R	hypothetical	syn
Agt|7	NC_003062:2102147	T>G	EX	AAA>CAA	K>Q	chromosome	nonsyn
Agt|48	NC_003062:2109543	C>A	IG				
Agt|5	NC_003062:2110339	C>T	IG				
Agt|42	NC_003062:2134060	T>A	EX	GAG>GTG	E>V	hypothetical	nonsyn
Agt|51	NC_003062:2157870	A>G	EX	GAT>GAC	D>D	hypothetical	syn
Agt|42	NC_003062:2158585	A>C	IG				
Agt|44	NC_003062:2163412	T>C	IG				
Agt|40	NC_003062:2163600	G>C	IG				
Agt|33	NC_003062:2176939	A>G	EX	ACG>GCG	T>A	two	nonsyn
Agt|39	NC_003062:2202170	G>A	EX	GTG>GTA	V>V	ABC	syn
Agt|43	NC_003062:2216799	C>G	EX	CGG>CCG	R>P	hypothetical	nonsyn
Agt|42	NC_003062:2221661	G>A	EX	TCA>TTA	S>L	poly(A)	nonsyn
Agt|34	NC_003062:2225834	C>T	EX	CCG>TCG	P>S	hypothetical	nonsyn
Agt|6	NC_003062:2233382	G>A	EX	CCC>TCC	P>S	acetyltransferase	nonsyn
Agt|10	NC_003062:2274061	C>A	IG				
Agt|13	NC_003062:2276897	C>T	EX	GCC>ACC	A>T	hypothetical	nonsyn
Agt|30	NC_003062:2278523	G>A	EX	GAA>AAA	E>K	hypothetical	nonsyn
Agt|26	NC_003062:2339410	A>C	EX	GTC>GGC	V>G	ABC	nonsyn
Agt|59	NC_003062:2343265	G>T	EX	GAT>TAT	D>Y	Hypothetical	nonsyn
Agt|2	NC_003062:2405531	G>A	EX	CTG>CTA	L>L	two	syn
Agt|17	NC_003062:2416269	T>C	IG				

Agt|19	NC_003062:2420743	G>A	EX	TCG>TCA	S>S	hypothetical	syn	
Agt|37	NC_003062:2422428	G>A	IG					
Agt|15	NC_003062:2446288	T>C	IG					
Agt|20	NC_003062:2449521	G>A	EX	GGC>AGC	G>S	HlyD	nonsyn	
Agt|42	NC_003062:2465510	C>G	EX	GTC>GTG	V>V	NAD(P)+	syn	
Agt|55	NC_003062:2488439	G>A	EX	TGG>TAG	W>*	dipeptidase	nonsyn	
Agt|37	NC_003062:2512099	C>T	EX	CTT>TTT	L>F	N-methylproline	nonsyn	
Agt|58	NC_003062:2534359	G>A	EX	GAA>AAA	E>K	ABC	nonsyn	
Agt|36	NC_003062:2540528	C>T	EX	TGG>TGA	W>*	thiamine	nonsyn	
Agt|28	NC_003062:2580720	T>G	EX	ATG>CTG	M>L	ABC	nonsyn	
Agt|4	NC_003062:2606662	C>T	EX	GAG>AAG	E>K	ATP	nonsyn	
Agt|16	NC_003062:2608924	G>A	EX	CGC>TGC	R>C	ATP	nonsyn	
Agt|24	NC_003062:2643419	C>T	EX	CGA>TGA	R>*	hypothetical	nonsyn	
Agt|21	NC_003062:2666987	C>T	IG					
Agt|43	NC_003062:2696055	T>C	EX	GAA>GGA	E>G	hypothetical	nonsyn	
Agt|44	NC_003062:2701803	C>T	EX	CCG>CCA	P>P	ABC	syn	
Agt|17	NC_003062:2734240	C>T	EX	GCA>ACA	A>T	oxidoredutase	nonsyn	
Agt|47	NC_003062:2737023	T>C	EX	ATA>ACA	I>T	oxidoreductase	nonsyn	
Agt|41	NC_003062:2739373	C>T	EX	ATC>ATT	I>I	hypothetical	syn	
Agt|9	NC_003062:2766294	G>C	IG					
Agt|51	NC_003062:2794483	G>C	EX	GGC>GGG	G>G	hypothetical	syn	
Agt|24	NC_003062:2802078	C>T	EX	GCT>ACT	A>T	precorrin-3B	nonsyn	
Agt|16	NC_003062:2802228	T>C	EX	TGA>TGG	*>W	precorrin-2	nonsyn	
Agt|10	NC_003062:2820870	C>T	EX	CAG>CAA	Q>Q	ABC	syn	
Agt|10	NC_003062:2823898	G>A	IG					
Agt|59	NC_003062:2825078	G>A	EX	GGC>AGC	G>S	D-galactarate	nonsyn	
Agt|25	NC_003062:2837494	C>G	EX	CGG>CCG	R>P	tRNA	nonsyn	
Bs|65	NZ_CM000488:1807	T>C	IG					
Bs|65	NZ_CM000488:1810	G>A	IG					
Bs|57	NZ_CM000488:2854	T>C	EX	TCG>CCG	S>P	DNA	nonsyn	
Bs|3	NZ_CM000488:3701	A>C	EX	AAA>CAA	K>Q	recombination	nonsyn	seq
Bs|54	NZ_CM000488:4731	G>A	IG					seq
Bs|69	plasmid:18786	G>A	IG					
Bs|52	plasmid:37642	T>A	IG					
Bs|68	NZ_CM000488:52841	C>T	EX	GGC>GGT	G>G	hypothetical	syn	
Bs|24	NZ_CM000488:67598	A>G	EX	GAA>GGA	E>G	hypothetical	nonsyn	
Bs|32	plasmid:71964	G>T	IG					
Bs|40	NZ_CM000488:75323	A>G	EX	ACA>GCA	T>A	hypothetical	nonsyn	
Bs|71	NZ_CM000488:75492	G>A	EX	AGC>AAC	S>N	hypothetical	nonsyn	
Bs|2	plasmid:77327	A>C	IG					
Bs|69	NZ_CM000488:109377	T>C	EX	GTT>GCT	V>A	hypothetical	nonsyn	
Bs|8	NZ_CM000488:120051	G>C	IG					
Bs|55	NZ_CM000488:120607	C>T	EX	GCT>GTT	A>V	50S	nonsyn	seq
Bs|30	NZ_CM000488:143224	C>T	EX	GCT>GTT	A>V	50S	nonsyn	
Bs|8	NZ_CM000488:254839	T>C	IG					
Bs|17	NZ_CM000488:295122	T>G	EX	TAT>TCT	Y>S	hypothetical	nonsyn	seq
Bs|40	NZ_CM000488:315244	C>T	EX	CAG>TAG	Q>*	hypothetical	nonsyn	seq
Bs|24	NZ_CM000488:319694	C>T	EX	CGG>CGA	R>R	hypothetical	syn	
Bs|48	NZ_CM000488:340482	C>T	EX	GCG>ACG	A>T	hypothetical	nonsyn	
Bs|63	NZ_CM000488:435548	C>T	IG					
Bs|45	NZ_CM000488:442405	C>T	EX	GCG>GTG	A>V	4-aminobutyrate	nonsyn	
Bs|43	NZ_CM000488:479685	A>G	EX	ATC>GTC	I>V	hypothetical	nonsyn	
Bs|11	NZ_CM000488:486200	C>T	EX	CAA>TAA	Q>*	hypothetical	nonsyn	
Bs|48	NZ_CM000488:498298	C>A	EX	AAC>AAA	N>K	two-component	nonsyn	
Bs|45	NZ_CM000488:563093	T>C	IG					
Bs|45	NZ_CM000488:572257	A>G	EX	ATA>GTA	I>V	hypothetical	nonsyn	
Bs|45	NZ_CM000488:582286	T>C	EX	TAC>TGC	Y>C	hypothetical	nonsyn	
Bs|31	NZ_CM000488:600726	A>G	EX	ATT>GTT	I>V	hypothetical	nonsyn	
Bs|41	NZ_CM000488:604739	C>A	EX	GGG>TGG	G>W	ATP-binding	nonsyn	

Bs|1	NZ_CM000488:625853	A>G	IG					seq
Bs|30	NZ_CM000488:634373	G>A	IG					
Bs|40	NZ_CM000488:634589	A>G	IG					
Bs|71	NZ_CM000488:646830	C>T	EX	TTC>TTT	F>F	redox-sensing	syn	
Bs|38	NZ_CM000488:668109	A>G	EX	GAA>GGA	E>G	H+-symporter	nonsyn	
Bs|44	NZ_CM000488:680633	A>G	IG					
Bs|45	NZ_CM000488:696305	G>A	IG					
Bs|7	NZ_CM000488:706589	A>G	EX	TAT>TGT	Y>C	phosphoribosylaminoimidazole	nonsyn	
Bs|24	NZ_CM000488:709521	T>A	EX	CTC>CAC	L>H	phosphoribosylamine--glycine	nonsyn	seq
Bs|62	NZ_CM000488:710269	G>A	EX	ATG>ATA	M>I	phosphoribosylamine--glycine	nonsyn	
Bs|15	NZ_CM000488:715395	T>G	EX	TAT>TAG	Y>*	hypothetical	nonsyn	
Bs|17	NZ_CM000488:741098	C>T	EX	GCT>GTT	A>V	hypothetical	nonsyn	
Bs|36	NZ_CM000488:766349	G>A	EX	GGA>AGA	G>R	hypothetical	nonsyn	
Bs|45	NZ_CM000488:772453	T>C	EX	GTC>GCC	V>A	hypothetical	nonsyn	
Bs|8	NZ_CM000488:786981	G>T	IG					
Bs|45	NZ_CM000488:800565	A>T	EX	GAA>GTA	E>V	hypothetical	nonsyn	
Bs|30	NZ_CM000488:806166	T>C	IG					
Bs|24	NZ_CM000488:806593	C>T	EX	TTC>TTT	F>F	hypothetical	syn	
Bs|19	NZ_CM000488:849567	G>A	IG					
Bs|44	NZ_CM000488:851409	C>T	EX	CTA>TTA	L>L	trehalose-6-phosphate	syn	
Bs|60	NZ_CM000488:858089	C>T	EX	GAA>AAA	E>K	bifunctional	nonsyn	
Bs|45	NZ_CM000488:859822	T>C	EX	ACA>GCA	T>A	hypothetical	nonsyn	
Bs|62	NZ_CM000488:905413	A>G	EX	AAA>GAA	K>E	hypothetical	nonsyn	
Bs|69	NZ_CM000488:923045	A>G	EX	GAG>GGG	E>G	hypothetical	nonsyn	
Bs|69	NZ_CM000488:927180	G>A	EX	GCA>ACA	A>T	hypothetical	nonsyn	
Bs|45	NZ_CM000488:945566	A>G	EX	CAC>CGC	H>R	hypothetical	nonsyn	
Bs|55	NZ_CM000488:951042	T>C	IG					
Bs|41	NZ_CM000488:958925	C>A	EX	CGC>CTC	R>L	vegetative	nonsyn	
Bs|63	NZ_CM000488:970888	C>T	EX	GCA>GTA	A>V	hypothetical	nonsyn	
Bs|68	NZ_CM000488:980449	C>T	EX	TCT>TTT	S>F	hypothetical	nonsyn	
Bs|40	NZ_CM000488:996728	T>A	EX	CTG>CAG	L>Q	hypothetical	nonsyn	
Bs|36	NZ_CM000488:1005692	C>T	EX	CGC>CGT	R>R	glycerol-3-phosphate	syn	
Bs|68	NZ_CM000488:1018011	G>A	IG					
Bs|3	NZ_CM000488:1020218	C>G	EX	ATG>ATC	M>I	LysR	nonsyn	seq
Bs|65	NZ_CM000488:1036955	C>A	EX	GTT>TTT	V>F	camphor	nonsyn	
Bs|57	NZ_CM000488:1040572	T>C	EX	CAC>CGC	H>R	hypothetical	nonsyn	
Bs|19	NZ_CM000488:1065041	A>T	EX	AAA>AAT	K>N	hypothetical	nonsyn	seq
Bs|35	NZ_CM000488:1069888	A>G	EX	GAT>GAC	D>D	molecular	syn	
Bs|2	NZ_CM000488:1084215	T>C	EX	GCT>GCC	A>A	penicillin-binding	syn	
Bs|8	NZ_CM000488:1089001	A>G	IG					
Bs|4	NZ_CM000488:1100489	A>C	EX	ATC>CTC	I>L	acyl-CoA	nonsyn	seq
Bs|22	NZ_CM000488:1105144	G>A	IG					
Bs|36	NZ_CM000488:1105183	C>T	IG					
Bs|37	NZ_CM000488:1118207	A>G	EX	AAG>AGG	K>R	hypothetical	nonsyn	
Bs|7	NZ_CM000488:1143110	T>C	EX	CCT>CCC	P>P	exonuclease	syn	seq
Bs|45	NZ_CM000488:1198656	C>T	EX	CAT>TAT	H>Y	carbamoyl	nonsyn	
Bs|46	NZ_CM000488:1219615	C>T	EX	GCT>GTT	A>V	oligopeptide	nonsyn	
Bs|62	NZ_CM000488:1224266	G>A	EX	GGC>GAC	G>D	oligopeptide	nonsyn	
Bs|32	NZ_CM000488:1252649	G>A	IG					
Bs|17	NZ_CM000488:1257260	C>T	EX	AGG>AAG	R>K	hypothetical	nonsyn	
Bs|15	NZ_CM000488:1279138	A>C	EX	TGG>GGG	W>G	heme	nonsyn	seq
Bs|44	NZ_CM000488:1279814	A>G	IG					
Bs|50	NZ_CM000488:1282818	C>T	EX	CGG>TGG	R>W	hypothetical	nonsyn	
Bs|54	NZ_CM000488:1285484	G>T	EX	ATG>ATT	M>I	hypothetical	nonsyn	
Bs|31	NZ_CM000488:1293747	A>G	EX	ACA>GCA	T>A	uroporphyrinogen-III	nonsyn	
Bs|55	NZ_CM000488:1295550	A>G	EX	ATA>ACA	I>T	hypothetical	nonsyn	
Bs|57	NZ_CM000488:1308713	T>C	EX	AGT>AGC	S>S	LacI	syn	
Bs|45	NZ_CM000488:1317936	C>T	EX	GCC>ACC	A>T	hypothetical	nonsyn	
Bs|57	NZ_CM000488:1320449	T>C	IG					seq

Bs|1	NZ_CM000488:1327052	T>A	EX	TTT>TAT	F>Y	hypothetical	nonsyn	seq
Bs|26	NZ_CM000488:1328269	A>G	EX	GGA>GGG	G>G	hypothetical	syn	
Bs|15	NZ_CM000488:1334273	G>A	IG					
Bs|28	NZ_CM000488:1351919	C>T	EX	GCC>ACC	A>T	hypothetical	nonsyn	
Bs|14	NZ_CM000488:1365167	G>T	IG					
Bs|32	NZ_CM000488:1386408	G>T	IG					
Bs|17	NZ_CM000488:1394780	A>C	EX	TAA>TCA	*>S	hypothetical	nonsyn	
Bs|68	NZ_CM000488:1395164	G>A	EX	TCG>TCA	S>S	hypothetical	syn	
Bs|38	NZ_CM000488:1409406	C>T	EX	GCC>GTC	A>V	hypothetical	nonsyn	seq
Bs|53	NZ_CM000488:1409851	A>G	IG					
Bs|22	NZ_CM000488:1410578	C>T	EX	GTC>GTT	V>V	hypothetical	syn	
Bs|7	NZ_CM000488:1418709	T>C	EX	TCT>CCT	S>P	two-component	nonsyn	
Bs|63	NZ_CM000488:1461920	G>A	EX	GAA>AAA	E>K	spore	nonsyn	
Bs|2	NZ_CM000488:1499082	A>G	IG					seq
Bs|45	NZ_CM000488:1502040	T>A	EX	TAT>AAT	Y>N	hypothetical	nonsyn	seq
Bs|68	NZ_CM000488:1509006	A>T	EX	GAA>GTA	E>V	phosphotransferase	nonsyn	
Bs|35	NZ_CM000488:1511575	A>G	IG					
Bs|24	NZ_CM000488:1538980	T>C	IG					
Bs|46	NZ_CM000488:1574667	C>T	EX	GCG>GTG	A>V	hypothetical	nonsyn	
Bs|54	NZ_CM000488:1576391	G>A	EX	GAC>GAT	D>D	hypothetical	syn	
Bs|52	NZ_CM000488:1599437	C>A	EX	GGC>GGA	G>G	bacillopeptidase	syn	
Bs|27	NZ_CM000488:1619202	G>C	EX	GTG>GTC	V>V	uracil	syn	
Bs|51	NZ_CM000488:1630251	G>C	EX	GGC>GCC	G>A	phosphoadenosine	nonsyn	
Bs|51	NZ_CM000488:1630255	T>A	EX	TGT>TGA	C>*	phosphoadenosine	nonsyn	
Bs|51	NZ_CM000488:1630266	C>A	EX	ACT>AAT	T>N	phosphoadenosine	nonsyn	
Bs|51	NZ_CM000488:1630267	T>A	EX	ACT>ACA	T>T	phosphoadenosine	syn	
Bs|7	NZ_CM000488:1645316	C>T	EX	CAT>TAT	H>Y	primosome	nonsyn	
Bs|15	NZ_CM000488:1646988	A>G	EX	AAC>AGC	N>S	methionyl-tRNA	nonsyn	seq
Bs|43	NZ_CM000488:1670591	A>G	EX	GTT>GCT	V>A	hypothetical	nonsyn	
Bs|27	NZ_CM000488:1681434	G>A	EX	GGA>AGA	G>R	succinyl-CoA	nonsyn	
Bs|15	NZ_CM000488:1682464	A>C	EX	CAA>CCA	Q>P	DNA	nonsyn	
Bs|1	NZ_CM000488:1692793	A>T	EX	AGC>TGC	S>C	flagellar	nonsyn	seq
Bs|54	NZ_CM000488:1713940	C>T	EX	ACT>ATT	T>I	two-component	nonsyn	
Bs|69	NZ_CM000488:1717754	A>G	EX	GTA>GTG	V>V	30S	syn	
Bs|19	NZ_CM000488:1728130	A>G	EX	AAA>GAA	K>E	DNA	nonsyn	
Bs|17	NZ_CM000488:1752456	A>G	EX	ACA>GCA	T>A	DNA	nonsyn	
Bs|40	NZ_CM000488:1759170	G>T	EX	GGG>GGT	G>G	3-ketoacyl-(acyl-carrier-protein)	syn	
Bs|46	NZ_CM000488:1763811	T>G	IG					seq
Bs|40	NZ_CM000488:1774095	T>C	IG					
Bs|60	NZ_CM000488:1781664	T>C	EX	GTG>GCG	V>A	transcriptional	nonsyn	seq
Bs|48	NZ_CM000488:1784118	C>T	IG					
Bs|49	NZ_CM000488:1788828	C>T	EX	CAT>TAT	H>Y	polyketide	nonsyn	
Bs|54	NZ_CM000488:1796764	A>G	EX	ACA>GCA	T>A	polyketide	nonsyn	
Bs|41	NZ_CM000488:1804735	C>T	EX	CAA>TAA	Q>*	polyketide	nonsyn	
Bs|49	NZ_CM000488:1816086	T>G	EX	GTC>GGC	V>G	polyketide	nonsyn	
Bs|17	NZ_CM000488:1833150	T>C	EX	GTT>GCT	V>A	polyketide	nonsyn	
Bs|20	NZ_CM000488:1856383	A>G	EX	CCA>CCG	P>P	polyketide	syn	
Bs|36	NZ_CM000488:1864188	C>T	EX	GCT>ACT	A>T	multidrug	nonsyn	
Bs|48	NZ_CM000488:1864847	C>T	EX	GGG>AGG	G>R	hypothetical	nonsyn	
Bs|13	NZ_CM000488:1875641	A>G	IG					
Bs|3	NZ_CM000488:1887763	A>T	EX	TTA>TTT	L>F	H+-symporter	nonsyn	seq
Bs|3	NZ_CM000488:1900124	C>T	IG					seq
Bs|31	NZ_CM000488:1901359	C>T	IG					
Bs|1	NZ_CM000488:1904003	C>A	IG					seq
Bs|54	NZ_CM000488:1915494	A>G	EX	TCT>CCT	S>P	hypothetical	nonsyn	
Bs|24	NZ_CM000488:1915729	A>G	IG					seq
Bs|37	NZ_CM000488:1919396	G>C	EX	GGA>GCA	G>A	transketolase	nonsyn	
Bs|1	NZ_CM000488:1925714	T>A	IG					seq
Bs|49	NZ_CM000488:1930701	G>A	IG					

Bs|37	NZ_CM000488:1934289	G>A	EX	GGG>AGG	G>R	DNA	nonsyn	seq
Bs|2	NZ_CM000488:1936697	T>G	EX	GAT>GAG	D>E	DNA	nonsyn	seq
Bs|55	NZ_CM000488:1945157	C>G	IG					
Bs|45	NZ_CM000488:1947170	T>C	EX	TTC>TCC	F>S	hypothetical	nonsyn	
Bs|30	NZ_CM000488:1954548	G>C	EX	CCC>CCG	P>P	acyl-CoA	syn	
Bs|55	NZ_CM000488:1955568	C>A	EX	GCA>TCA	A>S	hypothetical	nonsyn	seq
Bs|8	NZ_CM000488:1964164	C>T	EX	CCG>CCA	P>P	plipastatin	syn	
Bs|74	NZ_CM000488:1977750	G>A	EX	CTT>TTT	L>F	plipastatin	nonsyn	
Bs|57	NZ_CM000488:1983591	A>G	EX	ATA>ACA	I>T	plipastatin	nonsyn	
Bs|63	NZ_CM000488:1992969	A>G	EX	ATC>ACC	I>T	plipastatin	nonsyn	
Bs|2	NZ_CM000488:1997445	T>C	IG					seq
Bs|32	NZ_CM000488:1998593	C>T	EX	GAT>AAT	D>N	penicillin-binding	nonsyn	
Bs|49	NZ_CM000488:2003425	T>C	IG					
Bs|54	NZ_CM000488:2009251	T>C	EX	CAG>CGG	Q>R	glutamate	nonsyn	
Bs|4	NZ_CM000488:2015305	C>T	EX	GCT>ACT	A>T	gamma-glutamyl	nonsyn	seq
Bs|46	NZ_CM000488:2035993	C>T	IG					
Bs|55	NZ_CM000488:2036065	C>T	IG					seq
Bs|69	NZ_CM000488:2050660	G>A	EX	GTC>GTT	V>V	phosphoenolpyruvate	syn	seq
Bs|32	NZ_CM000488:2054393	A>C	EX	TTA>TGA	L>*	endo-1,4-beta-xylanase	nonsyn	seq
Bs|57	NZ_CM000488:2054488	A>G	IG					
Bs|14	NZ_CM000488:2058851	T>C	IG					
Bs|65	NZ_CM000488:2081564	A>T	EX	TAT>AAT	Y>N	hypothetical	nonsyn	
Bs|54	NZ_CM000488:2098288	A>T	EX	ATC>TTC	I>F	hypothetical	nonsyn	
Bs|22	NZ_CM000488:2103353	G>A	EX	GCT>ACT	A>T	superoxide	nonsyn	
Bs|55	NZ_CM000488:2104440	G>A	EX	CGA>TGA	R>*	hypothetical	nonsyn	
Bs|57	NZ_CM000488:2133034	T>G	EX	GCT>GCG	A>A	hypothetical	syn	
Bs|57	NZ_CM000488:2134117	G>A	EX	GCT>GTT	A>V	hypothetical	nonsyn	
Bs|14	NZ_CM000488:2141260	A>G	EX	TGG>CGG	W>R	acetylornithine	nonsyn	
Bs|15	NZ_CM000488:2143032	T>C	EX	GCA>GCG	A>A	hypothetical	syn	
Bs|14	NZ_CM000488:2151223	A>G	IG					seq
Bs|40	NZ_CM000488:2174720	A>G	IG					
Bs|22	NZ_CM000488:2210135	G>A	EX	ACC>ATC	T>I	hypothetical	nonsyn	
Bs|38	NZ_CM000488:2224366	T>A	IG					
Bs|2	NZ_CM000488:2224432	C>T	IG					seq
Bs|37	NZ_CM000488:2243177	C>T	EX	GCC>GTC	A>V	hypothetical	nonsyn	
Bs|65	NZ_CM000488:2246794	C>T	IG					
Bs|24	NZ_CM000488:2250659	C>T	EX	AGC>AGT	S>S	hypothetical	syn	seq
Bs|45	NZ_CM000488:2252368	A>G	EX	CAG>CGG	Q>R	hypothetical	nonsyn	
Bs|54	NZ_CM000488:2256497	G>A	EX	TGG>TAG	W>*	hypothetical	nonsyn	
Bs|65	NZ_CM000488:2276623	T>C	IG					
Bs|65	NZ_CM000488:2303696	T>C	EX	GAA>GGA	E>G	hypothetical	nonsyn	
Bs|31	NZ_CM000488:2316172	G>A	EX	CCG>CTG	P>L	naringenin-chalcone	nonsyn	
Bs|6	NZ_CM000488:2364666	A>G	EX	TTA>TCA	L>S	hypothetical	nonsyn	seq
Bs|45	NZ_CM000488:2383352	T>C	EX	ATG>GTG	M>V	heptaprenyl	nonsyn	
Bs|11	NZ_CM000488:2396273	C>T	EX	GAA>AAA	E>K	hypothetical	nonsyn	
Bs|26	NZ_CM000488:2403866	A>G	EX	TGG>CGG	W>R	hypothetical	nonsyn	
Bs|55	NZ_CM000488:2409337	G>A	EX	CCG>TCG	P>S	hypothetical	nonsyn	seq
Bs|49	NZ_CM000488:2415148	C>T	EX	CGG>CAG	R>Q	two-component	nonsyn	
Bs|20	NZ_CM000488:2422476	G>A	EX	GCC>GTC	A>V	spore	nonsyn	
Bs|1	NZ_CM000488:2422749	G>A	EX	CCG>TCG	P>S	D-alanyl-D-alanine	nonsyn	seq
Bs|17	NZ_CM000488:2441354	A>G	EX	TGG>CGG	W>R	sporulation	nonsyn	
Bs|11	NZ_CM000488:2451304	G>A	EX	CCG>CTG	P>L	malolactic	nonsyn	
Bs|71	NZ_CM000488:2455294	T>C	EX	TAC>TGC	Y>C	L-asparaginase	nonsyn	
Bs|48	NZ_CM000488:2477618	T>C	EX	AAG>GAG	K>E	ribonuclease	nonsyn	
Bs|71	NZ_CM000488:2480215	G>A	EX	GCG>GTG	A>V	6-phosphogluconate	nonsyn	
Bs|15	NZ_CM000488:2486374	A>G	EX	TTA>TCA	L>S	hypothetical	nonsyn	
Bs|38	NZ_CM000488:2488616	A>C	EX	ATG>AGG	M>R	hypothetical	nonsyn	
Bs|3	NZ_CM000488:2491962	C>T	IG					
Bs|13	NZ_CM000488:2493177	G>A	EX	GGA>GAA	G>E	multidrug	nonsyn	

Bs|38	NZ_CM000488:2493385	T>A	EX	ATT>ATA	I>I	multidrug	syn	
Bs|17	NZ_CM000488:2505101	G>T	EX	CCG>ACG	P>T	transcriptional	nonsyn	
Bs|31	NZ_CM000488:2514114	T>C	IG					
Bs|4	NZ_CM000488:2521363	A>C	EX	CTT>CGT	L>R	DNA	nonsyn	seq
Bs|44	NZ_CM000488:2536274	G>A	EX	GCC>GCT	A>A	mutants	syn	
Bs|37	NZ_CM000488:2547916	C>T	EX	TGG>TGA	W>*	glycine	nonsyn	
Bs|65	NZ_CM000488:2552669	A>T	EX	CTT>CAT	L>H	translocation-dependent	nonsyn	
Bs|26	NZ_CM000488:2553444	T>G	EX	ATT>CTT	I>L	type	nonsyn	seq
Bs|22	NZ_CM000488:2554482	T>C	IG					
Bs|32	NZ_CM000488:2568229	T>C	EX	ACC>GCC	T>A	hypothetical	nonsyn	seq
Bs|14	NZ_CM000488:2592655	C>T	EX	CTG>CTA	L>L	endonuclease	syn	
Bs|49	NZ_CM000488:2602147	T>C	EX	ATA>ATG	I>M	DNA	nonsyn	
Bs|45	NZ_CM000488:2604517	A>T	EX	GTG>GAG	V>E	hypothetical	nonsyn	
Bs|27	NZ_CM000488:2611365	A>G	EX	GTT>GTC	V>V	putative	syn	
Bs|69	NZ_CM000488:2627094	T>C	EX	GCA>GCG	A>A	class	syn	
Bs|8	NZ_CM000488:2632687	A>C	EX	AGT>AGG	S>R	required	nonsyn	
Bs|60	NZ_CM000488:2633074	G>A	EX	CAC>CAT	H>H	required	syn	
Bs|11	NZ_CM000488:2634837	T>C	IG					
Bs|63	NZ_CM000488:2660640	T>G	EX	TCC>GCC	S>A	hypothetical	nonsyn	
Bs|7	NZ_CM000488:2672636	G>A	EX	AGC>AGT	S>S	hypothetical	syn	
Bs|49	NZ_CM000488:2687116	A>C	EX	AAT>AAG	N>K	hypothetical	nonsyn	seq
Bs|45	NZ_CM000488:2690431	T>C	EX	AAA>GAA	K>E	hypothetical	nonsyn	
Bs|48	NZ_CM000488:2715058	A>C	EX	CTG>CGG	L>R	hypothetical	nonsyn	
Bs|20	NZ_CM000488:2725031	G>A	IG					
Bs|35	NZ_CM000488:2738566	A>G	IG					
Bs|7	NZ_CM000488:2741608	A>C	EX	TTA>GTA	L>V	RNA	nonsyn	
Bs|32	NZ_CM000488:2746214	T>C	EX	GTG>GCG	V>A	hypothetical	nonsyn	
Bs|26	NZ_CM000488:2762817	T>G	EX	ACC>CCC	T>P	NifA/NtrC	nonsyn	
Bs|22	NZ_CM000488:2773493	G>A	EX	GCA>GTA	A>V	hypothetical	nonsyn	seq
Bs|38	NZ_CM000488:2786088	T>C	EX	AAA>GAA	K>E	hypothetical	nonsyn	
Bs|20	NZ_CM000488:2798615	T>C	EX	AAA>GAA	K>E	alanyl-tRNA	nonsyn	
Bs|26	NZ_CM000488:2839854	C>A	EX	TTC>TTA	F>L	YrzF	nonsyn	
Bs|6	NZ_CM000488:2848696	T>C	IG					seq
Bs|54	NZ_CM000488:2856726	T>C	EX	CAG>CGG	Q>R	inhibition	nonsyn	
Bs|49	NZ_CM000488:2894422	T>C	EX	GCA>GCG	A>A	acetolactate	syn	
Bs|69	NZ_CM000488:2913378	T>C	EX	GAA>GGA	E>G	alpha-L-arabinosidase	nonsyn	
Bs|7	NZ_CM000488:2916561	C>T	EX	GCG>GCA	A>A	enoyl-CoA	syn	
Bs|7	NZ_CM000488:2924745	T>C	EX	CAC>CGC	H>R	hypothetical	nonsyn	
Bs|52	NZ_CM000488:2934603	C>T	EX	CTC>CTT	L>L	hypothetical	syn	
Bs|55	NZ_CM000488:2942235	G>A	EX	CCG>TCG	P>S	arabinose	nonsyn	
Bs|30	NZ_CM000488:2943679	T>C	EX	GAA>GGA	E>G	arabinose	nonsyn	
Bs|60	NZ_CM000488:2959963	T>C	EX	CAC>CGC	H>R	threonyl-tRNA	nonsyn	
Bs|35	NZ_CM000488:2963717	T>C	EX	GAA>GGA	E>G	membrane	nonsyn	
Bs|30	NZ_CM000488:2975894	G>A	EX	CCG>CTG	P>L	two-component	nonsyn	
Bs|7	NZ_CM000488:2976801	G>A	EX	CGC>TGC	R>C	two-component	nonsyn	
Bs|62	NZ_CM000488:2982646	T>C	EX	CTG>CCG	L>P	hypothetical	nonsyn	
Bs|51	NZ_CM000488:2987660	A>G	EX	CTA>CCA	L>P	acetyl-CoA	nonsyn	
Bs|45	NZ_CM000488:2990888	A>G	EX	TTT>TTC	F>F	DNA	syn	
Bs|57	NZ_CM000488:3001983	A>C	EX	GTT>GGT	V>G	hypothetical	nonsyn	
Bs|17	NZ_CM000488:3014116	C>T	IG					
Bs|17	NZ_CM000488:3014117	A>T	IG					
Bs|40	NZ_CM000488:3021499	C>T	EX	AGC>AAC	S>N	hypothetical	nonsyn	
Bs|52	NZ_CM000488:3034470	T>C	IG					
Bs|41	NZ_CM000488:3046432	T>C	EX	TTA>TTG	L>L	hypothetical	syn	
Bs|60	NZ_CM000488:3046936	T>C	EX	CTA>CTG	L>L	hypothetical	syn	seq
Bs|2	NZ_CM000488:3049575	C>T	IG					
Bs|1	NZ_CM000488:3057130	G>A	EX	AGC>AGT	S>S	malate	syn	seq
Bs|63	NZ_CM000488:3058144	G>T	EX	TGC>TGA	C>*	hypothetical	nonsyn	
Bs|43	NZ_CM000488:3062178	G>A	EX	ATC>ATT	I>I	pullulanase	syn	

Bs|46	NZ_CM000488:3071818	T>G	IG					seq
Bs|52	NZ_CM000488:3082500	A>T	EX	TAC>TTC	Y>F	hypothetical	nonsyn	
Bs|26	NZ_CM000488:3085315	G>A	EX	CCG>CTG	P>L	hypothetical	nonsyn	
Bs|2	NZ_CM000488:3086881	A>G	EX	TTT>TTC	F>F	hypothetical	syn	seq
Bs|48	NZ_CM000488:3090224	C>A	EX	GGC>TGC	G>C	biotin	nonsyn	
Bs|62	NZ_CM000488:3094539	A>G	IG					
Bs|46	NZ_CM000488:3130586	C>T	EX	AAG>AAA	K>K	hypothetical	syn	
Bs|2	NZ_CM000488:3164354	T>C	EX	AAA>GAA	K>E	glycogen	nonsyn	seq
Bs|28	NZ_CM000488:3177536	T>C	IG					
Bs|68	NZ_CM000488:3205251	G>A	EX	AGC>AGT	S>S	methyl-accepting	syn	
Bs|57	NZ_CM000488:3205342	T>C	EX	CAC>CGC	H>R	methyl-accepting	nonsyn	
Bs|50	NZ_CM000488:3207261	T>C	IG					
Bs|43	NZ_CM000488:3231026	G>T	EX	CGG>AGG	R>R	sporulation	syn	
Bs|35	NZ_CM000488:3232518	C>T	EX	CTC>TTC	L>F	hypothetical	nonsyn	seq
Bs|55	NZ_CM000488:3264862	T>C	EX	GAC>GGC	D>G	short	nonsyn	
Bs|11	NZ_CM000488:3287821	G>A	EX	CCC>TCC	P>S	2,3-dihydroxybenzoate-AMP	nonsyn	seq
Bs|60	NZ_CM000488:3317605	G>A	EX	GCA>GTA	A>V	hypothetical	nonsyn	seq
Bs|15	NZ_CM000488:3318211	G>A	EX	TTC>TTT	F>F	hypothetical	syn	
Bs|74	NZ_CM000488:3323458	G>A	EX	CTC>CTT	L>L	hypothetical	syn	
Bs|74	NZ_CM000488:3329546	C>T	EX	CTC>TTC	L>F	uric	nonsyn	seq
Bs|44	NZ_CM000488:3346730	A>G	EX	GTT>GCT	V>A	hypothetical	nonsyn	
Bs|53	NZ_CM000488:3393986	C>T	EX	GCG>GCA	A>A	hypothetical	syn	
Bs|40	NZ_CM000488:3394547	G>A	EX	CGG>TGG	R>W	hypothetical	nonsyn	
Bs|44	NZ_CM000488:3395222	G>A	EX	AGC>AGT	S>S	hypothetical	syn	
Bs|57	NZ_CM000488:3408022	C>G	EX	GCG>GCC	A>A	hypothetical	syn	
Bs|57	NZ_CM000488:3408460	C>T	IG					
Bs|17	NZ_CM000488:3442860	C>A	IG					seq
Bs|44	NZ_CM000488:3471640	G>A	EX	GCG>ACG	A>T	hypothetical	nonsyn	
Bs|74	NZ_CM000488:3478975	T>G	EX	CAA>CCA	Q>P	triosephosphate	nonsyn	
Bs|20	NZ_CM000488:3509165	C>T	EX	GCA>ACA	A>T	YvfI	nonsyn	
Bs|26	NZ_CM000488:3515627	T>C	EX	AAA>GAA	K>E	hypothetical	nonsyn	
Bs|62	NZ_CM000488:3536896	G>A	EX	GGG>GAG	G>E	hypothetical	nonsyn	
Bs|6	NZ_CM000488:3557526	T>C	EX	GAA>GAG	E>E	hypothetical	syn	seq
Bs|27	NZ_CM000488:3563754	A>G	EX	TAC>CAC	Y>H	hypothetical	nonsyn	
Bs|50	NZ_CM000488:3633821	T>C	IG					
Bs|40	NZ_CM000488:3653031	C>T	IG					
Bs|46	NZ_CM000488:3666701	T>C	EX	AGG>GGG	R>G	membrane-bound	nonsyn	seq
Bs|62	NZ_CM000488:3678489	C>A	EX	GAG>GAT	E>D	UDP-glucose:polyglycerol	nonsyn	
Bs|27	NZ_CM000488:3692310	G>A	EX	GCG>GTG	A>V	hypothetical	nonsyn	
Bs|44	NZ_CM000488:3698952	G>A	EX	CTC>CTT	L>L	hypothetical	syn	
Bs|30	NZ_CM000488:3735752	C>T	EX	AAC>AAT	N>N	hypothetical	syn	
Bs|50	NZ_CM000488:3744710	G>T	EX	CAG>AAG	Q>K	flagellar	nonsyn	
Bs|40	NZ_CM000488:3754454	C>T	EX	GGG>AGG	G>R	hypothetical	nonsyn	
Bs|52	NZ_CM000488:3755722	T>C	IG					seq
Bs|19	NZ_CM000488:3765396	A>C	EX	GGT>GGG	G>G	hypothetical	syn	
Bs|36	NZ_CM000488:3798374	C>T	EX	GCG>GCA	A>A	hypothetical	syn	
Bs|48	NZ_CM000488:3803924	T>C	IG					seq
Bs|4	NZ_CM000488:3804463	G>A	EX	GCT>GTT	A>V	fructose	nonsyn	seq
Bs|14	NZ_CM000488:3809172	T>G	EX	CTG>CGG	L>R	hypothetical	nonsyn	
Bs|1	NZ_CM000488:3814530	A>T	EX	TGG>AGG	W>R	hypothetical	nonsyn	seq
Bs|57	NZ_CM000488:3818544	G>A	EX	TTC>TTT	F>F	hypothetical	syn	
Bs|24	NZ_CM000488:3821104	G>A	EX	GGC>GGT	G>G	hypothetical	syn	
Bs|22	NZ_CM000488:3835012	A>G	IG					
Bs|28	NZ_CM000488:3855430	A>T	IG					seq
Bs|19	NZ_CM000488:3856077	A>T	EX	CTC>CAC	L>H	methylenomycin	nonsyn	
Bs|26	NZ_CM000488:3861032	A>C	EX	CTA>CTC	L>L	transcriptional	syn	
Bs|31	NZ_CM000488:3870542	T>C	EX	ACA>GCA	T>A	hypothetical	nonsyn	
Bs|51	NZ_CM000488:3873281	C>A	IG					
Bs|3	NZ_CM000488:3876855	T>C	EX	GAA>GGA	E>G	involved	nonsyn	seq

Bs|69	NZ_CM000488:3879709	A>T	IG					
Bs|24	NZ_CM000488:3881779	A>T	IG					
Bs|8	NZ_CM000488:3917914	T>C	EX	TGG>CGG	W>R	hypothetical	nonsyn	
Bs|17	NZ_CM000488:3924882	A>G	EX	GTT>GCT	V>A	hypothetical	nonsyn	
Bs|45	NZ_CM000488:3931707	T>C	EX	ATA>GTA	I>V	hypothetical	nonsyn	
Bs|65	NZ_CM000488:3978656	T>A	IG					
Bs|14	NZ_CM000488:4005558	T>C	EX	GTA>GCA	V>A	hypothetical	nonsyn	seq
Bs|45	NZ_CM000488:4025960	T>C	EX	GAA>GGA	E>G	cell	nonsyn	
Bs|41	NZ_CM000488:4053309	G>A	IG					
Bs|50	NZ_CM000488:4059405	A>G	EX	CAT>CAC	H>H	hypothetical	syn	
Bs|38	NZ_CM000488:4061406	G>A	EX	GGC>GGT	G>G	hypothetical	syn	
Bs|17	NZ_CM000488:4074623	A>T	EX	TAT>AAT	Y>N	inositol	nonsyn	
Bs|7	NZ_CM000488:4086475	T>C	EX	GTA>GCA	V>A	hypothetical	nonsyn	seq
Bs|19	NZ_CM000488:4117840	G>A	IG					
Bs|14	NZ_CM000488:4130603	T>C	EX	TAT>TGT	Y>C	hypothetical	nonsyn	seq
Bs|51	NZ_CM000488:4132788	T>C	EX	AAA>GAA	K>E	hypothetical	nonsyn	
Bs|57	NZ_CM000488:4138637	G>A	IG					
Bs|51	NZ_CM000488:4151353	T>C	EX	AAA>GAA	K>E	hypothetical	nonsyn	
Bs|44	NZ_CM000488:4155800	T>C	IG					seq
Bs|45	NZ_CM000488:4156086	G>A	EX	CCG>CTG	P>L	hypothetical	nonsyn	seq
Bs|55	NZ_CM000488:4158225	G>T	IG					
Bs|31	NZ_CM000488:4173504	T>C	EX	TCA>CCA	S>P	hypothetical	nonsyn	
Bs|71	NZ_CM000488:4175417	A>T	EX	CAT>CTT	H>L	hypothetical	nonsyn	
Bs|36	NZ_CM000488:4180435	G>T	IG					
Bs|52	NZ_CM000488:4193254	A>G	IG					
Bs|15	NZ_CM000488:4200542	C>T	EX	TGG>TGA	W>*	hypothetical	nonsyn	
Ec|83	NC_000913:1927	C>T	EX	CCG>TCG	P>S	fused	nonsyn	
Ec|48	NC_000913:13313	G>A	EX	GGT>GAT	G>D	chaperone	nonsyn	
Ec|75	NC_000913:20158	T>G	EX	AAA>CAA	K>Q	IS1	nonsyn	
Ec|11	NC_000913:30799	A>C	IG					
Ec|55	NC_000913:34237	C>T	IG					
Ec|50	NC_000913:60747	G>C	EX	CCG>GCG	P>A	RNA	nonsyn	
Ec|61	NC_000913:60965	A>G	EX	GTG>GCG	V>A	RNA	nonsyn	
Ec|35	NC_000913:77036	A>C	EX	GAT>GAG	D>E	transcriptional	nonsyn	
Ec|17	NC_000913:79763	C>T	EX	GCG>ACG	A>T	3-isopropylmalate	nonsyn	
Ec|99	NC_000913:80680	T>G	EX	GAT>GCT	D>A	3-isopropylmalate	nonsyn	
Ec|84	NC_000913:107482	T>G	IG					
Ec|8	NC_000913:119211	A>C	EX	GAT>GCT	D>A	1,6-anhydro-N-acetylmuramyl-L-alanine	nonsyn	
Ec|70	NC_000913:120366	G>T	EX	CGC>AGC	R>S	aromatic	nonsyn	
Ec|68	NC_000913:122177	G>A	EX	CGC>CAC	R>H	DNA-binding	nonsyn	
Ec|16	NC_000913:136363	A>G	EX	GAT>GAC	D>D	spermidine	syn	
Ec|12	NC_000913:146593	G>A	EX	GAC>GAT	D>D	aspartate	syn	
Ec|45	NC_000913:160881	C>G	EX	TTG>TTC	L>F	putative	nonsyn	
Ec|44	NC_000913:182689	A>C	EX	CAG>CCG	Q>P	DNA-binding	nonsyn	
Ec|28	NC_000913:186442	C>T	EX	GAC>AAC	D>N	uridylyltransferase	nonsyn	
Ec|39	NC_000913:220368	C>T	EX	CTG>CTA	L>L	DL-methionine	syn	
Ec|11	NC_000913:222754	G>A	IG					
Ec|39	NC_000913:223463	C>T	IG					
Ec|87	NC_000913:231608	G>T	EX	GGC>TGC	G>C	hypothetical	nonsyn	
Ec|28	NC_000913:231655	A>C	EX	ATA>ATC	I>I	hypothetical	syn	
Ec|71	NC_000913:236978	T>G	IG					
Ec|39	NC_000913:246540	A>G	IG					
Ec|65	NC_000913:251974	C>A	IG					
Ec|50	NC_000913:256366	G>A	EX	CCG>CCA	P>P	guanine-hypoxanthine	syn	
Ec|79	NC_000913:284486	A>G	IG					
Ec|16	NC_000913:286648	C>T	EX	GTC>GTT	V>V	CP4-6	syn	
Ec|94	NC_000913:286742	C>T	EX	CAG>TAG	Q>*	CP4-6	nonsyn	
Ec|8	NC_000913:340069	T>G	EX	ATT>ATG	I>M	carbamate	nonsyn	
Ec|50	NC_000913:344490	T>G	IG					

Ec|28	NC_000913:353886	T>C	IG				
Ec|28	NC_000913:353893	T>G	IG				
Ec|21	NC_000913:377367	C>T	EX	GTT>ATT	V>I	S-formylglutathione	nonsyn
Ec|62	NC_000913:386645	T>G	EX	TTA>GTA	L>V	taurine	nonsyn
Ec|70	NC_000913:389285	A>G	IG				
Ec|38	NC_000913:396478	G>C	EX	GGC>CGC	G>R	microcin	nonsyn
Ec|35	NC_000913:471102	C>T	EX	CAG>TAG	Q>*	fused	nonsyn
Ec|11	NC_000913:498995	T>A	EX	AAA>ATA	K>I	acetyl	nonsyn
Ec|6	NC_000913:526865	A>G	EX	ACA>GCA	T>A	hypothetical	nonsyn
Ec|90	NC_000913:536137	C>T	EX	CTG>TTG	L>L	tartronate	syn
Ec|38	NC_000913:537546	G>A	EX	CTG>CTA	L>L	putative	syn
Ec|29	NC_000913:540043	C>T	EX	ATC>ATT	I>I	putative	syn
Ec|13	NC_000913:543079	G>A	EX	GTC>GTT	V>V	hypothetical	syn
Ec|65	NC_000913:557219	C>T	IG				
Ec|49	NC_000913:617563	A>G	EX	TTA>TTG	L>L	regulator	syn
Ec|44	NC_000913:646804	G>T	EX	GCA>GAA	A>E	apo-citrate	nonsyn
Ec|94	NC_000913:651482	C>T	EX	CAG>TAG	Q>*	sensory	nonsyn
Ec|41	NC_000913:665947	C>T	EX	CAG>CAA	Q>Q	transpeptidase	syn
Ec|25	NC_000913:682663	C>T	IG				
Ec|74	NC_000913:691456	C>T	EX	GAA>AAA	E>K	metal-binding	nonsyn
Ec|53	NC_000913:700115	A>C	EX	TTT>GTT	F>V	DNA-binding	nonsyn
Ec|61	NC_000913:730434	T>C	EX	ACT>ACC	T>T	rhsC	syn
Ec|65	NC_000913:731563	G>A	EX	GCC>ACC	A>T	rhsC	nonsyn
Ec|39	NC_000913:736113	A>G	IG				
Ec|39	NC_000913:736115	A>T	IG				
Ec|39	NC_000913:736174	A>G	IG				
Ec|47	NC_000913:753915	A>C	IG				
Ec|66	NC_000913:774426	T>G	EX	GTT>GTG	V>V	membrane	syn
Ec|50	NC_000913:782795	G>C	EX	CGC>CCC	R>P	nicotinamide	nonsyn
Ec|89	NC_000913:785034	C>T	EX	CCA>CTA	P>L	3-deoxy-D-arabino-heptulosonate-7-phospl	nonsyn
Ec|68	NC_000913:816164	C>T	IG				
Ec|81	NC_000913:858330	G>A	IG				
Ec|48	NC_000913:872465	A>C	EX	AAA>AAC	K>N	putative	nonsyn
Ec|82	NC_000913:884568	G>T	EX	CTT>ATT	L>I	FMN	nonsyn
Ec|63	NC_000913:890423	A>G	EX	GAA>GGA	E>G	nitroreductase	nonsyn
Ec|7	NC_000913:897304	C>G	EX	GCC>GCG	A>A	inner	syn
Ec|63	NC_000913:898959	G>A	IG				
Ec|41	NC_000913:906575	C>A	EX	GCT>TCT	A>S	conserved	nonsyn
Ec|59	NC_000913:987136	T>C	EX	AAC>AGC	N>S	asparaginyl	nonsyn
Ec|28	NC_000913:990526	A>C	EX	ACC>CCC	T>P	aminopeptidase	nonsyn
Ec|55	NC_000913:1019090	C>G	EX	GGT>CGT	G>R	outer	nonsyn
Ec|16	NC_000913:1023067	C>T	EX	GCC>ACC	A>T	inner	nonsyn
Ec|46	NC_000913:1043601	T>C	EX	GAG>GGG	E>G	phosphotyrosine-protein	nonsyn
Ec|65	NC_000913:1054504	A>G	EX	TTT>CTT	F>L	hybrid	nonsyn
Ec|85	NC_000913:1067631	G>T	IG				
Ec|10	NC_000913:1074021	C>T	EX	GCG>GTG	A>V	DNA-binding	nonsyn
Ec|29	NC_000913:1083417	C>T	EX	CGC>CGT	R>R	deferrochelatase,	syn
Ec|74	NC_000913:1102222	C>T	EX	AAG>AAA	K>K	DNA-binding	syn
Ec|44	NC_000913:1103010	T>C	IG				
Ec|8	NC_000913:1104543	A>C	IG				
Ec|41	NC_000913:1127435	C>G	EX	GCC>GGC	A>G	putative	nonsyn
Ec|46	NC_000913:1173231	G>T	IG				
Ec|49	NC_000913:1186360	A>G	EX	TAT>TAC	Y>Y	cupin	syn
Ec|74	NC_000913:1189131	C>T	EX	CCG>CCA	P>P	DNA-binding	syn
Ec|75	NC_000913:1201412	T>C	IG				
Ec|50	NC_000913:1222442	A>C	IG				
Ec|21	NC_000913:1242881	A>T	EX	GAT>GTT	D>V	lytic	nonsyn
Ec|49	NC_000913:1301784	T>G	EX	TTG>GTG	L>V	oligopeptide	nonsyn
Ec|49	NC_000913:1311017	T>C	EX	AAC>AGC	N>S	inner	nonsyn

Ec|81	NC_000913:1334655	G>A	EX	CTG>CTA	L>L	aconitate	syn
Ec|82	NC_000913:1338450	C>T	EX	CTG>TTG	L>L	DUF1049	syn
Ec|74	NC_000913:1374071	C>T	EX	ACA>ATA	T>I	putative	nonsyn
Ec|45	NC_000913:1384979	A>T	EX	CAT>CTT	H>L	DNA-binding	nonsyn
Ec|63	NC_000913:1393393	A>T	EX	ATC>AAC	I>N	mechanosensitive	nonsyn
Ec|39	NC_000913:1408092	C>T	EX	ACC>ACT	T>T	ATP-dependent	syn
Ec|83	NC_000913:1416308	G>A	IG				
Ec|85	NC_000913:1424333	G>T	IG				
Ec|28	NC_000913:1435682	C>T	EX	GGC>AGC	G>S	fused	nonsyn
Ec|39	NC_000913:1522323	T>A	EX	AGT>AGA	S>R	ATP-binding	nonsyn
Ec|83	NC_000913:1535872	A>T	EX	TGG>AGG	W>R	nitrate	nonsyn
Ec|80	NC_000913:1542879	C>A	IG				
Ec|35	NC_000913:1547775	C>T	EX	GCG>GTG	A>V	formate	nonsyn
Ec|56	NC_000913:1558664	A>G	EX	TTC>TCC	F>S	D-ala-D-ala	nonsyn
Ec|7	NC_000913:1605686	T>A	EX	CTG>CAG	L>Q	trans-aconitate	nonsyn
Ec|53	NC_000913:1609699	C>G	EX	GCG>GCC	A>A	putative	syn
Ec|45	NC_000913:1612205	C>A	EX	GTG>TTG	V>L	succinate	nonsyn
Ec|59	NC_000913:1642727	G>A	EX	GCA>GTA	A>V	Qin	nonsyn
Ec|41	NC_000913:1665800	G>A	EX	GGC>GGT	G>G	global	syn
Ec|12	NC_000913:1666404	T>C	EX	GAG>GGG	E>G	global	nonsyn
Ec|66	NC_000913:1681615	G>A	EX	AGC>AAC	S>N	sensory	nonsyn
Ec|63	NC_000913:1695209	T>C	IG				
Ec|23	NC_000913:1702041	T>C	EX	ACC>GCC	T>A	putative	nonsyn
Ec|59	NC_000913:1702790	T>G	IG				
Ec|63	NC_000913:1718872	G>A	IG				
Ec|63	NC_000913:1772674	G>A	EX	GCC>ACC	A>T	quinate/shikimate	nonsyn
Ec|92	NC_000913:1773491	A>G	IG				
Ec|29	NC_000913:1775436	G>C	EX	GCG>CCG	A>P	putative	nonsyn
Ec|29	NC_000913:1788431	G>T	EX	CGT>AGT	R>S	conserved	nonsyn
Ec|6	NC_000913:1791978	G>A	EX	GCC>GCT	A>A	glutathione	syn
Ec|84	NC_000913:1801027	C>A	IG				
Ec|78	NC_000913:1825900	G>A	EX	ACC>ACT	T>T	succinylarginine	syn
Ec|92	NC_000913:1839765	G>C	EX	TGG>TGC	W>C	CTP	nonsyn
Ec|41	NC_000913:1842577	C>A	EX	GAG>TAG	E>*	inner	nonsyn
Ec|99	NC_000913:1855545	A>G	EX	GGT>GGC	G>G	putative	syn
Ec|61	NC_000913:1856410	G>A	EX	CTG>TTG	L>L	putative	syn
Ec|28	NC_000913:1882064	A>C	EX	AAG>CAG	K>Q	putative	nonsyn
Ec|6	NC_000913:1900116	G>A	EX	GAG>GAA	E>E	fused	syn
Ec|10	NC_000913:1920961	A>C	EX	ATT>ATG	I>M	serine/threonine-specific	nonsyn
Ec|38	NC_000913:1942219	G>C	EX	AGC>ACC	S>T	zinc	nonsyn
Ec|77	NC_000913:1948118	T>C	EX	AAA>AAG	K>K	aspartyl-tRNA	syn
Ec|99	NC_000913:1974613	A>C	EX	GTC>GGC	V>G	proton	nonsyn
Ec|11	NC_000913:1984131	G>A	EX	CTG>TTG	L>L	L-arabinose	syn
Ec|46	NC_000913:1990116	T>G	IG				
Ec|82	NC_000913:2009458	C>G	IG				
Ec|50	NC_000913:2057939	T>C	IG				
Ec|12	NC_000913:2069933	A>C	EX	AAC>ACC	N>T	CP4-44	nonsyn
Ec|35	NC_000913:2073132	T>G	EX	GCT>GCG	A>A	CP4-44	syn
Ec|62	NC_000913:2085248	C>T	IG				
Ec|14	NC_000913:2110939	A>T	EX	TCT>TCA	S>S	dTDP-glucose	syn
Ec|69	NC_000913:2136501	C>A	EX	TTC>TTA	F>L	inner	nonsyn
Ec|6	NC_000913:2154439	G>A	EX	GTT>ATT	V>I	multidrug	nonsyn
Ec|13	NC_000913:2173517	G>T	EX	GCA>GAA	A>E	D-tagatose	nonsyn
Ec|13	NC_000913:2183882	T>C	IG				
Ec|44	NC_000913:2186233	C>T	EX	CCG>CCA	P>P	putative	syn
Ec|85	NC_000913:2192922	A>T	EX	ATG>TTG	M>L	methionyl-tRNA	nonsyn
Ec|27	NC_000913:2227875	G>A	EX	GCG>GTG	A>V	tRNA-dihydrouridine	nonsyn
Ec|61	NC_000913:2231696	A>C	EX	AAA>AAC	K>N	conserved	nonsyn
Ec|23	NC_000913:2301727	T>C	EX	TAT>CAT	Y>H	hypothetical	nonsyn

Ec|35	NC_000913:2312739	G>T	EX	GCG>GCT	A>A	phosphotransfer	syn
Ec|59	NC_000913:2334355	G>A	EX	AGC>AGT	S>S	DUF2138	syn
Ec|80	NC_000913:2341516	C>T	EX	GTA>ATA	V>I	adhesin	nonsyn
Ec|78	NC_000913:2342414	A>G	IG				
Ec|5	NC_000913:2355276	G>A	EX	GTG>GTA	V>V	hypothetical	syn
Ec|99	NC_000913:2376697	G>C	EX	GAC>GAG	D>E	bifunctional	nonsyn
Ec|67	NC_000913:2397470	C>T	EX	GGT>AGT	G>S	NADH:ubiquinone	nonsyn
Ec|66	NC_000913:2411969	G>T	EX	GAG>TAG	E>*	acetate	nonsyn
Ec|14	NC_000913:2442906	C>T	EX	GGT>GAT	G>D	inner	nonsyn
Ec|81	NC_000913:2475550	C>T	EX	CTG>CTA	L>L	DNA-binding	syn
Ec|50	NC_000913:2478388	G>C	EX	GCG>CCG	A>P	D-serine	nonsyn
Ec|71	NC_000913:2485186	A>G	EX	ACG>GCG	T>A	hybrid	nonsyn
Ec|16	NC_000913:2491655	G>T	IG				
Ec|23	NC_000913:2531316	G>A	EX	GTG>ATG	V>M	cysteine	nonsyn
Ec|53	NC_000913:2558999	T>A	IG				
Ec|56	NC_000913:2562504	T>C	IG				
Ec|80	NC_000913:2562517	G>T	IG				
Ec|99	NC_000913:2597309	G>A	EX	ATC>ATT	I>I	dihydrodipicolinate	syn
Ec|44	NC_000913:2629826	T>G	EX	GCA>GCC	A>A	GMP	syn
Ec|66	NC_000913:2641447	C>A	EX	GAC>TAC	D>Y	dual	nonsyn
Ec|68	NC_000913:2641821	G>A	EX	GCA>GTA	A>V	dual	nonsyn
Ec|63	NC_000913:2649622	G>A	EX	CAG>TAG	Q>*	hypothetical	nonsyn
Ec|66	NC_000913:2653740	G>T	EX	TCG>TAG	S>*	aminopeptidase	nonsyn
Ec|59	NC_000913:2720716	A>C	IG				
Ec|96	NC_000913:2771854	C>T	EX	TCC>TTC	S>F	CP4-57	nonsyn
Ec|29	NC_000913:2795582	T>C	EX	TTA>TCA	L>S	putative	nonsyn
Ec|49	NC_000913:2816581	T>C	IG				
Ec|39	NC_000913:2836375	T>C	EX	GGA>GGG	G>G	DNA-binding	syn
Ec|65	NC_000913:2856362	G>A	EX	CCG>CCA	P>P	methyl-directed	syn
Ec|16	NC_000913:2857900	T>C	EX	TTC>TCC	F>S	serine/threonine-specific	nonsyn
Ec|59	NC_000913:2872430	A>G	EX	TCG>CCG	S>P	sulfate	nonsyn
Ec|29	NC_000913:2879898	A>G	EX	GAT>GAC	D>D	CRISP	syn
Ec|41	NC_000913:2909122	T>G	EX	GAA>GAC	E>D	antitoxin	nonsyn
Ec|37	NC_000913:2914325	T>A	EX	CTG>CAG	L>Q	hybrid	nonsyn
Ec|91	NC_000913:2932467	T>C	EX	TAC>CAC	Y>H	L-fucose	nonsyn
Ec|68	NC_000913:2939183	T>G	EX	GAA>GCA	E>A	23S	nonsyn
Ec|70	NC_000913:2941560	G>T	EX	GCA>TCA	A>S	cysteine	nonsyn
Ec|12	NC_000913:2961471	A>T	EX	TGG>AGG	W>R	hypothetical	nonsyn
Ec|44	NC_000913:2993131	T>C	IG				
Ec|66	NC_000913:2993903	C>G	IG				
Ec|8	NC_000913:3003313	T>C	EX	ATC>GTC	I>V	putative	nonsyn
Ec|65	NC_000913:3003735	G>A	EX	GCC>GTC	A>V	putative	nonsyn
Ec|69	NC_000913:3008229	G>A	EX	ACG>ACA	T>T	D-stereospecific	syn
Ec|50	NC_000913:3025203	T>C	EX	TAT>CAT	Y>H	putative	nonsyn
Ec|39	NC_000913:3078108	A>C	EX	ATC>AGC	I>S	transketolase	nonsyn
Ec|44	NC_000913:3125646	A>C	EX	ATC>AGC	I>S	glycolate	nonsyn
Ec|85	NC_000913:3143361	T>A	EX	CAG>CTG	Q>L	hydrogenase	nonsyn
Ec|6	NC_000913:3154268	G>A	EX	GAT>AAT	D>N	aldehyde	nonsyn
Ec|82	NC_000913:3156659	T>G	EX	ATT>AGT	I>S	outer	nonsyn
Ec|11	NC_000913:3186716	G>A	IG				
Ec|79	NC_000913:3218861	T>C	EX	CTG>CCG	L>P	putrescine:2-oxoglutaric	nonsyn
Ec|59	NC_000913:3255556	A>C	EX	GGT>GGG	G>G	putative	syn
Ec|12	NC_000913:3265238	A>G	IG				
Ec|49	NC_000913:3272211	A>C	EX	CTG>CGG	L>R	putative	nonsyn
Ec|56	NC_000913:3303905	G>A	IG				
Ec|79	NC_000913:3313129	G>A	EX	GCT>GTT	A>V	fused	nonsyn
Ec|13	NC_000913:3319333	A>T	EX	GAT>GAA	D>E	putative	nonsyn
Ec|45	NC_000913:3368875	G>A	EX	CGT>TGT	R>C	putative	nonsyn
Ec|6	NC_000913:3371956	G>C	EX	CCA>GCA	P>A	DNA-binding	nonsyn

Ec|45	NC_000913:3375922	A>C	EX	TTC>TGC	F>C	30S	nonsyn
Ec|90	NC_000913:3377420	C>T	EX	CAG>CAA	Q>Q	conserved	syn
Ec|45	NC_000913:3403174	A>T	IG				
Ec|45	NC_000913:3403196	C>T	IG				
Ec|29	NC_000913:3420219	G>A	EX	GGG>GGA	G>G	putative	syn
Ec|63	NC_000913:3426974	A>G	IG				
Ec|41	NC_000913:3429821	A>G	EX	ATC>ACC	I>T	putative	nonsyn
Ec|14	NC_000913:3441706	A>G	EX	GGT>GGC	G>G	preprotein	syn
Ec|96	NC_000913:3446351	C>A	EX	GAG>GAT	E>D	30S	nonsyn
Ec|35	NC_000913:3450134	G>T	EX	CCG>ACG	P>T	50S	nonsyn
Ec|28	NC_000913:3450223	T>A	EX	CAC>CTC	H>L	50S	nonsyn
Ec|85	NC_000913:3451150	C>A	EX	CGC>CTC	R>L	30S	nonsyn
Ec|93	NC_000913:3451341	A>G	IG				
Ec|28	NC_000913:3487449	T>C	EX	ACC>GCC	T>A	bifunctional	nonsyn
Ec|39	NC_000913:3499177	G>A	EX	GGC>AGC	G>S	putative	nonsyn
Ec|12	NC_000913:3506601	A>C	EX	GAT>GAG	D>E	putative	nonsyn
Ec|29	NC_000913:3519211	C>T	EX	CTG>CTA	L>L	protein	syn
Ec|61	NC_000913:3519568	G>A	EX	CAG>TAG	Q>*	protein	nonsyn
Ec|44	NC_000913:3587984	T>G	EX	ATC>CTC	I>L	glycerol-3-phosphate	nonsyn
Ec|29	NC_000913:3620862	A>G	EX	CGA>CGG	R>R	rhsB	syn
Ec|59	NC_000913:3678925	G>A	EX	CTG>TTG	L>L	putative	syn
Ec|14	NC_000913:3704059	G>A	IG				
Ec|47	NC_000913:3752522	C>T	IG				
Ec|64	NC_000913:3796821	G>A	EX	GCA>GTA	A>V	lipopolysaccharide	nonsyn
Ec|49	NC_000913:3833632	G>T	EX	GGC>GGA	G>G	putative	syn
Ec|23	NC_000913:3913443	A>T	IG				
Ec|29	NC_000913:3939677	A>G	IG				
Ec|49	NC_000913:3944645	G>T	IG				
Ec|99	NC_000913:3944942	T>G	IG				
Ec|21	NC_000913:3950600	T>G	EX	TAT>GAT	Y>D	branched-chain	nonsyn
Ec|14	NC_000913:3991950	T>C	EX	AAT>GAT	N>D	iron-dependent	nonsyn
Ec|85	NC_000913:4030185	G>A	EX	CCG>CCA	P>P	proline	syn
Ec|85	NC_000913:4030296	C>T	EX	TGC>TGT	C>C	proline	syn
Ec|39	NC_000913:4033357	C>T	IG				
Ec|45	NC_000913:4036734	A>C	IG				
Ec|76	NC_000913:4075709	A>T	EX	AAT>TAT	N>Y	putative	nonsyn
Ec|28	NC_000913:4077138	C>T	IG				
Ec|48	NC_000913:4077216	A>G	IG				
Ec|62	NC_000913:4090438	G>A	EX	CAG>TAG	Q>*	putative	nonsyn
Ec|10	NC_000913:4166238	A>T	IG				
Ec|10	NC_000913:4166244	T>A	IG				
Ec|86	NC_000913:4182926	A>C	EX	CAG>CCG	Q>P	RNA	nonsyn
Ec|17	NC_000913:4199652	T>C	EX	GGA>GGG	G>G	Zn-dependent	syn
Ec|8	NC_000913:4206062	A>G	IG				
Ec|70	NC_000913:4248805	A>T	IG				
Ec|13	NC_000913:4267708	A>C	EX	GAA>GCA	E>A	acid	nonsyn
Ec|53	NC_000913:4284754	A>G	EX	GTA>GCA	V>A	acetyl-CoA	nonsyn
Ec|44	NC_000913:4327687	A>C	EX	GAT>GCT	D>A	mutational	nonsyn
Ec|74	NC_000913:4380098	C>T	EX	GTC>ATC	V>I	fumarate	nonsyn
Ec|65	NC_000913:4390487	C>G	IG				
Ec|63	NC_000913:4411810	G>A	EX	GTG>ATG	V>M	glutathionylspermidine	nonsyn
Ec|6	NC_000913:4417711	A>G	IG				
Ec|99	NC_000913:4431312	C>T	EX	GAT>AAT	D>N	NAD(P)H:quinone	nonsyn
Ec|35	NC_000913:4433910	C>T	EX	GGG>AGG	G>R	2':3'-cyclic-nucleotide	nonsyn
Ec|87	NC_000913:4442956	C>A	EX	ATC>ATA	I>I	translocation	syn
Ec|41	NC_000913:4451827	G>T	EX	GTG>GTT	V>V	putative	syn
Ec|86	NC_000913:4466603	T>G	EX	CTG>CGG	L>R	magnesium	nonsyn
Ec|38	NC_000913:4481228	T>A	EX	GCA>GCT	A>A	valyl-tRNA	syn
Ec|17	NC_000913:4502337	C>A	EX	GCA>GAA	A>E	putative	nonsyn

Ec|63	NC_000913:4515690	G>A	EX	TCC>TCT	S>S	KpLE2	syn
Ec|50	NC_000913:4522598	T>G	EX	ACC>CCC	T>P	KpLE2	nonsyn
Ec|55	NC_000913:4535483	C>T	EX	GCG>GCA	A>A	9-O-acetyl	syn
Ec|17	NC_000913:4580887	C>T	EX	CGC>CAC	R>H	DNA	nonsyn
Ec|85	NC_000913:4588983	G>A	EX	CCG>CTG	P>L	putative	nonsyn
Ec|81	NC_000913:4602391	G>T	EX	CGC>CTC	R>L	bgl	nonsyn
Ec|38	NC_000913:4612417	T>G	IG				
Mf|55	NC_006055:880	G>T	EX	GCC>TCC	A>S	chromosome	nonsyn
Mf|12	NC_006055:937	G>A	EX	GTT>ATT	V>I	chromosome	nonsyn
Mf|57	NC_006055:3688	T>A	IG				
Mf|50	NC_006055:3979	G>C	EX	GTT>CTT	V>L	nucleotidyltransferase,	nonsyn
Mf|14	NC_006055:5544	T>C	EX	GGT>GGC	G>G	DNA	syn
Mf|47	NC_006055:5851	G>A	EX	GAT>AAT	D>N	DNA	nonsyn
Mf|42	NC_006055:7958	T>A	EX	ACT>ACA	T>T	DNA	syn
Mf|92	NC_006055:8912	A>G	EX	TGA>TGG	*>W	DNA	nonsyn
Mf|14	NC_006055:9651	C>A	EX	CAA>AAA	Q>K	DNA	nonsyn
Mf|65	NC_006055:9903	G>A	EX	GCT>ACT	A>T	beta-glucoside	nonsyn
Mf|6	NC_006055:10267	G>A	EX	TGT>TAT	C>Y	beta-glucoside	nonsyn
Mf|39	NC_006055:11632	T>G	EX	ATG>AGG	M>R	beta-glucoside	nonsyn
Mf|47	NC_006055:13385	C>T	EX	CCA>CTA	P>L	beta-glucosidase	nonsyn
Mf|42	NC_006055:14099	C>A	EX	CGT>AGT	R>S	transcriptional	nonsyn
Mf|50	NC_006055:15075	T>A	EX	TAT>TAA	Y>*	beta-glucosidase	nonsyn
Mf|92	NC_006055:15415	C>T	EX	CAA>TAA	Q>*	beta-glucosidase	nonsyn
Mf|58	NC_006055:16165	G>A	EX	GAT>AAT	D>N	beta-glucosidase	nonsyn
Mf|58	NC_006055:17611	G>A	EX	GGA>AGA	G>R	beta-glucosidase	nonsyn
Mf|58	NC_006055:21424	C>T	EX	GGC>AGC	G>S	arginine-ornithine	nonsyn
Mf|42	NC_006055:23722	G>A	EX	TAC>TAT	Y>Y	seryl-tRNA	syn
Mf|83	NC_006055:24901	C>T	EX	AGC>AGT	S>S	hsp33	syn
Mf|57	NC_006055:27072	T>C	EX	GTT>GTC	V>V	glutamine	syn
Mf|11	NC_006055:27539	C>G	EX	ACA>AGA	T>R	glutamine	nonsyn
Mf|19	NC_006055:27981	C>G	EX	CCC>CGC	P>R	hypothetical	nonsyn
Mf|23	NC_006055:28346	G>T	EX	GCC>TCC	A>S	hypothetical	nonsyn
Mf|76	NC_006055:30387	G>T	EX	GAA>TAA	E>*	hypothetical	nonsyn
Mf|83	NC_006055:33998	G>T	EX	GGT>GTT	G>V	sn-glycerol-3-phosphate	nonsyn
Mf|42	NC_006055:34010	G>A	EX	CGT>CAT	R>H	sn-glycerol-3-phosphate	nonsyn
Mf|85	NC_006055:35692	G>T	EX	TTG>TTT	L>F	sn-glycerol-3-phosphate	nonsyn
Mf|56	NC_006055:37112	T>C	EX	GAT>GAC	D>D	sn-glycerol-3-phosphate	syn
Mf|68	NC_006055:38477	G>A	EX	GTA>ATA	V>I	sn-glycerol-3-phosphate	nonsyn
Mf|56	NC_006055:45632	G>C	EX	TTG>TTC	L>F	beta-glucoside	nonsyn
Mf|83	NC_006055:46675	C>T	EX	ACA>ATA	T>I	beta-glucoside	nonsyn
Mf|56	NC_006055:47390	G>A	EX	AGA>AAA	R>K	6-phospho-beta-glucosidase	nonsyn
Mf|11	NC_006055:47537	C>A	EX	TCA>TAA	S>*	6-phospho-beta-glucosidase	nonsyn
Mf|26	NC_006055:48797	T>C	EX	AAC>AGC	N>S	transporter	nonsyn
Mf|76	NC_006055:49225	T>C	EX	TTA>TTG	L>L	transporter	syn
Mf|11	NC_006055:49663	T>C	EX	ATT>ACT	I>T	methionyl-tRNA	nonsyn
Mf|19	NC_006055:52834	G>T	EX	GCG>GCT	A>A	lipoate-protein	syn
Mf|47	NC_006055:54986	G>T	EX	GGA>TGA	G>*	pyruvate	nonsyn
Mf|93	NC_006055:55726	C>G	EX	AAC>AAG	N>K	pyruvate	nonsyn
Mf|6	NC_006055:58529	C>G	EX	CCA>CGA	P>R	dihydrolipate	nonsyn
Mf|90	NC_006055:62693	A>G	EX	AAA>GAA	K>E	Mg2+	nonsyn
Mf|14	NC_006055:64420	C>A	IG				
Mf|76	NC_006055:65457	C>T	EX	GTT>ATT	V>I	MarR	nonsyn
Mf|57	NC_006055:67043	C>T	EX	GCT>ACT	A>T	multidrug	nonsyn
Mf|6	NC_006055:68595	C>T	EX	TGA>TAA	*>*	hypothetical	syn
Mf|12	NC_006055:69934	C>T	IG				
Mf|55	NC_006055:70086	G>T	EX	TCT>TAT	S>Y	proline	nonsyn
Mf|57	NC_006055:70908	G>A	EX	AAG>AAA	K>K	preprotein	syn
Mf|55	NC_006055:73473	G>A	IG				
Mf|12	NC_006055:74006	C>T	EX	ACT>ATT	T>I	excinuclease	nonsyn

Mf|41	NC_006055:75261	A>G	EX	GAA>GAG	E>E	excinuclease	syn
Mf|93	NC_006055:76123	T>C	EX	GTT>GCT	V>A	repair	nonsyn
Mf|76	NC_006055:76468	C>T	EX	ACA>ATA	T>I	repair	nonsyn
Mf|76	NC_006055:77086	A>G	EX	CAT>CGT	H>R	repair	nonsyn
Mf|19	NC_006055:77821	G>T	EX	GGA>GTA	G>V	repair	nonsyn
Mf|11	NC_006055:80046	G>C	EX	TTG>TTC	L>F	transporter	nonsyn
Mf|49	NC_006055:83764	C>A	EX	GAC>GAA	D>E	prolipoprotein	nonsyn
Mf|58	NC_006055:89160	G>A	EX	GGA>AGA	G>R	substrate	nonsyn
Mf|65	NC_006055:91290	C>T	EX	CAA>TAA	Q>*	cytosol	nonsyn
Mf|57	NC_006055:95579	C>G	EX	GGC>GGG	G>G	adenylosuccinate	syn
Mf|55	NC_006055:95951	C>G	EX	GAC>GAG	D>E	adenylosuccinate	nonsyn
Mf|23	NC_006055:96815	G>A	EX	GAG>AAG	E>K	adenylosuccinate	nonsyn
Mf|6	NC_006055:96891	C>T	EX	GCA>GTA	A>V	adenylosuccinate	nonsyn
Mf|92	NC_006055:101397	C>T	EX	CTG>TTG	L>L	hypothetical	syn
Mf|90	NC_006055:101544	C>G	EX	CCT>GCT	P>A	hypothetical	nonsyn
Mf|49	NC_006055:101785	C>G	EX	TCA>TGA	S>*	hypothetical	nonsyn
Mf|68	NC_006055:102497	C>T	IG				
Mf|90	NC_006055:108570	C>G	EX	ACA>AGA	T>R	Glu-tRNA	nonsyn
Mf|99	NC_006055:108699	G>T	EX	TGT>TTT	C>F	Glu-tRNA	nonsyn
Mf|67	NC_006055:110775	C>T	EX	CTA>TTA	L>L	rRNA	syn
Mf|19	NC_006055:113671	G>T	EX	AAG>AAT	K>N	metallo-beta	nonsyn
Mf|14	NC_006055:114754	C>T	EX	AAC>AAT	N>N	oligopeptide	syn
Mf|56	NC_006055:116834	G>A	EX	GCT>ACT	A>T	oligopeptide	nonsyn
Mf|12	NC_006055:117679	C>G	EX	CTT>GTT	L>V	oligopeptide	nonsyn
Mf|56	NC_006055:118076	G>T	EX	AGA>ATA	R>I	oligopeptide	nonsyn
Mf|59	NC_006055:118160	C>A	EX	ACT>AAT	T>N	oligopeptide	nonsyn
Mf|47	NC_006055:119417	G>A	EX	GGT>AGT	G>S	oligopeptide	nonsyn
Mf|93	NC_006055:120593	C>A	EX	GCA>GAA	A>E	oligopeptide	nonsyn
Mf|90	NC_006055:124696	C>T	EX	ATC>ATT	I>I	DNA	syn
Mf|58	NC_006055:125978	C>T	EX	CCT>TCT	P>S	DNA	nonsyn
Mf|12	NC_006055:127441	G>A	IG				
Mf|19	NC_006055:127854	C>A	EX	CCA>CAA	P>Q	repair	nonsyn
Mf|76	NC_006055:128445	C>T	EX	ACA>ATA	T>I	repair	nonsyn
Mf|57	NC_006055:130118	G>T	EX	GCA>TCA	A>S	tetrapyrrole	nonsyn
Mf|83	NC_006055:131141	C>G	EX	GAA>CAA	E>Q	rhodanese-related	nonsyn
Mf|49	NC_006055:136038	G>A	EX	AGA>AAA	R>K	ATP	nonsyn
Mf|58	NC_006055:137967	G>A	EX	GGG>GAG	G>E	ATP	nonsyn
Mf|41	NC_006055:142455	G>A	EX	GAT>AAT	D>N	multidrug	nonsyn
Mf|58	NC_006055:144431	C>T	EX	ACT>ATT	T>I	phosphomannomutase	nonsyn
Mf|76	NC_006055:149859	C>T	IG				
Mf|12	NC_006055:151034	C>A	EX	TCT>TAT	S>Y	30S	nonsyn
Mf|49	NC_006055:151490	C>T	EX	AAC>AAT	N>N	50S	syn
Mf|56	NC_006055:152804	C>T	EX	GAC>GAT	D>D	50S	syn
Mf|23	NC_006055:152869	C>T	EX	TCA>TTA	S>L	50S	nonsyn
Mf|58	NC_006055:155006	C>T	EX	AAC>AAT	N>N	50S	syn
Mf|55	NC_006055:155078	A>G	EX	GTA>GTG	V>V	50S	syn
Mf|12	NC_006055:156509	G>C	EX	GGG>GGC	G>G	preprotein	syn
Mf|6	NC_006055:158125	C>G	EX	AAC>AAG	N>K	cytidine	nonsyn
Mf|19	NC_006055:159507	G>C	EX	GAT>CAT	D>H	methionine	nonsyn
Mf|39	NC_006055:159961	T>A	EX	ATT>AAT	I>N	50S	nonsyn
Mf|93	NC_006055:161490	C>A	EX	GCG>GAG	A>E	DNA-directed	nonsyn
Mf|58	NC_006055:161553	C>G	EX	GCT>GGT	A>G	DNA-directed	nonsyn
Mf|50	NC_006055:162564	C>A	EX	CAA>AAA	Q>K	cobalt	nonsyn
Mf|99	NC_006055:164938	C>A	EX	CCT>CAT	P>H	cobalt	nonsyn
Mf|68	NC_006055:167051	G>A	EX	GTT>ATT	V>I	substrate	nonsyn
Mf|39	NC_006055:167257	C>A	EX	ATC>ATA	I>I	substrate	syn
Mf|93	NC_006055:168584	G>C	EX	GTG>CTG	V>L	substrate	nonsyn
Mf|47	NC_006055:168715	G>A	EX	GCG>GCA	A>A	substrate	syn
Mf|58	NC_006055:169583	G>A	EX	GAT>AAT	D>N	substrate	nonsyn

Mf|58	NC_006055:171854	A>G	EX	TAT>TGT	Y>C	hypothetical	nonsyn
Mf|58	NC_006055:172565	C>T	EX	ACG>ATG	T>M	hypothetical	nonsyn
Mf|23	NC_006055:174352	G>A	EX	TCT>TTT	S>F	hypothetical	nonsyn
Mf|92	NC_006055:175428	G>A	EX	TTC>TTT	F>F	hypothetical	syn
Mf|19	NC_006055:181686	C>T	EX	TTC>TTT	F>F	DNA	syn
Mf|49	NC_006055:184687	G>C	EX	GTA>CTA	V>L	aspartyl/glutamyl-tRNA	nonsyn
Mf|58	NC_006055:190520	G>A	EX	ATG>ATA	M>I	methylenetetrahydrofolate	nonsyn
Mf|19	NC_006055:197380	A>G	EX	AAA>AAG	K>K	substrate	syn
Mf|58	NC_006055:198338	G>A	EX	GGG>AGG	G>R	substrate	nonsyn
Mf|99	NC_006055:200234	G>C	EX	GGA>GCA	G>A	guanosine	nonsyn
Mf|39	NC_006055:202514	G>T	EX	GAT>TAT	D>Y	dehydrogenase	nonsyn
Mf|62	NC_006055:203053	T>C	EX	GTA>GCA	V>A	6-phosphofructokinase	nonsyn
Mf|57	NC_006055:204548	G>A	EX	GAC>AAC	D>N	pyruvate	nonsyn
Mf|23	NC_006055:204753	C>G	EX	ACT>AGT	T>S	pyruvate	nonsyn
Mf|39	NC_006055:205232	G>A	EX	GTA>ATA	V>I	pyruvate	nonsyn
Mf|47	NC_006055:206061	G>A	IG				
Mf|39	NC_006055:210357	C>A	EX	CCT>ACT	P>T	fructose-specific	nonsyn
Mf|83	NC_006055:211515	G>C	EX	GTA>CTA	V>L	fructose-specific	nonsyn
Mf|42	NC_006055:211522	C>T	EX	GCC>GTC	A>V	fructose-specific	nonsyn
Mf|92	NC_006055:214957	G>T	EX	AGA>ATA	R>I	amino	nonsyn
Mf|49	NC_006055:216413	G>C	EX	CAC>CAG	H>Q	formate/nitrate	nonsyn
Mf|67	NC_006055:216967	C>A	IG				
Mf|6	NC_006055:218539	G>T	IG				
Mf|90	NC_006055:220338	C>A	EX	AGG>AGT	R>S	tryptophanyl-tRNA	nonsyn
Mf|76	NC_006055:221028	C>G	EX	CCA>CGA	P>R	NAD	nonsyn
Mf|59	NC_006055:224303	T>C	EX	CTA>CCA	L>P	hypothetical	nonsyn
Mf|90	NC_006055:224317	G>C	EX	GAA>CAA	E>Q	hypothetical	nonsyn
Mf|6	NC_006055:226327	A>T	EX	AAA>AAT	K>N	GTP-binding	nonsyn
Mf|6	NC_006055:228086	T>C	IG				
Mf|39	NC_006055:231335	G>A	EX	GAC>AAC	D>N	recombinase	nonsyn
Mf|85	NC_006055:235418	G>A	EX	GAA>AAA	E>K	virulence	nonsyn
Mf|14	NC_006055:235829	G>A	EX	GAA>AAA	E>K	virulence	nonsyn
Mf|62	NC_006055:236165	G>C	EX	GTA>CTA	V>L	virulence	nonsyn
Mf|65	NC_006055:237199	C>T	EX	TCT>TTT	S>F	hypothetical	nonsyn
Mf|58	NC_006055:237695	G>C	EX	GTG>GTC	V>V	hypothetical	syn
Mf|92	NC_006055:238301	C>T	EX	AAC>AAT	N>N	PTS	syn
Mf|65	NC_006055:242529	C>T	IG				
Mf|55	NC_006055:244653	G>C	EX	GAT>CAT	D>H	S-adenosylmethionine-dependent	nonsyn
Mf|50	NC_006055:245331	C>A	EX	TCA>TAA	S>*	hypothetical	nonsyn
Mf|47	NC_006055:246881	C>T	EX	ACA>ATA	T>I	serine/threonine	nonsyn
Mf|93	NC_006055:247256	G>C	EX	GGT>CGT	G>R	serine/threonine	nonsyn
Mf|26	NC_006055:247457	G>T	EX	GAA>TAA	E>*	serine/threonine	nonsyn
Mf|14	NC_006055:247924	C>T	EX	GAC>GAT	D>D	serine/threonine	syn
Mf|56	NC_006055:248189	G>A	EX	GAA>AAA	E>K	serine/threonine	nonsyn
Mf|59	NC_006055:250037	G>T	EX	GAA>TAA	E>*	hypothetical	nonsyn
Mf|76	NC_006055:251290	G>C	EX	TGT>TCT	C>S	hypothetical	nonsyn
Mf|26	NC_006055:251317	C>A	EX	GCT>GAT	A>D	hypothetical	nonsyn
Mf|58	NC_006055:254026	G>A	EX	GCA>ACA	A>T	dihydroxyacetone	nonsyn
Mf|92	NC_006055:256804	G>A	EX	GAT>AAT	D>N	structural	nonsyn
Mf|56	NC_006055:262342	T>C	EX	TAC>CAC	Y>H	phosphate	nonsyn
Mf|65	NC_006055:263612	G>T	EX	GAA>TAA	E>*	phosphate	nonsyn
Mf|93	NC_006055:264210	A>C	IG				
Mf|56	NC_006055:268748	C>A	EX	CGT>AGT	R>S	membrane	nonsyn
Mf|67	NC_006055:269086	C>T	EX	TAC>TAT	Y>Y	membrane	syn
Mf|58	NC_006055:269855	C>T	EX	AAC>AAT	N>N	tRNA	syn
Mf|50	NC_006055:270629	T>C	EX	CCT>CCC	P>P	tRNA	syn
Mf|50	NC_006055:271955	C>G	EX	GCT>GGT	A>G	cmp-binding	nonsyn
Mf|6	NC_006055:275005	C>T	EX	GAA>AAA	E>K	hypothetical	nonsyn
Mf|85	NC_006055:276438	C>A	EX	GTA>TTA	V>L	hypothetical	nonsyn

Mf|76	NC_006055:277561	A>T	EX	GAT>GAA	D>E	hypothetical	nonsyn
Mf|58	NC_006055:281533	G>A	EX	GAT>AAT	D>N	aldo/keto	nonsyn
Mf|99	NC_006055:284867	G>T	EX	GGT>TGT	G>C	multidrug	nonsyn
Mf|68	NC_006055:289583	G>A	EX	GTT>ATT	V>I	hypothetical	nonsyn
Mf|90	NC_006055:294286	T>G	EX	CTT>CTG	L>L	substrate	syn
Mf|42	NC_006055:295358	G>T	EX	GAT>TAT	D>Y	substrate	nonsyn
Mf|58	NC_006055:297174	G>A	EX	GTA>ATA	V>I	substrate	nonsyn
Mf|49	NC_006055:297716	G>T	EX	GCG>GCT	A>A	substrate	syn
Mf|49	NC_006055:303632	A>G	EX	TGA>TGG	*>W	multidrug	nonsyn
Mf|85	NC_006055:304618	G>A	EX	GGT>AGT	G>S	metal-dependent	nonsyn
Mf|50	NC_006055:306493	G>C	IG				
Mf|49	NC_006055:308466	G>C	EX	GTT>CTT	V>L	DNA	nonsyn
Mf|19	NC_006055:310242	C>T	EX	TAC>TAT	Y>Y	RNA	syn
Mf|59	NC_006055:311071	C>A	EX	CAT>AAT	H>N	RNA	nonsyn
Mf|67	NC_006055:311501	G>A	EX	GAT>AAT	D>N	SAM-dependent	nonsyn
Mf|12	NC_006055:311734	C>T	EX	AAC>AAT	N>N	SAM-dependent	syn
Mf|93	NC_006055:314539	G>A	EX	GGA>GAA	G>E	hypothetical	nonsyn
Mf|99	NC_006055:315564	G>C	EX	GAA>CAA	E>Q	hypothetical	nonsyn
Mf|41	NC_006055:316795	G>T	EX	GAG>GAT	E>D	protein-export	nonsyn
Mf|65	NC_006055:319587	G>A	EX	GGA>GAA	G>E	protein-export	nonsyn
Mf|57	NC_006055:324024	C>T	EX	CAC>CAT	H>H	hypothetical	syn
Mf|19	NC_006055:324141	G>C	EX	ATG>ATC	M>I	hypothetical	nonsyn
Mf|19	NC_006055:327322	C>T	EX	GCA>GTA	A>V	hypothetical	nonsyn
Mf|57	NC_006055:330221	C>T	EX	GTC>GTT	V>V	DNA	syn
Mf|68	NC_006055:331452	G>A	EX	GAC>AAC	D>N	DNA	nonsyn
Mf|39	NC_006055:334556	C>T	EX	ACA>ATA	T>I	DAD(P)H	nonsyn
Mf|50	NC_006055:336440	T>C	EX	ATT>ACT	I>T	hypothetical	nonsyn
Mf|55	NC_006055:339010	A>G	EX	TAT>TGT	Y>C	translation	nonsyn
Mf|85	NC_006055:341167	T>A	EX	TTT>ATT	F>I	hypothetical	nonsyn
Mf|11	NC_006055:341564	C>A	EX	CCA>CAA	P>Q	hypothetical	nonsyn
Mf|41	NC_006055:348869	C>T	EX	TTC>TTT	F>F	deoxycytosine	syn
Mf|90	NC_006055:349980	G>C	EX	GCA>CCA	A>P	deoxycytosine	nonsyn
Mf|92	NC_006055:350801	C>T	EX	ACA>ATA	T>I	hypothetical	nonsyn
Mf|12	NC_006055:351344	G>T	EX	AGA>ATA	R>I	hypothetical	nonsyn
Mf|65	NC_006055:353519	A>T	EX	AAT>AAA	N>K	type	nonsyn
Mf|6	NC_006055:356160	G>A	EX	GGT>GAT	G>D	DNA	nonsyn
Mf|26	NC_006055:358919	G>A	EX	GAA>AAA	E>K	DNA	nonsyn
Mf|50	NC_006055:362129	C>T	EX	CTA>TTA	L>L	beta-glucoside	syn
Mf|12	NC_006055:362363	G>T	EX	GTT>TTT	V>F	beta-glucoside	nonsyn
Mf|57	NC_006055:366801	G>A	EX	GCT>ACT	A>T	DNA	nonsyn
Mf|65	NC_006055:369017	G>A	EX	GCT>ACT	A>T	lysophospholipase	nonsyn
Mf|58	NC_006055:369212	G>A	EX	GAT>AAT	D>N	lysophospholipase	nonsyn
Mf|76	NC_006055:371938	G>A	EX	TTG>TTA	L>L	alpha	syn
Mf|76	NC_006055:373065	G>T	EX	GAA>TAA	E>*	beta-glucoside	nonsyn
Mf|49	NC_006055:375644	G>T	EX	CAT>AAT	H>N	hypothetical	nonsyn
Mf|62	NC_006055:378647	C>T	EX	TCT>TTT	S>F	exodeoxyribonuclease	nonsyn
Mf|57	NC_006055:380197	G>T	EX	TCA>TAA	S>*	hypothetical	nonsyn
Mf|68	NC_006055:380440	G>A	EX	TCA>TTA	S>L	hypothetical	nonsyn
Mf|58	NC_006055:383797	G>T	EX	TCA>TAA	S>*	hypothetical	nonsyn
Mf|41	NC_006055:387466	G>T	EX	TCA>TAA	S>*	rRNA	nonsyn
Mf|55	NC_006055:389221	C>A	EX	AGA>ATA	R>I	exodeoxyribonuclease	nonsyn
Mf|58	NC_006055:390543	G>T	EX	TCA>TAA	S>*	endonuclease	nonsyn
Mf|65	NC_006055:390670	C>T	EX	GCA>ACA	A>T	endonuclease	nonsyn
Mf|57	NC_006055:391566	G>T	EX	GCT>GAT	A>D	riboflavin	nonsyn
Mf|19	NC_006055:394773	C>A	EX	GTG>TTG	V>L	hypothetical	nonsyn
Mf|12	NC_006055:394872	C>T	EX	GAT>AAT	D>N	hypothetical	nonsyn
Mf|11	NC_006055:395189	G>A	EX	TCA>TTA	S>L	hypothetical	nonsyn
Mf|23	NC_006055:397049	A>T	IG				
Mf|23	NC_006055:397601	G>A	EX	GCA>ACA	A>T	GMP	nonsyn

Mf|6	NC_006055:397953	C>T	EX	GCT>GTT	A>V	GMP	nonsyn
Mf|83	NC_006055:401473	G>C	EX	TTC>TTG	F>L	chitinase	nonsyn
Mf|67	NC_006055:401825	G>T	EX	CCT>CAT	P>H	chitinase	nonsyn
Mf|42	NC_006055:402425	C>A	EX	GGA>GTA	G>V	chitinase	nonsyn
Mf|99	NC_006055:403449	A>T	EX	TCA>ACA	S>T	chitinase	nonsyn
Mf|99	NC_006055:403450	A>T	EX	GTT>GTA	V>V	chitinase	syn
Mf|99	NC_006055:403455	A>T	EX	TAT>AAT	Y>N	chitinase	nonsyn
Mf|62	NC_006055:409021	G>A	EX	CAA>TAA	Q>*	hypothetical	nonsyn
Mf|92	NC_006055:410584	A>G	EX	TTA>CTA	L>L	chromosome	syn
Mf|92	NC_006055:410585	G>A	EX	TTC>TTT	F>F	chromosome	syn
Mf|57	NC_006055:411195	G>A	EX	TCA>TTA	S>L	chromosome	nonsyn
Mf|11	NC_006055:414792	G>A	EX	TAC>TAT	Y>Y	valyl-tRNA	syn
Mf|14	NC_006055:416659	C>A	EX	GAT>TAT	D>Y	Mg2+	nonsyn
Mf|19	NC_006055:421247	C>A	EX	TCA>TAA	S>*	hypothetical	nonsyn
Mf|65	NC_006055:422425	G>A	EX	CAA>TAA	Q>*	30S	nonsyn
Mf|90	NC_006055:422899	T>G	EX	TTA>TTC	L>F	hypothetical	nonsyn
Mf|19	NC_006055:426085	G>A	EX	ACA>ATA	T>I	histidyl-tRNA	nonsyn
Mf|42	NC_006055:427320	G>A	EX	CTA>TTA	L>L	hypothetical	syn
Mf|49	NC_006055:429160	G>T	EX	CAT>AAT	H>N	hypothetical	nonsyn
Mf|55	NC_006055:429611	C>G	EX	ACA>AGA	T>R	30S	nonsyn
Mf|62	NC_006055:429615	G>A	EX	GCG>GCA	A>A	30S	syn
Mf|58	NC_006055:429882	G>A	EX	CAA>TAA	Q>*	DNA	nonsyn
Mf|59	NC_006055:431586	G>A	EX	CAA>TAA	Q>*	DNA	nonsyn
Mf|41	NC_006055:432601	A>G	EX	ATT>ACT	I>T	ribosome-binding	nonsyn
Mf|58	NC_006055:434827	C>T	EX	TGA>TAA	*>*	rRNA	syn
Mf|62	NC_006055:435114	C>A	EX	GAT>TAT	D>Y	xanthosine	nonsyn
Mf|65	NC_006055:435845	T>G	EX	TTT>TTG	F>L	choline	nonsyn
Mf|19	NC_006055:439968	G>A	IG				
Mf|49	NC_006055:441361	G>T	EX	GGA>TGA	G>*	hypothetical	nonsyn
Mf|83	NC_006055:441941	C>T	IG				
Mf|26	NC_006055:442143	T>C	EX	CAT>CGT	H>R	1-acyl-sn-glycerol-3-phosphate	nonsyn
Mf|41	NC_006055:444902	G>A	EX	TCA>TTA	S>L	hypothetical	nonsyn
Mf|59	NC_006055:444959	G>A	EX	CCA>CTA	P>L	hypothetical	nonsyn
Mf|47	NC_006055:447308	C>G	EX	GGA>CGA	G>R	lipoprotein	nonsyn
Mf|93	NC_006055:447393	C>A	EX	ATG>ATT	M>I	lipoprotein	nonsyn
Mf|58	NC_006055:448432	C>T	EX	TTG>TTA	L>L	isoleucyl-tRNA	syn
Mf|93	NC_006055:449769	C>A	EX	GTT>TTT	V>F	isoleucyl-tRNA	nonsyn
Mf|76	NC_006055:449805	G>T	EX	CGT>AGT	R>S	isoleucyl-tRNA	nonsyn
Mf|58	NC_006055:449909	G>A	EX	ACA>ATA	T>I	isoleucyl-tRNA	nonsyn
Mf|39	NC_006055:451989	G>C	EX	TCC>TCG	S>S	hypothetical	syn
Mf|6	NC_006055:452222	G>A	EX	GCT>GTT	A>V	cell	nonsyn
Mf|26	NC_006055:452235	C>A	EX	GTA>TTA	V>L	cell	nonsyn
Mf|12	NC_006055:452331	C>T	EX	GTT>ATT	V>I	cell	nonsyn
Mf|68	NC_006055:452334	C>T	EX	GTT>ATT	V>I	cell	nonsyn
Mf|26	NC_006055:452799	G>A	EX	CAA>TAA	Q>*	cell	nonsyn
Mf|49	NC_006055:452856	C>G	EX	GCT>CCT	A>P	cell	nonsyn
Mf|12	NC_006055:453422	A>G	EX	TAT>CAT	Y>H	hypothetical	nonsyn
Mf|55	NC_006055:453756	C>T	EX	ATG>ATA	M>I	hypothetical	nonsyn
Mf|23	NC_006055:454220	G>A	EX	CCA>TCA	P>S	hypothetical	nonsyn
Mf|58	NC_006055:454382	C>T	EX	GAA>AAA	E>K	hypothetical	nonsyn
Mf|65	NC_006055:454496	C>A	IG				
Mf|39	NC_006055:454536	G>A	EX	AGC>AGT	S>S	S-adenosyl-methyltransferase	syn
Mf|42	NC_006055:455013	G>T	EX	AAC>AAA	N>K	S-adenosyl-methyltransferase	nonsyn
Mf|65	NC_006055:455464	C>A	EX	GCT>TCT	A>S	cell	nonsyn
Mf|68	NC_006055:456315	G>A	EX	ACT>ATT	T>I	hypothetical	nonsyn
Mf|62	NC_006055:461113	C>A	EX	GAA>TAA	E>*	substrate	nonsyn
Mf|92	NC_006055:463957	C>A	EX	GCA>TCA	A>S	substrate	nonsyn
Mf|99	NC_006055:472448	C>T	EX	GCT>ACT	A>T	class	nonsyn
Mf|68	NC_006055:472456	C>A	EX	GGA>GTA	G>V	class	nonsyn

Mf|19	NC_006055:472648	A>G	EX	ATT>ACT	I>T	class	nonsyn
Mf|11	NC_006055:473147	C>A	EX	GAA>TAA	E>*	class	nonsyn
Mf|26	NC_006055:473264	G>T	EX	CAA>AAA	Q>K	class	nonsyn
Mf|6	NC_006055:473516	G>T	EX	CAT>AAT	H>N	class	nonsyn
Mf|62	NC_006055:473578	C>G	EX	TGA>TCA	*>S	class	nonsyn
Mf|85	NC_006055:473693	C>A	EX	GAA>TAA	E>*	class	nonsyn
Mf|41	NC_006055:474371	G>C	EX	CAG>GAG	Q>E	class	nonsyn
Mf|76	NC_006055:474442	C>T	EX	GGA>GAA	G>E	class	nonsyn
Mf|19	NC_006055:476970	G>T	EX	CAA>AAA	Q>K	hypothetical	nonsyn
Mf|11	NC_006055:477017	G>A	EX	TCA>TTA	S>L	hypothetical	nonsyn
Mf|76	NC_006055:480986	A>C	EX	TTT>TTG	F>L	hypothetical	nonsyn
Mf|93	NC_006055:482305	C>A	EX	GCT>TCT	A>S	tRNA-specific	nonsyn
Mf|50	NC_006055:482711	G>T	EX	CAA>AAA	Q>K	xanthine/uracil	nonsyn
Mf|90	NC_006055:486103	G>A	EX	GCG>GTG	A>V	molecular	nonsyn
Mf|19	NC_006055:486245	C>T	EX	GAA>AAA	E>K	molecular	nonsyn
Mf|90	NC_006055:488795	G>T	IG				
Mf|42	NC_006055:490193	G>A	EX	ACT>ATT	T>I	ATPase	nonsyn
Mf|47	NC_006055:490514	G>A	EX	TCA>TTA	S>L	ATPase	nonsyn
Mf|76	NC_006055:493045	C>T	EX	CTG>CTA	L>L	holliday	syn
Mf|49	NC_006055:495182	C>A	EX	TGA>TTA	*>L	multidrug	nonsyn
Mf|85	NC_006055:498717	T>C	EX	TTC>TCC	F>S	beta-glucosidase	nonsyn
Mf|47	NC_006055:499319	G>A	EX	GTT>ATT	V>I	beta-glucosidase	nonsyn
Mf|59	NC_006055:500526	C>G	EX	GCA>GGA	A>G	trehalose/sucrose/beta-glucoside	nonsyn
Mf|57	NC_006055:504931	T>G	EX	ATC>CTC	I>L	hypothetical	nonsyn
Mf|83	NC_006055:507222	G>C	EX	ACA>AGA	T>R	hypothetical	nonsyn
Mf|58	NC_006055:508938	C>G	EX	TTG>TTC	L>F	6-phospho-beta-glucosidase	nonsyn
Mf|56	NC_006055:510689	A>G	EX	TAC>CAC	Y>H	transcriptional	nonsyn
Mf|39	NC_006055:511628	C>A	EX	GGA>TGA	G>*	hypothetical	nonsyn
Mf|90	NC_006055:512062	G>T	EX	ATG>ATT	M>I	hypothetical	nonsyn
Mf|58	NC_006055:514705	G>A	EX	GCT>GTT	A>V	arginine	nonsyn
Mf|58	NC_006055:517256	C>T	IG				
Mf|6	NC_006055:518237	A>G	EX	TTA>TCA	L>S	hypothetical	nonsyn
Mf|90	NC_006055:521293	C>A	EX	GAT>TAT	D>Y	membrane-associated	nonsyn
Mf|55	NC_006055:523043	C>A	IG				
Mf|12	NC_006055:523496	G>T	IG				
Mf|58	NC_006055:523739	A>G	IG				
Mf|76	NC_006055:524223	G>T	IG				
Mf|58	NC_006055:524328	G>T	IG				
Mf|50	NC_006055:525001	C>T	EX	ATG>ATA	M>I	membrane-associated	nonsyn
Mf|47	NC_006055:526392	C>T	EX	GAT>AAT	D>N	membrane-associated	nonsyn
Mf|6	NC_006055:526610	C>T	EX	GGA>GAA	G>E	membrane-associated	nonsyn
Mf|92	NC_006055:527046	C>T	EX	GAA>AAA	E>K	membrane-associated	nonsyn
Mf|76	NC_006055:531159	T>G	IG				
Mf|58	NC_006055:531646	G>A	EX	TCA>TTA	S>L	hypothetical	nonsyn
Mf|23	NC_006055:532517	A>C	EX	GTT>GTG	V>V	multidrug	syn
Mf|11	NC_006055:534758	G>A	IG				
Mf|47	NC_006055:536946	G>A	EX	ACA>ATA	T>I	histone-like	nonsyn
Mf|19	NC_006055:537926	T>A	EX	TTA>TTT	L>F	histone-like	nonsyn
Mf|58	NC_006055:538407	G>A	EX	ACT>ATT	T>I	hypothetical	nonsyn
Mf|59	NC_006055:539559	G>A	EX	ACT>ATT	T>I	hypothetical	nonsyn
Mf|49	NC_006055:540304	C>T	EX	GGC>GAC	G>D	hypothetical	nonsyn
Mf|58	NC_006055:540340	G>A	EX	ACA>ATA	T>I	hypothetical	nonsyn
Mf|41	NC_006055:541068	G>C	EX	TAC>TAG	Y>*	hypothetical	nonsyn
Mf|59	NC_006055:541640	C>A	EX	GGT>TGT	G>C	hypothetical	nonsyn
Mf|39	NC_006055:544108	G>T	EX	TCA>TAA	S>*	hypothetical	nonsyn
Mf|12	NC_006055:545977	G>A	EX	GCA>GTA	A>V	multidrug	nonsyn
Mf|19	NC_006055:546911	C>T	EX	GAA>AAA	E>K	multidrug	nonsyn
Mf|65	NC_006055:547934	C>T	EX	GTA>ATA	V>I	multidrug	nonsyn
Mf|93	NC_006055:548212	G>T	EX	TCA>TAA	S>*	multidrug	nonsyn

Mf|99	NC_006055:550696	C>T	EX	GTG>GTA	V>V	phosphatidylglycerophosphatase	syn
Mf|26	NC_006055:554350	C>A	EX	TTC>TTA	F>L	GTP-binding	nonsyn
Mf|57	NC_006055:555265	C>A	IG				
Mf|49	NC_006055:555848	C>T	EX	GTT>ATT	V>I	nucleoside	nonsyn
Mf|93	NC_006055:558669	C>A	EX	GGG>GTG	G>V	GTP-binding	nonsyn
Mf|68	NC_006055:559862	C>T	IG				
Mf|49	NC_006055:560650	T>G	EX	ATT>CTT	I>L	prolyl-tRNA	nonsyn
Mf|12	NC_006055:561732	T>C	EX	TAC>TGC	Y>C	prolyl-tRNA	nonsyn
Mf|62	NC_006055:561991	C>T	EX	GGA>GAA	G>E	hypothetical	nonsyn
Mf|67	NC_006055:565625	C>T	EX	GTT>ATT	V>I	asparaginyl-tRNA	nonsyn
Mf|47	NC_006055:565949	C>A	EX	GAC>TAC	D>Y	asparaginyl-tRNA	nonsyn
Mf|62	NC_006055:567517	C>T	EX	GTA>ATA	V>I	lipase	nonsyn
Mf|41	NC_006055:569317	G>T	EX	CCT>ACT	P>T	DNA-binding/iron	nonsyn
Mf|58	NC_006055:570305	C>T	EX	GAG>GAA	E>E	hypothetical	syn
Mf|67	NC_006055:570997	C>T	EX	GCA>ACA	A>T	hypothetical	nonsyn
Mf|47	NC_006055:572809	C>G	EX	GTA>CTA	V>L	hypothetical	nonsyn
Mf|6	NC_006055:573092	G>A	EX	AAC>AAT	N>N	hypothetical	syn
Mf|39	NC_006055:573709	C>T	EX	GCA>ACA	A>T	hypothetical	nonsyn
Mf|47	NC_006055:575847	G>C	EX	TCA>TGA	S>*	leucyl-tRNA	nonsyn
Mf|67	NC_006055:575852	A>G	EX	GGT>GGC	G>G	leucyl-tRNA	syn
Mf|56	NC_006055:578464	C>T	EX	ATG>ATA	M>I	hypothetical	nonsyn
Mf|39	NC_006055:578965	C>T	EX	GGA>AGA	G>R	30S	nonsyn
Mf|85	NC_006055:580964	G>A	EX	CCA>TCA	P>S	Mg2+	nonsyn
Mf|58	NC_006055:581065	G>A	EX	ACA>ATA	T>I	Mg2+	nonsyn
Mf|14	NC_006055:581368	T>C	EX	GAA>GGA	E>G	Mg2+	nonsyn
Mf|26	NC_006055:586812	A>G	EX	GGT>GGC	G>G	trehalose	syn
Mf|57	NC_006055:588663	T>G	EX	CAA>CCA	Q>P	trehalose	nonsyn
Mf|76	NC_006055:592003	G>C	EX	GCT>GGT	A>G	triosephosphate	nonsyn
Mf|47	NC_006055:593026	T>C	EX	GAT>GGT	D>G	hypothetical	nonsyn
Mf|99	NC_006055:593229	G>C	EX	AAC>AAG	N>K	hypothetical	nonsyn
Mf|42	NC_006055:593553	C>A	EX	ATG>ATT	M>I	hypothetical	nonsyn
Mf|93	NC_006055:593868	C>A	EX	AAG>AAT	K>N	hypothetical	nonsyn
Mf|83	NC_006055:596599	C>T	IG				
Mf|6	NC_006055:598141	G>A	EX	CCT>CTT	P>L	hypothetical	nonsyn
Mf|59	NC_006055:602385	C>G	EX	GTG>CTG	V>L	spermidine/putrescine	nonsyn
Mf|85	NC_006055:603323	G>C	EX	CAC>CAG	H>Q	spermidine/putrescine	nonsyn
Mf|67	NC_006055:604570	G>T	IG				
Mf|58	NC_006055:605201	C>T	EX	GGT>AGT	G>S	HAD-superfamily	nonsyn
Mf|55	NC_006055:610126	C>T	EX	GGT>AGT	G>S	sucrose	nonsyn
Mf|47	NC_006055:610520	A>C	IG				
Mf|85	NC_006055:611082	C>T	IG				
Mf|55	NC_006055:611205	C>A	EX	ATG>ATT	M>I	phosphoenolpyruvate-protein	nonsyn
Mf|99	NC_006055:613403	C>A	EX	GGT>TGT	G>C	NH3-dependent	nonsyn
Mf|23	NC_006055:613576	G>T	EX	TCT>TAT	S>Y	NH3-dependent	nonsyn
Mf|59	NC_006055:613676	G>A	EX	CCG>TCG	P>S	NH3-dependent	nonsyn
Mf|92	NC_006055:616893	A>G	EX	CGT>CGC	R>R	ATP-dependent	syn
Mf|50	NC_006055:617221	T>C	EX	AAT>AGT	N>S	ATP-dependent	nonsyn
Mf|50	NC_006055:617222	T>C	EX	AAT>GAT	N>D	ATP-dependent	nonsyn
Mf|56	NC_006055:617305	C>A	EX	AGA>ATA	R>I	ATP-dependent	nonsyn
Mf|93	NC_006055:617507	C>T	EX	GTT>ATT	V>I	ATP-dependent	nonsyn
Mf|23	NC_006055:619728	G>A	EX	GCA>GTA	A>V	sucrose-6-phosphate	nonsyn
Mf|58	NC_006055:620021	T>A	EX	ACA>ACT	T>T	sucrose-6-phosphate	syn
Mf|49	NC_006055:620657	C>T	EX	GTT>ATT	V>I	sucrose	nonsyn
Mf|83	NC_006055:621365	C>G	EX	GAT>CAT	D>H	sucrose	nonsyn
Mf|99	NC_006055:623813	G>A	EX	GCT>GTT	A>V	ribonucleotide-diphosphate	nonsyn
Mf|56	NC_006055:624419	C>A	EX	GAA>TAA	E>*	ribonucleotide-diphosphate	nonsyn
Mf|90	NC_006055:626728	T>C	EX	GAC>GGC	D>G	hypothetical	nonsyn
Mf|59	NC_006055:629205	C>T	EX	GGA>AGA	G>R	multidrug	nonsyn
Mf|68	NC_006055:629220	A>T	EX	TAC>AAC	Y>N	multidrug	nonsyn

Mf|83	NC_006055:629327	G>A	EX	ACA>ATA	T>I	multidrug	nonsyn
Mf|67	NC_006055:629955	C>T	EX	GTG>ATG	V>M	multidrug	nonsyn
Mf|58	NC_006055:630138	C>A	EX	GAA>TAA	E>*	multidrug	nonsyn
Mf|93	NC_006055:630486	G>A	EX	CAA>TAA	Q>*	multidrug	nonsyn
Mf|39	NC_006055:631318	G>A	EX	CAT>TAT	H>Y	multidrug/protein/lipid	nonsyn
Mf|58	NC_006055:631682	T>A	EX	ACA>ACT	T>T	multidrug/protein/lipid	syn
Mf|67	NC_006055:632146	G>C	EX	CAA>GAA	Q>E	multidrug/protein/lipid	nonsyn
Mf|58	NC_006055:632239	T>C	EX	ACT>GCT	T>A	multidrug/protein/lipid	nonsyn
Mf|67	NC_006055:633492	G>A	EX	TCA>TTA	S>L	single	nonsyn
Mf|19	NC_006055:633813	C>G	EX	GAA>CAA	E>Q	ribonuclease	nonsyn
Mf|59	NC_006055:635956	C>T	EX	AGG>AAG	R>K	tRNA	nonsyn
Mf|39	NC_006055:637099	C>A	EX	GCA>TCA	A>S	30S	nonsyn
Mf|99	NC_006055:637799	C>A	EX	ATG>ATT	M>I	hypothetical	nonsyn
Mf|55	NC_006055:638455	T>C	EX	TCT>TCC	S>S	inorganic	syn
Mf|23	NC_006055:639320	T>C	EX	AAC>AGC	N>S	hypothetical	nonsyn
Mf|67	NC_006055:640546	G>A	EX	GCT>GTT	A>V	hypothetical	nonsyn
Mf|58	NC_006055:640753	G>C	EX	GAC>GAG	D>E	deoxynucleoside	nonsyn
Mf|83	NC_006055:641330	A>G	EX	GAT>GAC	D>D	23S	syn
Mf|14	NC_006055:643248	G>A	EX	GCA>GTA	A>V	chromosomal	nonsyn
Mf|99	NC_006055:644453	G>C	EX	GAC>GAG	D>E	hypothetical	nonsyn
Mf|56	NC_006055:645436	C>A	EX	GTA>TTA	V>L	hypothetical	nonsyn
Mf|92	NC_006055:647355	G>T	EX	TTC>TTA	F>L	arginyl-tRNA	nonsyn
Mf|92	NC_006055:648499	G>C	EX	TCA>TGA	S>*	ribosome	nonsyn
Mf|85	NC_006055:650459	G>T	EX	CAT>AAT	H>N	chitin	nonsyn
Mf|99	NC_006055:650879	G>A	EX	GCT>GTT	A>V	hypothetical	nonsyn
Mf|90	NC_006055:651395	C>A	EX	GAT>TAT	D>Y	elongation	nonsyn
Mf|14	NC_006055:652274	C>A	EX	GTA>TTA	V>L	30S	nonsyn
Mf|41	NC_006055:652370	C>T	EX	GTA>ATA	V>I	30S	nonsyn
Mf|39	NC_006055:653542	G>A	EX	TCA>TTA	S>L	transporter	nonsyn
Mf|56	NC_006055:656145	G>C	EX	GCA>GGA	A>G	CAAX	nonsyn
Mf|12	NC_006055:657159	G>A	EX	GCT>GTT	A>V	phosphotransferase	nonsyn
Mf|93	NC_006055:662017	C>A	EX	TCT>TAT	S>Y	polypeptide	nonsyn
Mf|26	NC_006055:663656	C>A	EX	TCT>TAT	S>Y	metallo-beta	nonsyn
Mf|12	NC_006055:663721	G>T	EX	GGT>TGT	G>C	metallo-beta	nonsyn
Mf|99	NC_006055:667602	C>T	EX	GAA>AAA	E>K	hypothetical	nonsyn
Mf|76	NC_006055:668351	G>T	EX	TCA>TAA	S>*	hypothetical	nonsyn
Mf|99	NC_006055:669668	C>A	EX	TCA>TAA	S>*	hypothetical	nonsyn
Mf|26	NC_006055:670788	C>T	IG				
Mf|23	NC_006055:672603	C>A	EX	GAT>TAT	D>Y	phosphoglycerate	nonsyn
Mf|90	NC_006055:679986	T>C	EX	AAC>AGC	N>S	DNA	nonsyn
Mf|76	NC_006055:684844	C>A	EX	AGT>ATT	S>I	chromosome	nonsyn
Mf|85	NC_006055:690508	G>A	EX	GTT>ATT	V>I	fatty	nonsyn
Mf|50	NC_006055:690969	G>C	EX	GAT>CAT	D>H	fatty	nonsyn
Mf|68	NC_006055:691383	G>A	EX	GAA>AAA	E>K	fatty	nonsyn
Mf|83	NC_006055:691534	G>C	EX	AGA>ACA	R>T	fatty	nonsyn
Mf|11	NC_006055:691708	T>C	IG				
Mf|19	NC_006055:692933	G>C	EX	ACA>AGA	T>R	hypothetical	nonsyn
Mf|14	NC_006055:695941	C>T	EX	GGA>AGA	G>R	DNA-directed	nonsyn
Mf|93	NC_006055:698824	C>T	EX	GTT>ATT	V>I	DNA-directed	nonsyn
Mf|65	NC_006055:699544	C>T	EX	GGA>AGA	G>R	DNA-directed	nonsyn
Mf|26	NC_006055:700578	T>C	EX	AAC>AGC	N>S	DNA-directed	nonsyn
Mf|90	NC_006055:700618	C>G	EX	GTT>CTT	V>L	DNA-directed	nonsyn
Mf|50	NC_006055:700747	G>T	EX	CCT>ACT	P>T	DNA-directed	nonsyn
Mf|23	NC_006055:701907	C>T	EX	GGA>GAA	G>E	DNA-directed	nonsyn
Mf|23	NC_006055:703407	G>A	EX	CAA>TAA	Q>*	hypothetical	nonsyn
Mf|99	NC_006055:705464	G>T	IG				
Mf|55	NC_006055:705838	G>T	IG				
Mf|58	NC_006055:707246	C>T	EX	GTA>ATA	V>I	ABC	nonsyn
Mf|23	NC_006055:708334	G>A	EX	ACA>ATA	T>I	ABC	nonsyn

Mf|6	NC_006055:712338	T>C	EX	AAC>AGC	N>S	50S	nonsyn
Mf|76	NC_006055:712955	G>A	EX	GCT>GTT	A>V	50S	nonsyn
Mf|26	NC_006055:713653	C>A	IG				
Mf|41	NC_006055:714052	T>C	EX	GTG>GCG	V>A	hypothetical	nonsyn
Mf|59	NC_006055:714565	G>A	EX	GGG>GAG	G>E	hypothetical	nonsyn
Mf|47	NC_006055:715274	G>T	EX	CCA>CAA	P>Q	hypothetical	nonsyn
Mf|42	NC_006055:720375	G>A	EX	TCA>TTA	S>L	beta-glucosidase	nonsyn
Mf|59	NC_006055:720594	G>A	EX	TCA>TTA	S>L	beta-glucosidase	nonsyn
Mf|68	NC_006055:721662	T>C	EX	ATT>GTT	I>V	transcriptional	nonsyn
Mf|19	NC_006055:723109	C>T	EX	GTT>ATT	V>I	6-phospho-beta-glucosidase	nonsyn
Mf|62	NC_006055:723466	C>G	EX	GTT>CTT	V>L	6-phospho-beta-glucosidase	nonsyn
Mf|65	NC_006055:724610	G>A	EX	ACA>ATA	T>I	transcriptional	nonsyn
Mf|19	NC_006055:727128	C>A	EX	GGG>GGT	G>G	substrate	syn
Mf|26	NC_006055:727328	C>T	EX	GTG>ATG	V>M	substrate	nonsyn
Mf|58	NC_006055:728007	G>A	EX	CCA>CTA	P>L	elongation	nonsyn
Mf|99	NC_006055:731438	G>A	EX	AAC>AAT	N>N	30S	syn
Mf|67	NC_006055:732133	C>T	EX	GCG>GCA	A>A	30S	syn
Mf|58	NC_006055:733804	G>A	EX	CCA>CTA	P>L	cardiolipin	nonsyn
Mf|11	NC_006055:736487	C>A	EX	GAA>TAA	E>*	hypothetical	nonsyn
Mf|83	NC_006055:738077	G>A	EX	CTT>TTT	L>F	polypeptide	nonsyn
Mf|58	NC_006055:738229	G>A	EX	TCA>TTA	S>L	polypeptide	nonsyn
Mf|58	NC_006055:739931	C>T	EX	GCC>ACC	A>T	peptide	nonsyn
Mf|49	NC_006055:744513	G>T	IG				
Mf|85	NC_006055:747858	C>T	EX	GCG>GCA	A>A	ribokinase	syn
Mf|76	NC_006055:749091	C>T	EX	GAT>AAT	D>N	PTS	nonsyn
Mf|23	NC_006055:749659	T>C	IG				
Mf|85	NC_006055:751595	G>T	EX	GCT>GAT	A>D	multidrug	nonsyn
Mf|62	NC_006055:751842	G>A	EX	CAA>TAA	Q>*	multidrug	nonsyn
Mf|65	NC_006055:752332	C>G	IG				
Mf|93	NC_006055:755692	C>T	EX	ATG>ATA	M>I	HD	nonsyn
Mf|6	NC_006055:758000	C>T	EX	GTT>ATT	V>I	sodium-glutamate	nonsyn
Mf|99	NC_006055:759951	G>T	EX	AAC>AAA	N>K	NADH	nonsyn
Mf|85	NC_006055:760327	G>A	EX	ACA>ATA	T>I	NADH	nonsyn
Mf|58	NC_006055:761490	G>A	EX	GGA>AGA	G>R	DNA	nonsyn
Mf|55	NC_006055:764884	C>A	EX	GAT>TAT	D>Y	hypothetical	nonsyn
Mf|50	NC_006055:769966	C>T	EX	GTT>ATT	V>I	phosphatidylglycerophosphate	nonsyn
Mf|58	NC_006055:770689	G>A	EX	TCA>TTA	S>L	putrescine/ornithine	nonsyn
Mf|62	NC_006055:771271	G>A	EX	ACT>ATT	T>I	putrescine/ornithine	nonsyn
Mf|65	NC_006055:771571	C>T	EX	GGT>GAT	G>D	putrescine/ornithine	nonsyn
Mf|50	NC_006055:772078	G>A	EX	CTT>TTT	L>F	purine-nucleoside	nonsyn
Mf|55	NC_006055:772909	C>T	EX	GGT>GAT	G>D	ribose	nonsyn
Mf|14	NC_006055:774218	G>A	EX	ACA>ATA	T>I	ribose	nonsyn
Mf|59	NC_006055:774359	C>T	EX	GGA>GAA	G>E	ribose	nonsyn
Mf|14	NC_006055:776162	G>A	EX	ACA>ATA	T>I	ribose	nonsyn
Mf|19	NC_006055:777359	G>C	EX	CCA>CGA	P>R	xylose	nonsyn
Mf|68	NC_006055:778817	G>A	EX	GCT>GTT	A>V	DNA-binding	nonsyn
Mf|68	NC_006055:778837	C>T	EX	GCG>GCA	A>A	DNA-binding	syn
Mf|68	NC_006055:778838	G>A	EX	GCG>GTG	A>V	DNA-binding	nonsyn
Mf|6	NC_006055:779199	C>A	EX	GCA>TCA	A>S	DNA-binding	nonsyn
Mf|56	NC_006055:779820	G>T	EX	ACT>AAT	T>N	tRNA	nonsyn
Mf|93	NC_006055:782021	C>A	EX	GCT>TCT	A>S	cell	nonsyn
Mf|50	NC_006055:782380	C>G	EX	GGT>GCT	G>A	cell	nonsyn
Mf|55	NC_006055:782440	G>A	EX	GCA>GTA	A>V	cell	nonsyn
Mf|12	NC_006055:785976	C>T	EX	GTT>ATT	V>I	DNA	nonsyn
Mf|76	NC_006055:787942	T>C	EX	GAA>GGA	E>G	DNA	nonsyn
Mf|55	NC_006055:790847	G>T	EX	CAA>AAA	Q>K	HAD-superfamily	nonsyn
Mf|58	NC_006055:791129	C>T	IG				
Mf|41	NC_006055:791249	G>T	EX	CGT>AGT	R>S	inner	nonsyn
Mf|62	NC_006055:791908	G>A	EX	ACA>ATA	T>I	inner	nonsyn

Se|70	NC_005003:1464	G>A	IG				
Se|66	NC_005008:1544	G>A	IG				
Se|62	NC_005004:1706	C>A	EX	CTT>ATT	L>I	hypothetical	nonsyn
Se|2	NC_005008:3500	T>C	EX	AGT>AGC	S>S	RepC	syn
Se|39	NC_005007:4079	C>A	EX	GCG>GAG	A>E	RLX	nonsyn
Se|39	NC_005003:5939	G>A	IG				
Se|75	NC_005004:10466	T>G	IG				
Se|38	NC_004461:13129	A>G	IG				
Se|75	NC_004461:14691	C>T	IG				
Se|67	NC_004461:17081	C>A	EX	CTG>ATG	L>M	homoserine-o-acetyltransferase	nonsyn
Se|11	NC_005004:17598	G>T	IG				
Se|3	NC_004461:21923	A>T	EX	TCA>TCT	S>S	replicative	syn
Se|38	NC_004461:34294	G>A	EX	GGG>GGA	G>G	teichoic	syn
Se|13	NC_004461:39377	A>T	IG				
Se|12	NC_004461:40860	A>T	EX	AAA>ATA	K>I	penicillin	nonsyn
Se|12	NC_004461:59880	A>G	EX	ATA>ACA	I>T	cassette	nonsyn
Se|66	NC_004461:60149	G>A	EX	TAC>TAT	Y>Y	cassette	syn
Se|70	NC_004461:69607	A>T	EX	GAT>GTT	D>V	hypothetical	nonsyn
Se|59	NC_004461:70211	G>A	EX	GTT>ATT	V>I	hypothetical	nonsyn
Se|63	NC_004461:82427	T>G	EX	GTC>GGC	V>G	regulatory	nonsyn
Se|59	NC_004461:86584	T>G	EX	ACT>ACG	T>T	dihydrolipoamide	syn
Se|12	NC_004461:108640	T>G	EX	GAA>GCA	E>A	pyridoxal-deC	nonsyn
Se|75	NC_004461:120131	C>T	IG				
Se|11	NC_004461:123802	G>T	EX	GAA>TAA	E>*	hypothetical	nonsyn
Se|2	NC_004461:132566	G>A	IG				
Se|62	NC_004461:170678	G>A	EX	GGA>AGA	G>R	adenosylmethionine-8-amino-7-oxononanc	nonsyn
Se|63	NC_004461:182083	G>T	EX	GCT>TCT	A>S	Zn-binding	nonsyn
Se|4	NC_004461:184153	C>T	IG				
Se|34	NC_004461:184980	A>T	EX	CAA>CAT	Q>H	NADH-dependent	nonsyn
Se|7	NC_004461:188114	G>A	EX	GGT>AGT	G>S	transmembrane	nonsyn
Se|2	NC_004461:201039	G>T	EX	AGT>ATT	S>I	ABC	nonsyn
Se|7	NC_004461:204655	C>A	EX	GCT>GAT	A>D	formate	nonsyn
Se|69	NC_004461:207022	C>A	EX	CGC>CTC	R>L	succinyl-diaminopimelate	nonsyn
Se|10	NC_004461:214647	A>G	EX	TTA>TTG	L>L	glycine	syn
Se|10	NC_004461:217646	A>C	EX	TTC>GTC	F>V	hypothetical	nonsyn
Se|70	NC_004461:224256	A>G	IG				
Se|2	NC_004461:228462	G>T	EX	GGC>GGA	G>G	multidrug	syn
Se|63	NC_004461:231309	T>G	EX	TTC>GTC	F>V	multidrug	nonsyn
Se|69	NC_004461:236135	C>T	EX	GCT>GTT	A>V	surfactin	nonsyn
Se|3	NC_004461:236986	T>A	EX	TAT>AAT	Y>N	surfactin	nonsyn
Se|12	NC_004461:255006	G>T	EX	GGG>TGG	G>W	branched-chain	nonsyn
Se|38	NC_004461:257948	C>T	EX	TCC>TCT	S>S	hypothetical	syn
Se|50	NC_004461:258275	T>C	IG				
Se|59	NC_004461:265982	C>T	EX	GGT>AGT	G>S	malate:quinone	nonsyn
Se|62	NC_004461:281215	T>A	IG				
Se|59	NC_004461:295465	C>T	EX	CAT>TAT	H>Y	tRNA/rRNA	nonsyn
Se|2	NC_004461:299847	G>T	EX	CGT>CTT	R>L	50S	nonsyn
Se|10	NC_004461:301563	A>G	IG				
Se|13	NC_004461:333412	C>T	EX	CAA>TAA	Q>*	Ser-Asp	nonsyn
Se|4	NC_004461:334590	C>T	EX	GAC>GAT	D>D	Ser-Asp	syn
Se|4	NC_004461:334629	C>A	EX	TCC>TCA	S>S	Ser-Asp	syn
Se|4	NC_004461:334632	T>C	EX	GAT>GAC	D>D	Ser-Asp	syn
Se|4	NC_004461:334641	G>A	EX	TCG>TCA	S>S	Ser-Asp	syn
Se|3	NC_004461:337670	C>A	EX	CAC>CAA	H>Q	hypothetical	nonsyn
Se|38	NC_004461:352812	C>T	EX	GGC>GGT	G>G	cationic	syn
Se|75	NC_004461:375658	A>T	EX	AAA>AAT	K>N	hypothetical	nonsyn
Se|75	NC_004461:380105	G>T	EX	GCG>GCT	A>A	iron-binding	syn
Se|3	NC_004461:388607	G>C	EX	TCT>TGT	S>C	hypothetical	nonsyn
Se|38	NC_004461:392574	G>C	EX	CGT>CCT	R>P	monovalent	nonsyn

Se|13	NC_004461:393788	C>T	EX	CAT>TAT	H>Y	monovalent	nonsyn
Se|75	NC_004461:433604	G>T	IG				
Se|13	NC_004461:447598	C>A	EX	GGT>GTT	G>V	undecaprenyl	nonsyn
Se|38	NC_004461:449335	C>A	EX	CCT>CAT	P>H	hypothetical	nonsyn
Se|65	NC_004461:449548	G>C	EX	GGT>GCT	G>A	hypothetical	nonsyn
Se|4	NC_004461:462739	A>T	EX	AAA>AAT	K>N	transcription	nonsyn
Se|34	NC_004461:489682	G>A	EX	GAT>AAT	D>N	ABC	nonsyn
Se|69	NC_004461:494579	A>T	IG				
Se|34	NC_004461:495944	C>A	EX	GCT>GAT	A>D	hypothetical	nonsyn
Se|10	NC_004461:497763	C>A	EX	GAC>TAC	D>Y	bifunctional	nonsyn
Se|75	NC_004461:501808	A>T	EX	TTA>TAA	L>*	di-tripepride	nonsyn
Se|70	NC_004461:502669	T>A	IG				
Se|7	NC_004461:514279	C>T	EX	GCA>ACA	A>T	hypothetical	nonsyn
Se|2	NC_004461:514708	C>T	IG				
Se|75	NC_004461:519140	C>T	EX	GGT>GAT	G>D	hypothetical	nonsyn
Se|67	NC_004461:523198	A>C	EX	GTC>GGC	V>G	hypothetical	nonsyn
Se|62	NC_004461:528592	T>G	EX	TTT>TTG	F>L	preprotein	nonsyn
Se|66	NC_004461:543285	C>T	EX	CTT>TTT	L>F	hypothetical	nonsyn
Se|8	NC_004461:545580	C>T	EX	GCA>GTA	A>V	hypothetical	nonsyn
Se|7	NC_004461:554343	A>G	EX	CAC>CGC	H>R	glyceraldehyde-3-phosphate	nonsyn
Se|12	NC_004461:572238	A>G	EX	TTA>CTA	L>L	hypothetical	syn
Se|39	NC_004461:579262	C>A	EX	GAA>TAA	E>*	hypothetical	nonsyn
Se|7	NC_004461:580374	T>A	IG				
Se|59	NC_004461:580448	G>C	IG				
Se|34	NC_004461:593568	T>A	EX	GGT>GGA	G>G	ABC	syn
Se|34	NC_004461:608615	C>G	IG				
Se|59	NC_004461:610535	C>G	EX	CTT>GTT	L>V	DltB	nonsyn
Se|70	NC_004461:627607	C>T	EX	GTG>ATG	V>M	monovalent	nonsyn
Se|66	NC_004461:645498	G>A	EX	GGT>AGT	G>S	hypothetical	nonsyn
Se|59	NC_004461:660859	G>A	EX	ATG>ATA	M>I	hypothetical	nonsyn
Se|4	NC_004461:668674	T>A	EX	GTT>GTA	V>V	3-oxoacyl-ACP	syn
Se|39	NC_004461:687790	T>A	EX	GAT>GAA	D>E	inorganic	nonsyn
Se|4	NC_004461:689530	G>A	EX	GAT>AAT	D>N	Mg2+	nonsyn
Se|13	NC_004461:700673	C>A	IG				
Se|62	NC_004461:706991	A>T	EX	CCT>CCA	P>P	diacylglycerol	syn
Se|59	NC_004461:710039	A>C	EX	AAA>ACA	K>T	peptide	nonsyn
Se|38	NC_004461:719747	A>G	IG				
Se|50	NC_004461:725555	T>G	IG				
Se|50	NC_004461:752293	T>G	EX	TGT>GGT	C>G	phosphoribosylaminoimidazole	nonsyn
Se|2	NC_004461:759880	G>T	EX	GAC>TAC	D>Y	phosphoribosylglycinamide	nonsyn
Se|13	NC_004461:763866	C>T	EX	GAA>AAA	E>K	cation	nonsyn
Se|11	NC_004461:775282	T>A	EX	ATT>ATA	I>I	cytochrome	syn
Se|70	NC_004461:796310	T>A	EX	GCA>GCT	A>A	manganese	syn
Se|7	NC_004461:817228	C>T	EX	ATG>ATA	M>I	hypothetical	nonsyn
Se|11	NC_004461:818705	C>T	EX	CAT>TAT	H>Y	hypothetical	nonsyn
Se|39	NC_004461:837123	G>A	EX	GCA>ACA	A>T	succinate	nonsyn
Se|50	NC_004461:842238	A>C	IG				
Se|69	NC_004461:855203	A>T	EX	AGT>TGT	S>C	cell	nonsyn
Se|3	NC_004461:891218	G>T	EX	GTC>TTC	V>F	PP2C	nonsyn
Se|67	NC_004461:894721	C>T	EX	ACA>ATA	T>I	ribulose-phosphate	nonsyn
Se|70	NC_004461:908615	G>T	EX	CCG>CCT	P>P	chromosome	syn
Se|12	NC_004461:912689	G>A	IG				
Se|39	NC_004461:931541	C>T	EX	GCT>GTT	A>V	ATP-dependent	nonsyn
Se|75	NC_004461:933004	A>T	EX	AAA>ATA	K>I	transcriptional	nonsyn
Se|67	NC_004461:934727	A>T	IG				
Se|75	NC_004461:941768	G>A	EX	GGC>AGC	G>S	prolyl-tRNA	nonsyn
Se|12	NC_004461:978277	A>G	EX	AAT>AGT	N>S	(dimethylallyl)adenosine	nonsyn
Se|39	NC_004461:1023530	G>T	IG				
Se|2	NC_004461:1073788	T>G	EX	CAA>CAC	Q>H	oligopeptide	nonsyn

Se|59	NC_004461:1073992	C>T	EX	TGG>TGA	W>*	oligopeptide	nonsyn
Se|59	NC_004461:1077752	G>A	EX	GGC>GAC	G>D	oligoendopeptidase	nonsyn
Se|50	NC_004461:1095693	T>G	EX	TAT>TCT	Y>S	ABC	nonsyn
Se|7	NC_004461:1111089	G>T	IG				
Se|10	NC_004461:1121744	G>T	IG				
Se|4	NC_004461:1123368	T>A	EX	GGA>GGT	G>G	undecaprenyldiphospho-muramoylpentape	syn
Se|3	NC_004461:1129122	C>T	EX	GAG>AAG	E>K	dihydrofolate	nonsyn
Se|10	NC_004461:1134366	C>A	EX	GCA>GAA	A>E	hypothetical	nonsyn
Se|75	NC_004461:1140329	C>A	EX	CTG>CTT	L>L	ebhA	syn
Se|8	NC_004461:1144884	G>A	EX	ACT>ATT	T>I	ebhA	nonsyn
Se|50	NC_004461:1158627	G>T	EX	ACA>AAA	T>K	ebhA	nonsyn
Se|62	NC_004461:1165805	G>A	EX	GCC>GCT	A>A	hypothetical	syn
Se|65	NC_004461:1167586	T>A	EX	CAA>CTA	Q>L	hypothetical	nonsyn
Se|11	NC_004461:1170320	G>T	EX	CAA>AAA	Q>K	hypothetical	nonsyn
Se|66	NC_004461:1182805	A>G	EX	TCA>CCA	S>P	dinG	nonsyn
Se|69	NC_004461:1193140	A>T	EX	CTT>CAT	L>H	3-dehydroquinate	nonsyn
Se|75	NC_004461:1195203	G>T	EX	AGC>AGA	S>R	nucleoside	nonsyn
Se|8	NC_004461:1215453	C>T	EX	GCT>ACT	A>T	hypothetical	nonsyn
Se|13	NC_004461:1223560	G>A	EX	AGT>AAT	S>N	glucose-6-phosphate	nonsyn
Se|62	NC_004461:1224078	G>A	EX	GAA>AAA	E>K	glucose-6-phosphate	nonsyn
Se|69	NC_004461:1235825	C>T	EX	ATG>ATA	M>I	branched-chain	nonsyn
Se|62	NC_004461:1243467	G>T	EX	GCG>GAG	A>E	hypothetical	nonsyn
Se|39	NC_004461:1250957	C>T	EX	AAG>AAA	K>K	Xaa-Pro	syn
Se|3	NC_004461:1254397	G>T	IG				
Se|59	NC_004461:1256500	C>T	EX	GAG>AAG	E>K	glycine	nonsyn
Se|3	NC_004461:1256590	C>A	EX	GCT>TCT	A>S	glycine	nonsyn
Se|10	NC_004461:1262915	A>T	EX	TTA>ATA	L>I	late	nonsyn
Se|62	NC_004461:1264324	A>T	EX	GTT>GAT	V>D	glucokinase	nonsyn
Se|50	NC_004461:1264683	G>T	EX	GCC>GCA	A>A	glucokinase	syn
Se|11	NC_004461:1267362	G>T	IG				
Se|75	NC_004461:1268447	G>A	EX	GCA>GTA	A>V	penicillin-binding	nonsyn
Se|7	NC_004461:1284691	T>C	EX	GAT>GGT	D>G	DNA	nonsyn
Se|10	NC_004461:1286588	C>T	EX	GCG>GCA	A>A	diacylglycerol	syn
Se|34	NC_004461:1291649	C>A	IG				
Se|75	NC_004461:1292049	G>A	IG				
Se|69	NC_004461:1302144	T>A	EX	AAA>ATA	K>I	GTP-binding	nonsyn
Se|70	NC_004461:1309714	T>A	EX	GAG>GTG	E>V	hypothetical	nonsyn
Se|2	NC_004461:1315145	C>A	EX	GAA>TAA	E>*	hypothetical	nonsyn
Se|11	NC_004461:1328303	C>T	EX	GTT>ATT	V>I	alanyl-tRNA	nonsyn
Se|10	NC_004461:1334078	T>C	EX	GAT>GGT	D>G	iron-sulfur	nonsyn
Se|50	NC_004461:1338625	G>A	IG				
Se|75	NC_004461:1342547	T>A	EX	ACA>TCA	T>S	histidyl-tRNA	nonsyn
Se|70	NC_004461:1353283	A>C	EX	TCT>TCG	S>S	bifunctional	syn
Se|59	NC_004461:1354941	G>T	EX	TTC>TTA	F>L	queuine	nonsyn
Se|75	NC_004461:1359215	C>G	EX	GAG>CAG	E>Q	GTPase	nonsyn
Se|75	NC_004461:1363245	C>G	EX	GAA>CAA	E>Q	hypothetical	nonsyn
Se|7	NC_004461:1369464	C>A	IG				
Se|67	NC_004461:1384791	G>T	EX	ACT>AAT	T>N	translation	nonsyn
Se|59	NC_004461:1388434	G>A	EX	GAC>GAT	D>D	threonyl-tRNA	syn
Se|62	NC_004461:1412544	C>T	EX	GAC>AAC	D>N	DNA	nonsyn
Se|7	NC_004461:1434516	A>T	EX	TTA>ATA	L>I	hypothetical	nonsyn
Se|75	NC_004461:1453525	C>A	EX	CAG>CAT	Q>H	DNA	nonsyn
Se|75	NC_004461:1475946	G>A	EX	GCA>GTA	A>V	FmtB	nonsyn
Se|7	NC_004461:1498044	G>T	EX	GAT>TAT	D>Y	hypothetical	nonsyn
Se|59	NC_004461:1499992	A>C	IG				
Se|38	NC_004461:1500452	G>T	EX	AGC>ATC	S>I	hypothetical	nonsyn
Se|13	NC_004461:1510172	G>A	EX	GAT>AAT	D>N	phosphoenolpyruvate	nonsyn
Se|11	NC_004461:1532097	G>T	EX	GCA>GAA	A>E	hypothetical	nonsyn
Se|59	NC_004461:1540678	T>C	EX	GAT>GGT	D>G	hypothetical	nonsyn

Se|69	NC_004461:1558656	C>A	IG				
Se|10	NC_004461:1558780	G>T	IG				
Se|2	NC_004461:1565389	G>T	IG				
Se|62	NC_004461:1592599	G>A	IG				
Se|67	NC_004461:1594035	C>G	IG				
Se|7	NC_004461:1609536	G>T	EX	GTA>TTA	V>L	hypothetical	nonsyn
Se|66	NC_004461:1612439	A>T	EX	CTC>CAC	L>H	teichoic	nonsyn
Se|12	NC_004461:1624187	C>A	EX	GCT>TCT	A>S	hypothetical	nonsyn
Se|2	NC_004461:1634967	C>A	EX	GGG>GGT	G>G	RNA	syn
Se|4	NC_004461:1636776	T>G	EX	AGA>AGC	R>S	aspartyl/glutamyl-tRNA	nonsyn
Se|13	NC_004461:1646298	G>A	EX	GCT>GTT	A>V	ATP-dependend	nonsyn
Se|3	NC_004461:1647093	C>T	EX	GTA>ATA	V>I	geranylgeranylglyceryl	nonsyn
Se|66	NC_004461:1656277	C>A	EX	GCT>TCT	A>S	hypothetical	nonsyn
Se|62	NC_004461:1661883	G>A	EX	CCG>CCA	P>P	hypothetical	syn
Se|62	NC_004461:1661886	T>C	EX	TTT>TTC	F>F	hypothetical	syn
Se|62	NC_004461:1661887	A>G	EX	ATT>GTT	I>V	hypothetical	nonsyn
Se|7	NC_004461:1663574	T>C	EX	GGA>GGG	G>G	bla	syn
Se|13	NC_004461:1686685	A>T	EX	TCA>ACA	S>T	Ser-Asp	nonsyn
Se|10	NC_004461:1693269	A>G	EX	TTA>CTA	L>L	fructokinase	syn
Se|50	NC_004461:1695354	A>T	EX	TTG>TAG	L>*	sucrose	nonsyn
Se|70	NC_004461:1718218	C>A	EX	CAG>AAG	Q>K	threonine	nonsyn
Se|70	NC_004461:1729555	G>T	EX	CAA>AAA	Q>K	sigmaB	nonsyn
Se|75	NC_004461:1737319	C>G	IG				
Se|3	NC_004461:1743238	A>G	EX	TAC>TGC	Y>C	cardiolipin	nonsyn
Se|75	NC_004461:1759723	A>T	EX	TTC>ATC	F>I	ATP	nonsyn
Se|66	NC_004461:1777105	G>A	IG				
Se|12	NC_004461:1781173	G>T	EX	CGA>AGA	R>R	spermine/spermidine	syn
Se|34	NC_004461:1784707	C>A	EX	GAG>GAT	E>D	amidase	nonsyn
Se|8	NC_004461:1785020	A>G	EX	GTA>GCA	V>A	amidase	nonsyn
Se|39	NC_004461:1796618	G>A	EX	GGC>GGT	G>G	hypothetical	syn
Se|10	NC_004461:1805460	A>T	EX	TTT>TTA	F>L	phosphoglucosamine	nonsyn
Se|75	NC_004461:1812882	C>G	IG				
Se|4	NC_004461:1818482	A>T	EX	TTT>TAT	F>Y	multidrug	nonsyn
Se|39	NC_004461:1833466	T>C	EX	AAA>GAA	K>E	alkaline	nonsyn
Se|12	NC_004461:1838760	G>T	IG				
Se|62	NC_004461:1848340	A>C	EX	TCA>TCC	S>S	NAD-dependent	syn
Se|59	NC_004461:1856258	A>T	EX	GAT>GAA	D>E	adenylate	nonsyn
Se|62	NC_004461:1856778	A>T	EX	ATG>AAG	M>K	adenylate	nonsyn
Se|8	NC_004461:1857670	C>T	EX	CAG>CAA	Q>Q	preprotein	syn
Se|10	NC_004461:1862923	C>T	EX	GGC>GAC	G>D	50S	nonsyn
Se|69	NC_004461:1865507	C>A	EX	GCG>GCT	A>A	50S	syn
Se|66	NC_004461:1865708	T>C	EX	GTA>GTG	V>V	50S	syn
Se|13	NC_004461:1884581	C>T	IG				
Se|4	NC_004461:1900254	A>T	EX	ACA>TCA	T>S	urease	nonsyn
Se|11	NC_004461:1900328	T>C	EX	CGT>CGC	R>R	urease	syn
Se|4	NC_004461:1904706	A>G	EX	ATT>GTT	I>V	urease	nonsyn
Se|34	NC_004461:1917551	A>T	EX	TTT>TTA	F>L	hypothetical	nonsyn
Se|38	NC_004461:1924227	A>T	EX	AAT>TAT	N>Y	hypothetical	nonsyn
Se|69	NC_004461:1926714	C>T	EX	GGC>GGT	G>G	PTS	syn
Se|38	NC_004461:1929542	G>T	EX	GCT>GAT	A>D	transport	nonsyn
Se|8	NC_004461:1930756	C>T	IG				
Se|59	NC_004461:1938335	G>T	EX	GGC>GGA	G>G	hypothetical	syn
Se|70	NC_004461:1974800	C>T	EX	CGT>TGT	R>C	two	nonsyn
Se|50	NC_004461:1984557	T>C	EX	AAT>GAT	N>D	teichoic	nonsyn
Se|8	NC_004461:1987990	A>T	IG				
Se|70	NC_004461:2000368	T>C	EX	AGC>GGC	S>G	hypothetical	nonsyn
Se|4	NC_004461:2011364	G>A	EX	CCA>TCA	P>S	respiratory	nonsyn
Se|7	NC_004461:2014683	C>T	EX	CGT>CAT	R>H	nitrite	nonsyn
Se|66	NC_004461:2014897	A>T	EX	TAT>AAT	Y>N	nitrite	nonsyn

Se|50	NC_004461:2029797	A>T	EX	TTA>ATA	L>I	ABC	nonsyn
Se|7	NC_004461:2029930	A>G	IG				
Se|39	NC_004461:2033666	T>A	EX	AAT>ATT	N>I	hypothetical	nonsyn
Se|75	NC_004461:2037051	T>G	EX	TTT>TGT	F>C	bicyclomycin	nonsyn
Se|10	NC_004461:2045532	G>A	EX	GGA>GAA	G>E	lipoprotein	nonsyn
Se|12	NC_004461:2057986	G>A	EX	GGT>AGT	G>S	sorbitol	nonsyn
Se|3	NC_004461:2060367	T>A	EX	TTT>TTA	F>L	amino	nonsyn
Se|8	NC_004461:2074414	C>A	EX	CTC>ATC	L>I	polyphosphate	nonsyn
Se|70	NC_004461:2094383	G>A	EX	CAA>TAA	Q>*	hypothetical	nonsyn
Se|59	NC_004461:2095515	T>C	EX	GTA>GCA	V>A	hypothetical	nonsyn
Se|12	NC_004461:2101576	G>A	IG				
Se|38	NC_004461:2119111	G>T	EX	CAC>AAC	H>N	NAD(P)H-flavin	nonsyn
Se|67	NC_004461:2138913	C>A	IG				
Se|69	NC_004461:2161041	C>T	IG				
Se|75	NC_004461:2164906	G>A	EX	GAG>AAG	E>K	copper-transporting	nonsyn
Se|13	NC_004461:2178128	A>T	EX	CTT>CAT	L>H	hypothetical	nonsyn
Se|7	NC_004461:2181155	T>A	EX	ATA>TTA	I>L	hypothetical	nonsyn
Se|7	NC_004461:2181488	A>T	IG				
Se|62	NC_004461:2182307	A>C	EX	GGT>GGG	G>G	TcaA	syn
Se|67	NC_004461:2184047	T>C	IG				
Se|63	NC_004461:2193342	T>G	IG				
Se|10	NC_004461:2197932	G>A	EX	GCT>GTT	A>V	hypothetical	nonsyn
Se|62	NC_004461:2203530	G>T	IG				
Se|4	NC_004461:2207856	G>A	EX	CAA>TAA	Q>*	pyruvate	nonsyn
Se|39	NC_004461:2224164	T>C	EX	ACA>ACG	T>T	anaerobic	syn
Se|70	NC_004461:2227104	G>T	EX	GCA>GAA	A>E	sulfate	nonsyn
Se|11	NC_004461:2271633	G>T	IG				
Se|59	NC_004461:2274246	A>T	IG				
Se|4	NC_004461:2274326	A>T	IG				
Se|50	NC_004461:2275555	C>A	EX	GGT>TGT	G>C	succinyl-diaminopimelate	nonsyn
Se|4	NC_004461:2276592	G>A	EX	TCG>TCA	S>S	hypothetical	syn
Se|75	NC_004461:2277035	G>A	EX	GGT>GAT	G>D	hypothetical	nonsyn
Se|75	NC_004461:2277046	G>T	EX	GTA>TTA	V>L	hypothetical	nonsyn
Se|2	NC_004461:2279764	G>A	EX	GGC>GGT	G>G	acetoin	syn
Se|34	NC_004461:2295975	G>A	EX	CGT>TGT	R>C	hypothetical	nonsyn
Se|70	NC_004461:2300541	G>A	EX	CAG>TAG	Q>*	hypothetical	nonsyn
Se|69	NC_004461:2302285	G>A	EX	CCT>TCT	P>S	lipopolysaccharide	nonsyn
Se|70	NC_004461:2307896	T>C	EX	GAC>GGC	D>G	hypothetical	nonsyn
Se|13	NC_004461:2309356	G>A	EX	GCG>GTG	A>V	preprotein	nonsyn
Se|69	NC_004461:2316667	C>T	IG				
Se|34	NC_004461:2321134	C>A	IG				
Se|38	NC_004461:2332105	A>C	EX	TTA>TTC	L>F	GntR	nonsyn
Se|62	NC_004461:2338126	G>A	IG				
Se|2	NC_004461:2340255	G>A	EX	CAT>TAT	H>Y	lysyl-tRNA	nonsyn
Se|66	NC_004461:2340742	C>A	IG				
Se|3	NC_004461:2342964	C>A	EX	GGC>GTC	G>V	cysteine	nonsyn
Se|70	NC_004461:2351050	C>T	EX	AGT>AAT	S>N	hypothetical	nonsyn
Se|10	NC_004461:2355344	G>T	EX	CAA>AAA	Q>K	transcription-repair	nonsyn
Se|50	NC_004461:2364631	C>A	EX	GAT>TAT	D>Y	veg	nonsyn
Se|11	NC_004461:2376115	T>C	EX	ATC>GTC	I>V	lysine	nonsyn
Se|39	NC_004461:2382242	A>T	IG				
Se|10	NC_004461:2415458	G>T	EX	GCA>GAA	A>E	hypothetical	nonsyn
Se|10	NC_004461:2427575	C>A	IG				
Se|62	NC_004461:2429329	G>T	EX	CCA>ACA	P>T	inositol-monophosphate	nonsyn
Se|66	NC_004461:2455399	C>T	EX	CGG>TGG	R>W	bifunctional	nonsyn
Se|62	NC_004461:2460674	G>A	EX	GCG>GTG	A>V	acetyl-CoA	nonsyn
Se|7	NC_004461:2472200	C>A	EX	TCG>TCT	S>S	Ser-Asp	syn
Se|50	NC_004461:2490267	G>A	EX	GGA>AGA	G>R	hypothetical	nonsyn
Vc|39	NZ_AAUT01000042:261	G>A	EX	CGC>TGC	R>C	fumarate	nonsyn

Vc|47	NZ_AAUT01000200:393	G>A	EX	AGC>AGT	S>S	outer	syn
Vc|9	NZ_AAUT01000041:445	G>A	EX	CCG>CCA	P>P	magnesium	syn
Vc|34	NZ_AAUT01000249:560	C>G	EX	GGG>GGC	G>G	UDP-glucose	syn
Vc|16	NZ_AAUT01000056:643	G>C	EX	GGC>GGG	G>G	aspartokinase,	syn
Vc|23	NZ_AAUT01000092:103	C>T	EX	CCT>TCT	P>S	GGDEF	nonsyn
Vc|31	NZ_AAUT01000175:104	C>A	EX	GGC>GGA	G>G	hypothetical	syn
Vc|47	NZ_AAUT01000006:149	G>A	EX	GTC>ATC	V>I	protein-P-II	nonsyn
Vc|68	NZ_AAUT01000029:166	A>G	EX	AAG>AGG	K>R	GTP	nonsyn
Vc|39	NZ_AAUT01000130:167	T>G	EX	ATC>CTC	I>L	Na+/H+	nonsyn
Vc|35	NZ_AAUT01000059:192	C>G	IG				
Vc|31	NZ_AAUT01000117:196	T>G	EX	GAT>GCT	D>A	glycogen	nonsyn
Vc|70	NZ_AAUT01000104:206	T>G	EX	TCC>GCC	S>A	GGDEF	nonsyn
Vc|39	NZ_AAUT01000101:208	C>A	EX	CCC>CCA	P>P	imidazolonepropionase	syn
Vc|32	NZ_AAUT01000050:210	A>G	EX	GTA>GCA	V>A	1-deoxyxylulose-5-phosphate	nonsyn
Vc|26	NZ_AAUT01000090:236	A>T	EX	ATC>AAC	I>N	sugar	nonsyn
Vc|33	NZ_AAUT01000108:244	G>A	EX	GCC>GTC	A>V	orotidine	nonsyn
Vc|19	NZ_AAUT01000087:254	C>A	IG				
Vc|1	NZ_AAUT01000007:274	C>T	EX	GCG>GTG	A>V	UDP-3-O-3-hydroxymyristoyl	nonsyn
Vc|59	NZ_AAUT01000032:281	C>T	EX	GGT>GAT	G>D	rod	nonsyn
Vc|70	NZ_AAUT01000032:315	G>A	EX	TCT>TTT	S>F	rod	nonsyn
Vc|16	NZ_AAUT01000019:323	C>T	EX	ATG>ATA	M>I	hypothetical	nonsyn
Vc|31	NZ_AAUT01000032:326	C>T	EX	GGT>AGT	G>S	rod	nonsyn
Vc|35	NZ_AAUT01000125:385	G>A	EX	GCA>GTA	A>V	adenylosuccinate	nonsyn
Vc|29	NZ_AAUT01000053:396	A>C	EX	GAT>GAG	D>E	sensor	nonsyn
Vc|8	NZ_AAUT01000085:429	C>T	EX	GAT>AAT	D>N	exodeoxyribonuclease	nonsyn
Vc|19	NZ_AAUT01000102:435	C>T	EX	CCT>CTT	P>L	isocitrate	nonsyn
Vc|16	NZ_AAUT01000122:439	T>A	EX	ATT>ATA	I>I	inner	syn
Vc|45	NZ_AAUT01000092:477	G>A	EX	CTG>CTA	L>L	hypothetical	syn
Vc|33	NZ_AAUT01000036:517	C>A	IG				
Vc|48	NZ_AAUT01000065:520	C>A	EX	ACC>ACA	T>T	UDP-glucose	syn
Vc|59	NZ_AAUT01000097:664	T>G	EX	GAT>GCT	D>A	hypothetical	nonsyn
Vc|20	NZ_AAUT01000075:765	G>A	EX	CCA>CTA	P>L	carbamoyl-phosphate	nonsyn
Vc|26	NZ_AAUT01000088:770	C>A	IG				
Vc|59	NZ_AAUT01000016:780	G>A	EX	GTC>ATC	V>I	hypothetical	nonsyn
Vc|45	NZ_AAUT01000048:786	G>A	EX	GGT>AGT	G>S	hypothetical	nonsyn
Vc|59	NZ_AAUT01000050:804	G>A	IG				
Vc|48	NZ_AAUT01000029:835	G>A	EX	CAA>TAA	Q>*	regulator	nonsyn
Vc|65	NZ_AAUT01000093:838	C>A	IG				
Vc|35	NZ_AAUT01000068:840	A>C	EX	GGT>GGG	G>G	spermidine/putrescine	syn
Vc|30	NZ_AAUT01000026:853	T>G	IG				
Vc|57	NZ_AAUT01000066:897	T>G	EX	ATC>CTC	I>L	exopolyphosphatase	nonsyn
Vc|34	NZ_AAUT01000075:954	G>T	IG				
Vc|43	NZ_AAUT01000041:977	T>C	EX	ATT>ACT	I>T	fructose-1,6-bisphosphatase	nonsyn
Vc|48	NZ_AAUT01000018:100	G>C	EX	GAC>GAG	D>E	multidrug	nonsyn
Vc|4	NZ_AAUT01000088:101	C>T	EX	CCA>TCA	P>S	aspartate	nonsyn
Vc|11	NZ_AAUT01000033:103	C>T	EX	CCC>CTC	P>L	single-stranded-DNA-specific	nonsyn
Vc|16	NZ_AAUT01000064:104	T>C	EX	CAT>CGT	H>R	hypothetical	nonsyn
Vc|31	NZ_AAUT01000059:106	G>T	EX	ACC>AAC	T>N	hypothetical	nonsyn
Vc|33	NZ_AAUT01000080:111	T>A	EX	TAT>AAT	Y>N	MutT/nudix	nonsyn
Vc|22	NZ_AAUT01000058:122	C>T	IG				
Vc|5	NZ_AAUT01000060:124	T>C	EX	TCC>CCC	S>P	hypothetical	nonsyn
Vc|31	NZ_AAUT01000010:130	T>G	EX	TTT>TTG	F>L	hypothetical	nonsyn
Vc|48	NZ_AAUT01000080:135	A>G	EX	AAC>AGC	N>S	4-hydroxyphenylpyruvate	nonsyn
Vc|17	NZ_AAUT01000035:140	A>G	EX	AGT>GGT	S>G	hypothetical	nonsyn
Vc|57	NZ_AAUT01000030:144	C>T	EX	GTT>ATT	V>I	hypothetical	nonsyn
Vc|22	NZ_AAUT01000056:146	G>A	IG				
Vc|35	NZ_AAUT01000004:147	G>A	EX	ACC>ATC	T>I	MdsD	nonsyn
Vc|34	NZ_AAUT01000007:151	C>T	EX	GGC>GGT	G>G	hypothetical	syn
Vc|4	NZ_AAUT01000047:155	G>A	EX	CCA>TCA	P>S	transcriptional	nonsyn

Vc|19	NZ_AAUT01000011:156	C>A	EX	CCC>ACC	P>T	hypothetical	nonsyn
Vc|11	NZ_AAUT01000066:158	C>T	EX	GCA>GTA	A>V	phosphate	nonsyn
Vc|59	NZ_AAUT01000068:160	T>C	EX	TGG>TGG	W>W	hypothetical	syn
Vc|1	NZ_AAUT01000064:161	C>T	EX	TGA>TAA	*>*	hypothetical	syn
Vc|41	NZ_AAUT01000013:164	G>T	EX	GCT>GAT	A>D	hypothetical	nonsyn
Vc|10	NZ_AAUT01000037:165	C>T	IG				
Vc|25	NZ_AAUT01000020:170	G>T	EX	GAG>GAT	E>D	sensor	nonsyn
Vc|1	NZ_AAUT01000021:171	C>T	IG				
Vc|30	NZ_AAUT01000054:179	C>T	EX	ATG>ATA	M>I	sulfate	nonsyn
Vc|19	NZ_AAUT01000046:181	T>G	EX	GGT>GGG	G>G	RTX	syn
Vc|11	NZ_AAUT01000016:184	T>G	IG				
Vc|30	NZ_AAUT01000010:185	C>T	IG				
Vc|49	NZ_AAUT01000029:195	G>T	EX	CTC>ATC	L>I	transporter	nonsyn
Vc|39	NZ_AAUT01000046:195	A>T	EX	CAA>CAT	Q>H	RTX	nonsyn
Vc|39	NZ_AAUT01000046:195	A>T	EX	CAA>CAT	Q>H	RTX	nonsyn
Vc|19	NZ_AAUT01000011:196	G>T	IG				
Vc|61	NZ_AAUT01000053:200	A>C	IG				
Vc|5	NZ_AAUT01000008:203	G>T	EX	GAG>TAG	E>*	hypothetical	nonsyn
Vc|23	NZ_AAUT01000064:210	C>A	EX	GAT>TAT	D>Y	3-oxoacyl-(acyl-carrier-protein)	nonsyn
Vc|16	NZ_AAUT01000043:211	C>T	EX	GAT>AAT	D>N	sensory	nonsyn
Vc|63	NZ_AAUT01000054:218	C>T	EX	GCC>ACC	A>T	GGDEF	nonsyn
Vc|43	NZ_AAUT01000004:219	C>T	EX	TTC>TTT	F>F	sodium/dicarboxylate	syn
Vc|53	NZ_AAUT01000035:223	T>G	EX	GTA>GTC	V>V	polysaccharide	syn
Vc|61	NZ_AAUT01000005:225	T>C	EX	TTT>TTC	F>F	hypothetical	syn
Vc|70	NZ_AAUT01000009:225	G>A	EX	CTG>CTA	L>L	lipase,	syn
Vc|39	NZ_AAUT01000037:225	G>A	IG				
Vc|17	NZ_AAUT01000018:232	C>T	EX	GAG>AAG	E>K	alpha-1,6-galactosidase,	nonsyn
Vc|4	NZ_AAUT01000006:237	C>A	EX	GAA>TAA	E>*	GGDEF	nonsyn
Vc|9	NZ_AAUT01000052:243	G>A	EX	GGC>AGC	G>S	aspartokinase,	nonsyn
Vc|49	NZ_AAUT01000033:271	T>C	EX	AGT>AGC	S>S	UDP-N-acetylmuramoylalanyl-D-glutamyl-2	syn
Vc|48	NZ_AAUT01000026:287	G>A	EX	GAG>GAA	E>E	glutaminyl-tRNA	syn
Vc|31	NZ_AAUT01000004:288	C>A	EX	CTC>ATC	L>I	methyl-accepting	nonsyn
Vc|63	NZ_AAUT01000026:295	A>G	EX	AAG>AGG	K>R	glutaminyl-tRNA	nonsyn
Vc|17	NZ_AAUT01000035:313	G>T	IG				
Vc|23	NZ_AAUT01000034:327	C>T	IG				
Vc|53	NZ_AAUT01000017:331	T>G	EX	GTG>GGG	V>G	flagellar	nonsyn
Vc|33	NZ_AAUT01000002:332	T>C	EX	ACC>GCC	T>A	oxaloacetate	nonsyn
Vc|35	NZ_AAUT01000027:333	A>G	EX	CTG>CCG	L>P	LuxO	nonsyn
Vc|33	NZ_AAUT01000030:346	C>T	IG				
Vc|8	NZ_AAUT01000013:347	G>T	IG				
Vc|43	NZ_AAUT01000014:370	T>A	IG				
Vc|37	NZ_AAUT01000009:388	G>A	EX	GCA>ACA	A>T	hydrolase,	nonsyn
Vc|47	NZ_AAUT01000021:393	G>T	EX	GCG>GCT	A>A	carbonic	syn
Vc|51	NZ_AAUT01000010:404	A>C	EX	TAT>TCT	Y>S	hypothetical	nonsyn
Vc|4	NZ_AAUT01000021:425	A>T	EX	TAC>AAC	Y>N	glycine	nonsyn
Vc|1	NZ_AAUT01000020:441	G>A	EX	GGC>AGC	G>S	cysteine	nonsyn
Vc|45	NZ_AAUT01000016:442	C>G	EX	AAC>AAG	N>K	vgrG	nonsyn
Vc|52	NZ_AAUT01000005:448	T>G	IG				
Vc|29	NZ_AAUT01000018:449	A>G	IG				
Vc|51	NZ_AAUT01000004:452	T>G	EX	CAA>CCA	Q>P	vgrG	nonsyn
Vc|28	NZ_AAUT01000004:467	G>A	EX	GGC>GGT	G>G	hcp	syn
Vc|53	NZ_AAUT01000008:471	G>A	EX	CTT>TTT	L>F	acyl	nonsyn
Vc|39	NZ_AAUT01000011:490	G>A	EX	CAA>TAA	Q>*	methyl-accepting	nonsyn
Vc|48	NZ_AAUT01000011:560	T>G	EX	CTA>CTC	L>L	6-phospho-beta-glucosidase	syn
Vc|22	NZ_AAUT01000012:584	C>T	EX	GTT>ATT	V>I	type	nonsyn
Vc|70	NZ_AAUT01000008:639	A>G	EX	CAG>CGG	Q>R	3-deoxy-D-manno-octulosonic-acid	nonsyn
Vc|57	NZ_AAUT01000002:644	A>G	EX	ATA>GTA	I>V	ToxR-activated	nonsyn
Vc|65	NZ_AAUT01000005:696	G>C	IG				
Vc|66	NZ_AAUT01000006:709	G>T	EX	CTG>CTT	L>L	transcriptional	syn

Vc|51	NZ_AAUT01000007:757!	C>T	IG				
Vc|34	NZ_AAUT01000007:761(	C>T	IG				
Vc|30	NZ_AAUT01000005:806:	T>G	EX	TTT>GTT	F>V	acetyltransferase,	nonsyn
Vc|23	NZ_AAUT01000002:857:	C>T	EX	TAC>TAT	Y>Y	leader	syn
Vc|37	NZ_AAUT01000002:903:	G>T	EX	GGT>TGT	G>C	tagE	nonsyn
Vc|63	NZ_AAUT01000001:954:	G>A	EX	ACA>ATA	T>I	sugar	nonsyn
Vc|51	NZ_AAUT01000001:971:	C>T	EX	CAA>TAA	Q>*	sodium/solute	nonsyn
Vc|31	NZ_AAUT01000003:975(	G>A	EX	AAG>AAA	K>K	hypothetical	syn
Vc|25	NZ_AAUT01000004:994!	C>T	EX	GTC>GTT	V>V	hypothetical	syn
Vc|45	NZ_AAUT01000002:103·	T>C	IG				
Vc|23	NZ_AAUT01000001:112!	C>T	EX	GAA>AAA	E>K	sucrose	nonsyn
Vc|10	NZ_AAUT01000003:120!	A>G	EX	GAT>GAC	D>D	quinolinate	syn
