# Supplementary material for: Evolution of the Insertion-Deletion Mutation Rate Across the Tree of Life
Source: G3 (Bethesda). 2016 Jun 15;6(8):2583–91. doi: 10.1534/g3.116.030890 (PMC4978911; doi:10.1534/g3.116.030890)
Supplement: Supplemental Material [file supp_g3.116.030890_DatasetS4.pdf]

Dataset S4.

List of insertion-deletion mutation calls for *Agrobacterium tumefaciens* (Agt), *Bacillus subtilis* (Bs), *Escherichia coli* (Ec), *Mesoplasma florum* (Mf), *Vibrio cholerae* (Vc), and *Caenorhabditis elegans* (Ce). IG - intergenic, EX - exon. Line column indicates the organism and MA line where indel is called seperated by (|). Algorithm (Alg.) is the mapping algorithm that identified the indel (N-Novoalign, B-Bwa, P-Pindel). Gene is the gene name or type of gene where the insertion/ deletion occurs, (-) indicates no known gene at location. Confirmed (conf.) column indicates if the mutation was verified using direct sequencing (seq), gel electrophoresis (gel), or did not amplify (-).

Line	Scaffold:Position	Size	Alg.			EX or IG	Gene	Conf.
			N	B	P			
Agt|44	NC_003064.2:4506	1	*	*	*	EX	fructosyl	
Agt|26	NC_003065.3:7362	1	*	*	*	EX	hypothetical	
Agt|36	NC_003065.3:71571	-1	*	*	-	IG	-	
Agt|42	NC_003062.2:82741	-4	*	*	*	EX	permease	
Agt|25	NC_003063.2:195916	1	*	*	*	IG	-	
Agt|30	NC_003063.2:225220	-1	*	*	-	EX	ftsk	
Agt|11	NC_003064.2:229665	1	*	*	*	EX	D-serine	
Agt|36	NC_003064.2:320813	-1	*	*	-	EX	D-isomer	
Agt|34	NC_003063.2:364471	1	*	*	-	IG	-	
Agt|55	NC_003064.2:392047	-1	*	*	-	EX	dihydrodipicolinate	
Agt|53	NC_003064.2:392047	-1	*	*	-	EX	dihydrodipicolinate	
Agt|5	NC_003063.2:504098	-3	*	*	-	EX	oligopeptide	
Agt|7	NC_003063.2:546849	6	*	*	*	EX	diguanylate	
Agt|2	NC_003063.2:625417	-1	*	*	-	EX	hypothetical	
Agt|30	NC_003062.2:762254	1	*	*	*	IG	-	
Agt|36	NC_003062.2:801590	1	*	*	-	EX	pyruvate,orthophosphate	
Agt|15	NC_003062.2:880411	-12	*	*	*	EX	acetyltransferase	
Agt|59	NC_003063.2:1029884	-1	*	*	*	EX	hypothetical	
Agt|44	NC_003062.2:1175889	-10307	-	-	*	IG	-	
Agt|9	NC_003062.2:1175889	-10307	-	-	*	IG	-	
Agt|39	NC_003063.2:1181859	1	*	*	*	EX	hypothetical	
Agt|25	NC_003062.2:1182402	-1	*	*	*	IG	-	
Agt|45	NC_003062.2:1182402	1	-	-	*	IG	-	
Agt|5	NC_003062.2:1191021	-1	*	*	*	EX	ABC	
Agt|26	NC_003062.2:1220203	-1	*	*	*	IG	-	
Agt|57	NC_003062.2:1220203	-1	*	*	-	IG	-	
Agt|29	NC_003062.2:1220203	1	*	*	-	IG	-	
Agt|46	NC_003062.2:1275276	-1	*	*	-	EX	hypothetical	
Agt|20	NC_003063.2:1306030	2	*	*	-	IG	-	
Agt|53	NC_003063.2:1306030	1	*	*	-	IG	-	
Agt|55	NC_003063.2:1306030	1	*	*	-	IG	-	
Agt|7	NC_003063.2:1380071	-1	*	*	*	EX	LacI	
Agt|51	NC_003063.2:1402387	-1	*	*	*	EX	LamB/YcsF	
Agt|4	NC_003063.2:1426511	-1	*	*	-	EX	hypothetical	
Agt|42	NC_003063.2:1447051	-1	*	*	*	IG	-	
Agt|58	NC_003062.2:1501701	-30	*	*	*	EX	single-strand	
Agt|44	NC_003063.2:1520361	1	*	*	*	EX	nitrite	
Agt|46	NC_003062.2:1538924	-1	*	*	-	EX	formate	
Agt|51	NC_003062.2:1538924	-1	*	*	*	EX	formate	
Agt|51	NC_003063.2:1556554	-1	*	*	*	IG	-	
Agt|32	NC_003063.2:1635958	1	*	*	*	IG	-	

Agt|55	NC_003063.2:1700261	-1	*	*	-	EX	myo-inositol	
Agt|28	NC_003062.2:1737920	-3	*	*	-	EX	hypothetical	
Agt|5	NC_003063.2:1789815	-1	*	*	-	EX	nolF	
Agt|59	NC_003063.2:1829949	-1	*	*	-	EX	ABC	
Agt|15	NC_003063.2:1829949	1	*	*	-	EX	ABC	
Agt|10	NC_003063.2:2040017	-1	*	*	*	IG	-	
Agt|40	NC_003062.2:2471250	1	*	*	-	IG	-	
Agt|26	NC_003062.2:2829423	-1	*	*	*	EX	hypothetical	
Agt|7	NC_003062.2:2838293	-1	*	*	*	IG	-	
Bs|7	NZ_CM000488.1:3154	1	*	*	*	IG	-	seq
Bs|57	NZ_CM000488.1:29645	-1	*	*	*	EX	hypothetical	
Bs|46	plasmid:31466	-6	*	*	*	IG	-	
Bs|20	plasmid:59759	-66	-	-	*	IG	-	
Bs|19	NZ_CM000488.1:90374	-1	*	*	*	IG	-	seq
Bs|30	NZ_CM000488.1:90374	-1	*	*	*	IG	-	
Bs|45	NZ_CM000488.1:267583	1	*	*	*	EX	hypothetical	
Bs|35	NZ_CM000488.1:367560	-1	*	*	*	EX	hypothetical	
Bs|26	NZ_CM000488.1:446576	-10	-	-	*	EX	hypothetical	seq
Bs|3	NZ_CM000488.1:491130	-30	*	*	*	EX	manganese	
Bs|69	NZ_CM000488.1:491130	-30	*	*	*	EX	manganese	
Bs|22	NZ_CM000488.1:491130	-30	*	*	*	EX	manganese	
Bs|68	NZ_CM000488.1:491130	-30	*	*	*	EX	manganese	
Bs|28	NZ_CM000488.1:491130	-30	*	*	*	EX	manganese	
Bs|32	NZ_CM000488.1:491130	-30	*	*	*	EX	manganese	
Bs|35	NZ_CM000488.1:491130	-30	*	*	*	EX	manganese	
Bs|30	NZ_CM000488.1:491130	-30	-	-	*	EX	manganese	
Bs|27	NZ_CM000488.1:491130	-30	-	-	*	EX	manganese	
Bs|36	NZ_CM000488.1:491130	-30	-	-	*	EX	manganese	
Bs|71	NZ_CM000488.1:491130	-30	-	-	*	EX	manganese	
Bs|74	NZ_CM000488.1:491130	-30	-	-	*	EX	manganese	
Bs|38	NZ_CM000488.1:491130	-30	-	-	*	EX	manganese	
Bs|40	NZ_CM000488.1:491130	-30	-	-	*	EX	manganese	
Bs|43	NZ_CM000488.1:491130	-30	-	-	*	EX	manganese	
Bs|65	NZ_CM000488.1:491130	-30	-	-	*	EX	manganese	
Bs|39	NZ_CM000488.1:491130	-30	-	-	*	EX	manganese	
Bs|31	NZ_CM000488.1:491130	-30	-	-	*	EX	manganese	
Bs|37	NZ_CM000488.1:491130	-30	-	-	*	EX	manganese	
Bs|20	NZ_CM000488.1:505821	-1	*	*	*	EX	hypothetical	
Bs|62	NZ_CM000488.1:524912	-1	*	*	*	EX	hypothetical	seq
Bs|6	NZ_CM000488.1:525198	1	*	*	*	IG	-	seq
Bs|62	NZ_CM000488.1:525198	1	*	*	*	IG	-	
Bs|27	NZ_CM000488.1:531210	-1	*	*	*	IG	-	
Bs|6	NZ_CM000488.1:555880	1	*	*	*	IG	-	seq
Bs|1	NZ_CM000488.1:559201	1	*	*	*	IG	-	seq
Bs|26	NZ_CM000488.1:599768	1	*	*	*	EX	hypothetical	seq
Bs|30	NZ_CM000488.1:607751	-1	*	*	*	IG	-	
Bs|68	NZ_CM000488.1:615440	-1	*	*	*	EX	hypothetical	
Bs|14	NZ_CM000488.1:626136	-1	*	*	*	EX	hypothetical	
Bs|45	NZ_CM000488.1:755445	1	*	*	*	EX	polypeptide	
Bs|62	NZ_CM000488.1:800459	11	*	*	*	IG	-	--
Bs|32	NZ_CM000488.1:886044	-12	*	*	*	EX	hypothetical	--
Bs|54	NZ_CM000488.1:923830	1	*	*	*	EX	YfhE	
Bs|28	NZ_CM000488.1:959034	-9	*	*	*	EX	vegetative	--
Bs|8	NZ_CM000488.1:960654	1	*	*	-	EX	aliphatic	
Bs|3	NZ_CM000488.1:1073004	6	*	*	*	IG	-	--
Bs|31	NZ_CM000488.1:1073004	6	*	*	*	IG	-	--
Bs|45	NZ_CM000488.1:1100167	-1	*	*	*	IG	-	
Bs|22	NZ_CM000488.1:1151601	-1	*	*	*	EX	hypothetical	seq
Bs|37	NZ_CM000488.1:1209732	-1	*	*	*	IG	-	

Bs|1	NZ_CM000488.1:1327050	-1	*	*	*	EX	hypothetical	
Bs|69	NZ_CM000488.1:1335634	24	-	-	*	EX	hypothetical	seq
Bs|11	NZ_CM000488.1:1425882	1	*	*	*	EX	transaminase	seq
Bs|15	NZ_CM000488.1:1466869	1	*	*	*	IG	-	
Bs|55	NZ_CM000488.1:1547731	1	*	*	*	EX	hypothetical	
Bs|51	NZ_CM000488.1:1630257	-5	*	*	-	EX	phosphoadenosine	--
Bs|24	NZ_CM000488.1:1676959	-1	*	*	*	EX	ribonuclease	seq
Bs|26	NZ_CM000488.1:1676959	-1	*	*	*	EX	ribonuclease	
Bs|65	NZ_CM000488.1:1714855	-1	*	*	*	EX	inhibition	seq
Bs|28	NZ_CM000488.1:1740917	-1	*	*	*	EX	hypothetical	
Bs|38	NZ_CM000488.1:1755104	-1	*	*	*	IG	-	
Bs|45	NZ_CM000488.1:1777288	-309	-	-	*	EX	DNA	gel
Bs|4	NZ_CM000488.1:1866729	1	*	*	*	EX	RNA-binding	seq
Bs|36	NZ_CM000488.1:1922151	-1	*	*	*	EX	hypothetical	
Bs|45	NZ_CM000488.1:1938149	-1401	-	-	*	EX	amino	
Bs|48	NZ_CM000488.1:1938149	-1401	-	-	*	EX	amino	
Bs|51	NZ_CM000488.1:1938149	-1401	-	-	*	EX	amino	
Bs|50	NZ_CM000488.1:1938149	-1401	-	-	*	EX	amino	
Bs|44	NZ_CM000488.1:1938149	-1401	-	-	*	EX	amino	
Bs|41	NZ_CM000488.1:1938149	-1401	-	-	*	EX	amino	
Bs|52	NZ_CM000488.1:1938149	-1401	-	-	*	EX	amino	
Bs|46	NZ_CM000488.1:1938149	-1401	-	-	*	EX	amino	
Bs|49	NZ_CM000488.1:1938149	-1401	-	-	*	EX	amino	
Bs|69	NZ_CM000488.1:1943490	4	*	*	*	EX	endo-1,4-beta-xylanase	
Bs|8	NZ_CM000488.1:1944358	-4136	-	-	*	EX	endo-1,4-beta-xylanase	gel
Bs|45	NZ_CM000488.1:2064605	-1	*	*	*	EX	hypothetical	
Bs|26	NZ_CM000488.1:2068187	-1	*	*	*	EX	hypothetical	
Bs|22	NZ_CM000488.1:2097878	-1	*	*	*	EX	hypothetical	
Bs|35	NZ_CM000488.1:2152742	1	*	*	*	EX	hypothetical	seq
Bs|74	NZ_CM000488.1:2154392	1	*	*	*	EX	hypothetical	
Bs|41	NZ_CM000488.1:2170495	1	*	*	*	EX	hypothetical	seq
Bs|32	NZ_CM000488.1:2228381	-1	*	*	*	IG	-	
Bs|63	NZ_CM000488.1:2256708	1	*	*	*	EX	hypothetical	
Bs|60	NZ_CM000488.1:2289257	-1	*	*	*	EX	hypothetical	seq
Bs|31	NZ_CM000488.1:2304761	-2	*	*	*	EX	homoserine	
Bs|27	NZ_CM000488.1:2316414	-1	*	*	*	EX	naringenin-chalcone	seq
Bs|69	NZ_CM000488.1:2317269	-1	*	*	*	IG	-	
Bs|24	NZ_CM000488.1:2319727	-1	*	*	*	EX	hypothetical	
Bs|36	NZ_CM000488.1:2383704	-1	*	*	*	EX	transcription	
Bs|46	NZ_CM000488.1:2419719	-1	*	*	*	EX	required	
Bs|40	NZ_CM000488.1:2426419	-1	*	*	*	EX	hypothetical	
Bs|13	NZ_CM000488.1:2441288	-2	*	*	*	EX	sporulation	seq
Bs|35	NZ_CM000488.1:2485995	-1	*	*	*	EX	hypothetical	seq
Bs|38	NZ_CM000488.1:2493380	1	*	*	*	EX	multidrug	
Bs|19	NZ_CM000488.1:2513723	-1	*	*	*	EX	hypothetical	
Bs|51	NZ_CM000488.1:2543900	1	*	*	*	EX	hypothetical	
Bs|24	NZ_CM000488.1:2673381	19	-	-	*	EX	hypothetical	seq
Bs|63	NZ_CM000488.1:2679304	1	*	*	*	IG	-	
Bs|62	NZ_CM000488.1:2716393	-1	*	*	*	EX	multidrug-efflux	
Bs|49	NZ_CM000488.1:2731200	1	*	*	*	EX	cytochrome	
Bs|2	NZ_CM000488.1:2813831	1	*	*	*	EX	hypothetical	seq
Bs|54	NZ_CM000488.1:2856918	-2	-	-	*	IG	-	
Bs|17	NZ_CM000488.1:2863466	-1	*	*	*	EX	spatial	
Bs|63	NZ_CM000488.1:2868738	-2	*	*	-	IG	-	seq
Bs|39	NZ_CM000488.1:2924109	-1	*	*	*	IG	-	
Bs|43	NZ_CM000488.1:2924109	-1	*	*	*	IG	-	
Bs|20	NZ_CM000488.1:2938004	-2	*	*	*	EX	alpha-L-arabinofuranosidase	
Bs|55	NZ_CM000488.1:2950906	1	*	*	*	EX	hypothetical	
Bs|36	NZ_CM000488.1:2993837	1	*	*	*	EX	hypothetical	

Bs|45	NZ_CM000488.1:3009637	-1	*	*	*	IG	-	seq
Bs|6	NZ_CM000488.1:3009637	-1	*	*	*	IG	-	
Bs|55	NZ_CM000488.1:3051738	-1	*	*	*	IG	-	
Bs|43	NZ_CM000488.1:3058502	-1	*	*	*	IG	-	seq
Bs|45	NZ_CM000488.1:3058502	-1	*	*	*	IG	-	
Bs|54	NZ_CM000488.1:3104151	-1	*	*	*	IG	-	
Bs|60	NZ_CM000488.1:3371555	-1	*	*	*	IG	-	
Bs|39	NZ_CM000488.1:3371577	-1	*	*	*	EX	hypothetical	
Bs|17	NZ_CM000488.1:3543935	1	*	*	*	EX	hypothetical	seq
Bs|55	NZ_CM000488.1:3651513	-1	*	*	*	EX	biosynthesis	
Bs|22	NZ_CM000488.1:3696920	-1	*	*	*	EX	hypothetical	
Bs|39	NZ_CM000488.1:3706735	-1	*	*	*	IG	-	
Bs|6	NZ_CM000488.1:3769959	-1	*	*	*	EX	hypothetical	seq
Bs|74	NZ_CM000488.1:3769959	-1	*	*	*	EX	hypothetical	
Bs|48	NZ_CM000488.1:3815217	-21	*	*	*	EX	hypothetical	--
Bs|6	NZ_CM000488.1:3873136	-1	*	*	*	EX	hypothetical	
Bs|69	NZ_CM000488.1:3879665	-44	-	-	*	IG	-	seq
Bs|8	NZ_CM000488.1:3933228	-39	-	-	*	EX	hypothetical	
Bs|8	NZ_CM000488.1:3933266	-1	*	*	*	EX	hypothetical	seq
Bs|17	NZ_CM000488.1:4100765	-1	*	*	*	EX	hypothetical	
Ce|529	NC_003284:21431	1	*	*	NA	IG	-	
Ce|523	NC_003279:229092	-1	*	*	NA	IG	-	
Ce|529	NC_003279:229092	-1	*	*	NA	IG	-	
Ce|538	NC_003279:230551	-1	*	*	NA	IG	-	
Ce|553	NC_003279:230554	-1	*	*	NA	IG	-	
Ce|553	NC_003281:313084	1	*	*	NA	EX	Y50D7A.1	
Ce|574	NC_003280:362422	-1	*	*	NA	IG	-	
Ce|529	NC_003284:425461	1	*	*	NA	EX	ZK1193.4	
Ce|574	NC_003282:440589	-1	*	*	NA	IG	-	
Ce|523	NC_003284:538145	-1	*	*	NA	EX	F28C10.4	
Ce|574	NC_003281:666843	1	*	*	NA	IG	-	
Ce|574	NC_003279:774903	1	*	*	NA	EX	T06A4.1	
Ce|538	NC_003283:941768	-1	*	*	NA	EX	Y50D4C.3	
Ce|574	NC_003282:1480525	1	*	*	NA	IG	-	
Ce|574	NC_003280:1580398	2	*	*	NA	IG	-	
Ce|553	NC_003284:2428247	-1	*	*	NA	IG	-	
Ce|574	NC_003283:2606202	1	*	*	NA	EX	F53E10.3	
Ce|574	NC_003280:2642035	1	*	*	NA	IG	-	
Ce|574	NC_003279:3259385	-1	*	*	NA	IG	-	
Ce|574	NC_003284:3271341	1	*	*	NA	IG	-	
Ce|574	NC_003284:3314891	1	*	*	NA	IG	-	
Ce|574	NC_003284:3413738	1	*	*	NA	EX	dhs-27	
Ce|523	NC_003284:3860506	1	*	*	NA	IG	-	
Ce|538	NC_003284:3867534	2	*	*	NA	IG	-	
Ce|523	NC_003284:3875662	-1	*	*	NA	IG	-	
Ce|574	NC_003281:4007037	1	*	*	NA	EX	pxd-1	
Ce|526	NC_003281:4196878	1	*	*	NA	IG	-	
Ce|574	NC_003284:4216410	1	*	*	NA	EX	-	
Ce|529	NC_003281:5053760	1	*	*	NA	IG	-	
Ce|523	NC_003279:5357110	-1	*	*	NA	IG	-	
Ce|574	NC_003284:6013738	1	*	*	NA	IG	-	
Ce|529	NC_003280:6533103	-1	*	*	NA	EX	abcx-1	
Ce|574	NC_003280:6533103	-1	*	*	NA	EX	abcx-1	
Ce|538	NC_003284:6606591	1	*	*	NA	IG	-	
Ce|538	NC_003280:6657828	1	*	*	NA	EX	T19D12.10	
Ce|523	NC_003280:6730826	-1	*	*	NA	EX	T14B4.9	
Ce|523	NC_003280:6730862	-1	*	*	NA	EX	T14B4.9	
Ce|538	NC_003280:6730862	-1	*	*	NA	EX	T14B4.9	
Ce|523	NC_003283:6896914	1	*	*	NA	IG	-	

Ce|538	NC_003283:6972737	1	*	*	NA	EX	srx-59
Ce|538	NC_003280:6999650	1	*	*	NA	EX	unc-104
Ce|529	NC_003284:7068593	1	*	*	NA	IG	-
Ce|574	NC_003284:7068593	1	*	*	NA	IG	-
Ce|529	NC_003280:7422380	1	*	*	NA	IG	-
Ce|574	NC_003280:7422380	1	*	*	NA	IG	-
Ce|553	NC_003281:7453357	-2	*	*	NA	IG	-
Ce|574	NC_003281:7643635	1	*	*	NA	IG	-
Ce|553	NC_003279:7879125	1	*	*	NA	IG	-
Ce|574	NC_003284:8869901	1	*	*	NA	EX	ubc-21
Ce|523	NC_003284:8900564	1	*	*	NA	IG	-
Ce|529	NC_003284:8900564	1	*	*	NA	IG	-
Ce|529	NC_003281:8910563	1	*	*	NA	EX	unc-32
Ce|523	NC_003280:8916809	-1	*	*	NA	IG	-
Ce|538	NC_003284:9748181	-1	*	*	NA	EX	him-4
Ce|574	NC_003284:9999442	1	*	*	NA	IG	-
Ce|523	NC_003284:10298182	-1	*	*	NA	IG	-
Ce|574	NC_003280:10830982	1	*	*	NA	EX	mix-1
Ce|574	NC_003284:10841781	-1	*	*	NA	IG	-
Ce|529	NC_003283:10866552	-1	*	*	NA	IG	-
Ce|526	NC_003281:10896013	1	*	*	NA	IG	-
Ce|574	NC_003280:11318332	-1	*	*	NA	IG	-
Ce|529	NC_003280:11769647	1	*	*	NA	IG	-
Ce|574	NC_003284:11773775	-1	*	*	NA	IG	-
Ce|538	NC_003281:12059763	1	*	*	NA	IG	-
Ce|574	NC_003282:12335742	-1	*	*	NA	EX	-
Ce|538	NC_003284:12435505	1	*	*	NA	EX	T18D3.7
Ce|574	NC_003284:12435505	1	*	*	NA	EX	T18D3.7
Ce|526	NC_003281:12672223	1	*	*	NA	EX	taf-7.2
Ce|523	NC_003282:12883827	1	*	*	NA	IG	-
Ce|538	NC_003282:13450032	1	*	*	NA	EX	-
Ce|538	NC_003279:13644260	-1	*	*	NA	EX	dkf-1
Ce|523	NC_003282:13764410	1	*	*	NA	EX	sorb-1
Ce|538	NC_003282:13793521	-1	*	*	NA	EX	Y45F10D.7
Ce|538	NC_003284:14075543	-2	*	*	NA	EX	C33G3.4
Ce|574	NC_003279:14169356	1	*	*	NA	IG	-
Ce|538	NC_003284:14523332	-1	*	*	NA	IG	-
Ce|574	NC_003283:14524355	1	*	*	NA	IG	-
Ce|529	NC_003280:15051037	-1	*	*	NA	IG	-
Ce|529	NC_003283:15835625	1	*	*	NA	IG	-
Ce|538	NC_003283:18170182	1	*	*	NA	EX	C14A6.8
Ec|65	NC_000913.2:19777	-18	-	-	*	IG	-
Ec|29	NC_000913.2:59170	-1	*	*	*	IG	-
Ec|63	NC_000913.2:117578	-14	*	*	*	IG	-
Ec|93	NC_000913.2:220480	-3	*	*	*	EX	DL-methionine
Ec|45	NC_000913.2:293748	-1	*	*	*	EX	CP4-6
Ec|28	NC_000913.2:353860	-94	-	-	*	IG	-
Ec|35	NC_000913.2:566008	-1250	-	-	*	IG	-
Ec|65	NC_000913.2:573676	-137	-	-	*	IG	-
Ec|74	NC_000913.2:681083	-1	*	*	*	EX	Hsp70
Ec|17	NC_000913.2:1003376	-1	*	*	*	EX	putative
Ec|38	NC_000913.2:1197676	8	-	-	*	IG	-
Ec|12	NC_000913.2:1211303	1	*	*	*	IG	-
Ec|39	NC_000913.2:1211303	1	*	*	*	IG	-
Ec|28	NC_000913.2:1211303	-1	*	*	*	IG	-
Ec|71	NC_000913.2:1211303	1	*	*	*	IG	-
Ec|60	NC_000913.2:1211303	1	*	*	*	IG	-
Ec|65	NC_000913.2:1211303	1	*	*	*	IG	-
Ec|48	NC_000913.2:1211303	1	*	*	*	IG	-

Ec|63	NC_000913.2:1211303	1	*	*	*	IG	-
Ec|14	NC_000913.2:1419655	-1	*	*	*	EX	Rac
Ec|45	NC_000913.2:1419655	-1	*	*	*	EX	Rac
Ec|6	NC_000913.2:1491025	-18	*	*	*	EX	methyl-accepting
Ec|39	NC_000913.2:1592148	-1	*	*	*	IG	-
Ec|46	NC_000913.2:1811456	1	*	*	*	EX	cell
Ec|38	NC_000913.2:1869850	-1	*	*	*	EX	putative
Ec|86	NC_000913.2:1918710	1	*	*	*	EX	16S
Ec|91	NC_000913.2:1970624	1	*	*	*	EX	methyl-accepting
Ec|89	NC_000913.2:1970624	1	*	*	*	EX	methyl-accepting
Ec|44	NC_000913.2:2162577	-4	*	*	*	EX	DNA-binding
Ec|50	NC_000913.2:2255606	-6	*	*	*	EX	pseudouridine
Ec|6	NC_000913.2:2404562	9	*	*	*	EX	DNA-binding
Ec|9	NC_000913.2:2558975	-24	-	-	*	IG	-
Ec|53	NC_000913.2:2558975	-24	-	-	*	IG	-
Ec|9	NC_000913.2:2558997	-1	*	*	-	IG	-
Ec|53	NC_000913.2:2558997	-1	*	*	-	IG	-
Ec|37	NC_000913.2:2763433	-8	*	*	*	IG	-
Ec|81	NC_000913.2:2763433	8	-	-	*	IG	-
Ec|14	NC_000913.2:2763433	8	-	-	*	IG	-
Ec|93	NC_000913.2:2763433	-24	-	-	*	IG	-
Ec|62	NC_000913.2:2792402	-1	*	*	*	EX	gamma-aminobutyrate
Ec|50	NC_000913.2:2970933	-69	-	-	*	EX	lysophospholipid
Ec|80	NC_000913.2:2993775	-1	*	*	*	IG	-
Ec|96	NC_000913.2:3133909	-1	*	*	*	EX	phosphate
Ec|44	NC_000913.2:3188268	-1	*	*	*	EX	putative
Ec|93	NC_000913.2:3244075	-12	*	*	*	EX	hexuronate
Ec|14	NC_000913.2:3795056	-1	*	*	*	EX	O-antigen
Ec|100	NC_000913.2:3939973	1	-	-	*	IG	-
Ec|100	NC_000913.2:4034218	1	-	-	*	IG	-
Ec|99	NC_000913.2:4106780	1	*	*	*	IG	-
Ec|12	NC_000913.2:4282422	-1	*	*	*	EX	acetate
Ec|59	NC_000913.2:4311169	1	*	*	*	IG	-
Ec|45	NC_000913.2:4432572	39	-	-	*	IG	-
Ec|45	NC_000913.2:4604345	-1	*	*	*	IG	-
Mf|26	NC_006055.1:1349	-1	*	*	*	IG	-
Mf|59	NC_006055.1:32099	1	*	*	*	IG	-
Mf|85	NC_006055.1:44016	1	*	*	*	EX	transcriptional
Mf|11	NC_006055.1:44576	-2	*	*	*	IG	-
Mf|92	NC_006055.1:44576	-1	*	*	-	IG	-
Mf|59	NC_006055.1:44576	-1	*	*	-	IG	-
Mf|62	NC_006055.1:44576	-1	*	*	-	IG	-
Mf|57	NC_006055.1:44576	-1	*	*	-	IG	-
Mf|6	NC_006055.1:51562	-1	*	*	*	EX	NADH
Mf|62	NC_006055.1:64141	-1	*	*	*	EX	Mg2+
Mf|83	NC_006055.1:64198	-1	*	*	*	EX	Mg2+
Mf|41	NC_006055.1:85079	-1	*	*	*	EX	prolipoprotein
Mf|47	NC_006055.1:124252	1	*	*	*	EX	lysophospholipase
Mf|92	NC_006055.1:125589	2	*	*	*	EX	DNA
Mf|55	NC_006055.1:180163	1	*	*	*	EX	substrate
Mf|68	NC_006055.1:187300	1	*	*	*	EX	K+,
Mf|11	NC_006055.1:198193	1	*	*	*	EX	substrate
Mf|49	NC_006055.1:208454	-11	*	*	*	IG	-
Mf|31	NC_006055.1:210238	1	*	*	*	EX	fructose-specific
Mf|39	NC_006055.1:210238	1	*	*	*	EX	fructose-specific
Mf|68	NC_006055.1:210302	-1	*	*	*	EX	fructose-specific
Mf|6	NC_006055.1:212185	5	*	*	*	EX	fructose-specific
Mf|42	NC_006055.1:229215	1	*	*	*	EX	DNA
Mf|92	NC_006055.1:246712	1	*	*	*	EX	serine/threonine

Mf|23	NC_006055.1:247723	1	*	*	*	EX	serine/threonine
Mf|67	NC_006055.1:251013	1	*	*	*	IG	-
Mf|65	NC_006055.1:251037	1	*	*	*	IG	-
Mf|92	NC_006055.1:259234	-1	*	*	*	IG	-
Mf|67	NC_006055.1:263856	-1	*	*	*	EX	phosphate
Mf|59	NC_006055.1:271602	6	*	*	*	EX	uracil-DNA
Mf|39	NC_006055.1:284202	-2	*	*	*	IG	-
Mf|56	NC_006055.1:284202	-4	*	*	*	IG	-
Mf|26	NC_006055.1:285396	1	*	*	*	EX	hypothetical
Mf|49	NC_006055.1:292616	-6	*	*	*	EX	substrate
Mf|56	NC_006055.1:292616	-6	*	*	*	EX	substrate
Mf|67	NC_006055.1:314152	-1	*	*	*	EX	hypothetical
Mf|67	NC_006055.1:344335	1	*	*	*	EX	ATP/GTP
Mf|31	NC_006055.1:350681	1	*	*	*	EX	hypothetical
Mf|57	NC_006055.1:351102	-1	*	*	*	EX	hypothetical
Mf|50	NC_006055.1:351379	1	*	*	*	EX	hypothetical
Mf|90	NC_006055.1:352376	-1	*	*	*	EX	hypothetical
Mf|83	NC_006055.1:352427	1	*	*	*	EX	hypothetical
Mf|83	NC_006055.1:354321	-1	*	*	*	EX	type
Mf|49	NC_006055.1:354321	-1	*	*	*	EX	type
Mf|58	NC_006055.1:362283	-1	*	*	*	EX	beta-glucoside
Mf|58	NC_006055.1:373939	-1672	-	-	*	EX	beta-glucoside
Mf|57	NC_006055.1:377897	5	*	*	*	EX	exodeoxyribonuclease
Mf|19	NC_006055.1:386317	-1	*	*	*	EX	DNA
Mf|99	NC_006055.1:386611	-1	*	*	*	EX	DNA
Mf|42	NC_006055.1:391758	1	*	*	*	EX	hypothetical
Mf|99	NC_006055.1:396957	1	*	*	*	IG	-
Mf|23	NC_006055.1:399947	1	*	*	*	EX	hypothetical
Mf|57	NC_006055.1:400304	-1	*	*	*	EX	hypothetical
Mf|58	NC_006055.1:407328	-1	*	*	*	EX	hypothetical
Mf|59	NC_006055.1:421014	1	*	*	*	EX	hypothetical
Mf|11	NC_006055.1:422354	-1	*	*	*	IG	-
Mf|99	NC_006055.1:437601	-2	*	*	-	IG	-
Mf|55	NC_006055.1:453094	-3	*	*	*	EX	cell
Mf|14	NC_006055.1:454295	1	*	*	*	EX	hypothetical
Mf|6	NC_006055.1:455904	2	*	*	*	IG	-
Mf|83	NC_006055.1:473827	1	*	*	*	EX	class
Mf|76	NC_006055.1:476372	-1	*	*	*	EX	translation
Mf|65	NC_006055.1:489521	1	*	*	*	EX	ATPase
Mf|76	NC_006055.1:499619	2	*	*	*	EX	trehalose/sucrose/beta-glucoside
Mf|92	NC_006055.1:523043	-1	*	*	-	IG	-
Mf|76	NC_006055.1:523043	-1	*	*	-	IG	-
Mf|99	NC_006055.1:523043	-3	*	*	*	IG	-
Mf|14	NC_006055.1:523043	-1	*	*	-	IG	-
Mf|85	NC_006055.1:523043	-1	*	*	-	IG	-
Mf|56	NC_006055.1:523043	-1	*	*	-	IG	-
Mf|83	NC_006055.1:523043	-1	*	*	-	IG	-
Mf|68	NC_006055.1:528050	-3	*	*	*	EX	histone-like/ribosomal-like
Mf|42	NC_006055.1:528050	-3	*	*	*	EX	histone-like/ribosomal-like
Mf|65	NC_006055.1:528050	3	*	*	*	EX	histone-like/ribosomal-like
Mf|55	NC_006055.1:528050	-3	*	*	*	EX	histone-like/ribosomal-like
Mf|12	NC_006055.1:528050	-3	*	*	*	EX	histone-like/ribosomal-like
Mf|23	NC_006055.1:528050	-3	*	*	*	EX	histone-like/ribosomal-like
Mf|6	NC_006055.1:528050	3	*	*	*	EX	histone-like/ribosomal-like
Mf|19	NC_006055.1:528050	-3	*	*	*	EX	histone-like/ribosomal-like
Mf|41	NC_006055.1:528050	-3	*	*	*	EX	histone-like/ribosomal-like
Mf|56	NC_006055.1:528050	-3	*	*	*	EX	histone-like/ribosomal-like
Mf|47	NC_006055.1:528050	-3	*	*	*	EX	histone-like/ribosomal-like
Mf|99	NC_006055.1:529274	-3	*	*	-	IG	-

Mf|57	NC_006055.1:529274	-4	*	*	-	IG	-
Mf|23	NC_006055.1:533891	1	*	*	-	IG	-
Mf|39	NC_006055.1:533891	1	*	*	-	IG	-
Mf|56	NC_006055.1:533891	-1	*	*	-	IG	-
Mf|11	NC_006055.1:533891	-1	*	*	-	IG	-
Mf|14	NC_006055.1:533891	1	*	*	-	IG	-
Mf|41	NC_006055.1:533891	-1	*	*	-	IG	-
Mf|99	NC_006055.1:548681	-1	*	*	-	IG	-
Mf|12	NC_006055.1:570619	-1	*	*	*	EX	hypothetical
Mf|57	NC_006055.1:577206	1	*	*	*	EX	hypothetical
Mf|14	NC_006055.1:577823	-9	*	*	*	EX	hypothetical
Mf|67	NC_006055.1:577890	1	*	*	*	EX	hypothetical
Mf|41	NC_006055.1:596911	1	*	*	*	IG	-
Mf|76	NC_006055.1:598630	1	*	*	*	EX	hypothetical
Mf|41	NC_006055.1:610523	1	*	*	*	IG	-
Mf|58	NC_006055.1:610523	2	*	*	*	IG	-
Mf|55	NC_006055.1:610523	1	*	*	*	IG	-
Mf|50	NC_006055.1:617172	-78	-	-	*	EX	ATP-dependent
Mf|42	NC_006055.1:640996	1	*	*	*	EX	deoxynucleoside
Mf|14	NC_006055.1:668848	1	*	*	*	IG	-
Mf|49	NC_006055.1:668848	-1	*	*	*	IG	-
Mf|42	NC_006055.1:668848	-1	*	*	*	IG	-
Mf|62	NC_006055.1:676726	3	*	*	*	EX	formamidopyrimidine-DNA
Mf|39	NC_006055.1:703756	-122	-	-	*	EX	hypothetical
Mf|19	NC_006055.1:704646	1	*	*	*	EX	beta-lactamase
Mf|19	NC_006055.1:709236	-1	*	*	*	IG	-
Mf|67	NC_006055.1:715252	1	*	*	*	EX	hypothetical
Mf|41	NC_006055.1:715816	-1	*	*	*	EX	hypothetical
Mf|19	NC_006055.1:719113	-1	*	*	*	EX	HAD-superfamily
Mf|49	NC_006055.1:723905	1	*	*	*	IG	-
Mf|39	NC_006055.1:727009	1	*	*	*	EX	substrate
Mf|31	NC_006055.1:727009	1	*	*	*	EX	substrate
Mf|47	NC_006055.1:727917	-1	*	*	*	IG	-
Mf|23	NC_006055.1:734522	-1	*	*	*	EX	cardiolipin
Mf|56	NC_006055.1:739216	-1	*	*	*	EX	polypeptide
Mf|14	NC_006055.1:749645	-1	*	*	*	IG	-
Mf|93	NC_006055.1:751392	-1	*	*	*	EX	multidrug
Mf|58	NC_006055.1:752865	1	*	*	*	IG	-
Mf|92	NC_006055.1:754690	-1	*	*	*	EX	RNA
Mf|56	NC_006055.1:773113	-1	*	*	*	EX	ribose
Mf|58	NC_006055.1:773484	-1	*	*	*	EX	ribose
Mf|31	NC_006055.1:775316	3	*	*	*	EX	ribose
Mf|39	NC_006055.1:775316	3	*	*	*	EX	ribose
Mf|26	NC_006055.1:778327	-1	*	*	*	IG	-
Mf|85	NC_006055.1:785039	5	*	*	*	EX	S-adenosylmethionine:2-demethylmenaquinone
Mf|56	NC_006055.1:785568	1	*	*	*	EX	S-adenosylmethionine:2-demethylmenaquinone
Se|4	NC_005005.1:54	1	-	-	*	IG	-
Se|7	NC_005005.1:54	1	-	-	*	IG	-
Se|2	NC_005005.1:54	1	-	-	*	IG	-
Se|11	NC_005005.1:54	1	-	-	*	IG	-
Se|3	NC_005005.1:54	1	-	-	*	IG	-
Se|12	NC_005005.1:54	1	-	-	*	IG	-
Se|8	NC_005005.1:54	1	-	-	*	IG	-
Se|10	NC_005005.1:54	1	-	-	*	IG	-
Se|13	NC_005005.1:54	1	-	-	*	IG	-
Se|13	NC_005005.1:3346	-12	*	*	*	EX	hypothetical
Se|4	NC_005005.1:10527	1	*	*	*	IG	-
Se|75	NC_005005.1:10527	1	*	*	*	IG	-
Se|50	NC_004461.1:51507	-15481	-	-	*	IG	-

Se|13	NC_004461.1:75823	-1876	-	-	*	EX	transposase
Se|10	NC_004461.1:133408	1	*	*	*	IG	-
Se|67	NC_004461.1:373396	-2	*	*	-	IG	-
Se|39	NC_004461.1:462055	20	-	-	*	EX	hypothetical
Se|39	NC_004461.1:485768	1	*	*	*	EX	hypothetical
Se|34	NC_004461.1:485768	1	*	*	*	EX	hypothetical
Se|66	NC_004461.1:578337	-1	*	*	*	EX	hypothetical
Se|65	NC_004461.1:578337	-1	*	*	*	EX	hypothetical
Se|10	NC_004461.1:740878	-1	-	-	*	IG	-
Se|8	NC_004461.1:740878	-1	-	-	*	IG	-
Se|2	NC_004461.1:796289	2	*	*	*	EX	manganese
Se|12	NC_004461.1:912691	2	*	*	*	IG	-
Se|39	NC_004461.1:948449	-132	-	-	*	EX	transcription
Se|67	NC_004461.1:1206471	-24	*	*	*	EX	elastin
Se|39	NC_004461.1:1208360	-1	*	*	*	EX	ATP-dependent
Se|75	NC_004461.1:1501509	-3601	-	-	*	IG	-
Se|8	NC_004461.1:1577662	-1	*	*	*	IG	-
Se|67	NC_004461.1:1592951	1	*	*	*	IG	-
Se|75	NC_004461.1:1593739	1	*	*	*	IG	-
Se|70	NC_004461.1:1594245	-1	*	*	-	IG	-
Se|66	NC_004461.1:1666743	1	*	*	*	EX	hypothetical
Se|50	NC_004461.1:1717590	-1	*	*	*	EX	threonine
Se|12	NC_004461.1:1813275	1	*	*	*	IG	-
Se|11	NC_004461.1:1843381	1	*	*	*	EX	PTS
Se|69	NC_004461.1:1877753	-1	*	*	*	EX	acriflavin
Se|34	NC_004461.1:1917547	-1	*	*	-	EX	hypothetical
Se|34	NC_004461.1:1917550	-1	-	-	*	EX	hypothetical
Se|34	NC_004461.1:2217486	-5	-	-	*	IG	-
Se|39	NC_004461.1:2217486	-5	-	-	*	IG	-
Se|66	NC_004461.1:2401344	-2	*	*	*	EX	poly
Se|65	NC_004461.1:2401344	-2	*	*	*	EX	poly
Se|12	NC_004461.1:2467399	9	*	*	*	IG	-
Vc|57	NZ_AAUT01000005.1:992	-1	*	*	-	IG	-
Vc|63	NZ_AAUT01000016.1:3729	-1	*	*	*	EX	hypothetical
Vc|29	NZ_AAUT01000075.1:4429	1	*	*	-	IG	-
Vc|49	NZ_AAUT01000120.1:4583	-1	*	*	-	EX	hypothetical
Vc|53	NZ_AAUT01000046.1:6590	-12064	-	-	*	EX	sensor
Vc|23	NZ_AAUT01000026.1:7390	-13	*	*	*	EX	hypothetical
Vc|58	NZ_AAUT01000027.1:10961	-4	*	*	*	EX	long-chain
Vc|65	NZ_AAUT01000047.1:11646	-1	*	*	*	EX	superoxide
Vc|39	NZ_AAUT01000046.1:19581	-21	*	*	*	EX	RTX
Vc|32	NZ_AAUT01000059.1:19844	-1	-	-	*	IG	-
Vc|70	NZ_AAUT01000023.1:23212	-40	-	-	*	EX	sigma-54
Vc|47	NZ_AAUT01000053.1:25517	1	*	*	-	EX	hypothetical
Vc|53	NZ_AAUT01000018.1:31753	16	-	-	*	IG	-
Vc|26	NZ_AAUT01000041.1:32998	-10	*	*	*	EX	hypothetical
Vc|25	NZ_AAUT01000041.1:32998	-10	*	*	-	EX	hypothetical
Vc|7	NZ_AAUT01000011.1:33355	-1	*	*	*	IG	-
Vc|51	NZ_AAUT01000001.1:34071	-12	*	*	*	EX	hydroxymethylglutaryl-coenzyme
Vc|4	NZ_AAUT01000033.1:35325	-1	*	*	*	EX	cell
Vc|1	NZ_AAUT01000005.1:62972	-6424	-	-	*	IG	-
Vc|9	NZ_AAUT01000005.1:87636	-536	-	-	*	IG	-
